# Intravenous immunoglobulin for the treatment of Kawasaki disease

**DOI:** 10.1002/14651858.CD014884.pub2

**Published:** 2023-01-25

**Authors:** Cathryn Broderick, Shinobu Kobayashi, Maiko Suto, Shuichi Ito, Tohru Kobayashi

**Affiliations:** Usher InstituteUniversity of EdinburghEdinburghUK; Department of Social MedicineNational Center for Child Health and DevelopmentTokyoJapan; Department of Health PolicyNational Center for Child Health and DevelopmentTokyoJapan; Department of PediatricsGraduate School of Medicine, Yokohama City UniversityYokohamaJapan; Department of Development StrategyNational Center for Child Health and DevelopmentTokyoJapan

**Keywords:** Child, Humans, Acute Coronary Syndrome, Aspirin, Aspirin/adverse effects, Fever, Fever/drug therapy, Fever/etiology, Immunoglobulins, Intravenous, Immunoglobulins, Intravenous/adverse effects, Inflammation, Mucocutaneous Lymph Node Syndrome, Mucocutaneous Lymph Node Syndrome/drug therapy, Prednisolone, Prednisolone/therapeutic use

## Abstract

**Background:**

Kawasaki disease (KD) is an acute systemic vasculitis (inflammation of the blood vessels) that mainly affects children. Symptoms include fever, chapped lips, strawberry tongue, red eyes (bulbar conjunctival injection), rash, redness, swollen hands and feet or skin peeling; and enlarged cervical lymph nodes. High fevers and systemic inflammation characterise the acute phase. Inflammation of the coronary arteries causes the most serious complication of the disease, coronary artery abnormalities (CAAs). The primary treatment is intravenous immunoglobulin (IVIG) and acetylsalicylic acid (ASA/aspirin), with doses and regimens differing between institutions. It is important to know which regimens are the safest and most effective in preventing complications.

**Objectives:**

To evaluate the efficacy and safety of IVIG in treating and preventing cardiac consequences of Kawasaki disease.

**Search methods:**

The Cochrane Vascular Information Specialist searched the Cochrane Vascular Specialised Register, CENTRAL, MEDLINE, Embase, and CINAHL databases, and the World Health Organization International Clinical Trials Registry Platform and ClinicalTrials.gov trials registers to 26 April 2022.

**Selection criteria:**

We included randomised controlled trials (RCTs) investigating the use of IVIG for the treatment of KD. We included studies involving treatment for initial or refractory KD, or both.

**Data collection and analysis:**

We used standard Cochrane methods. Our primary outcomes were incidence of CAAs and incidence of any adverse effects after treatment. Our secondary outcomes were acute coronary syndromes, duration of fever, need for additional treatment, length of hospital stay, and mortality. We used GRADE to assess the certainty of the evidence for each outcome.

**Main results:**

We identified 31 RCTs involving a total of 4609 participants with KD. Studies compared IVIG with ASA, another dose or regimen of IVIG, prednisolone, or infliximab. The majority of studies reported on primary treatment, so those results are reported below. A limited number of studies investigated secondary or tertiary treatment in IVIG‐resistant patients. Doses and regimens of IVIG infusion varied between studies, and all studies had some concerns related to risk of bias.

**Primary treatment with IVIG compared to ASA for people with KD**

Compared to ASA treatment, IVIG probably reduces the incidence of CAAs in people with KD up to 30 days (odds ratio (OR) 0.60, 95% confidence interval (CI) 0.41 to 0.87; 11 studies, 1437 participants; moderate‐certainty evidence). The individual studies reported a range of adverse effects, but there was little to no difference in numbers of adverse effects between treatment groups (OR 0.57, 95% CI 0.17 to 1.89; 10 studies, 1376 participants; very low‐certainty evidence). There was limited evidence for the incidence of acute coronary syndromes, so we are uncertain of any effects. Duration of fever days from treatment onset was probably shorter in the IVIG group (mean difference (MD) −4.00 days, 95% CI −5.06 to −2.93; 3 studies, 307 participants; moderate‐certainty evidence). There was little or no difference between groups in need for additional treatment (OR 0.27, 95% CI 0.05 to 1.57; 3 studies, 272 participants; low‐certainty evidence). No study reported length of hospital stay, and no deaths were reported in either group.

**Primary treatment with IVIG compared to different infusion regimens of IVIG for people with KD**

Higher‐dose regimens of IVIG probably reduce the incidence of CAAs compared to medium‐ or lower‐dose regimens of IVIG up to 30 days (OR 0.60, 95% CI 0.40 to 0.89; 8 studies, 1824 participants; moderate‐certainty evidence). There was little to no difference in the number of adverse effects between groups (OR 1.11, 95% CI 0.52 to 2.37; 6 studies, 1659 participants; low‐certainty evidence). No study reported on acute coronary syndromes. Higher‐dose IVIG may reduce the duration of fever compared to medium‐ or lower‐dose regimens (MD −0.71 days, 95% CI −1.36 to −0.06; 4 studies, 992 participants; low‐certainty evidence). Higher‐dose regimens may reduce the need for additional treatment (OR 0.29, 95% CI 0.10 to 0.88; 4 studies, 1125 participants; low‐certainty evidence). We did not detect a clear difference in length of hospital stay between infusion regimens (MD −0.24, 95% CI −0.78 to 0.30; 3 studies, 752 participants; low‐certainty evidence). One study reported mortality, and there was little to no difference detected between regimens (moderate‐certainty evidence).

**Primary treatment with IVIG compared to prednisolone for people with KD**

The evidence comparing IVIG with prednisolone on incidence of CAA is very uncertain (OR 0.60, 95% CI 0.24 to 1.48; 2 studies, 140 participants; very low‐certainty evidence), and there was little to no difference between groups in adverse effects (OR 4.18, 95% CI 0.19 to 89.48; 1 study; 90 participants; low‐certainty evidence). We are very uncertain of the impact on duration of fever, as two studies reported this outcome differently and showed conflicting results. One study reported on acute coronary syndromes and mortality, finding little or no difference between groups (low‐certainty evidence). No study reported the need for additional treatment or length of hospital stay.

**Authors' conclusions:**

The included RCTs investigated a variety of comparisons, and the small number of events observed during the study periods limited detection of effects. The certainty of the evidence ranged from moderate to very low due to concerns related to risk of bias, imprecision, and inconsistency. The available evidence indicated that high‐dose IVIG regimens are probably associated with a reduced risk of CAA formation compared to ASA or medium‐ or low‐dose IVIG regimens. There were no clinically significant differences in incidence of adverse effects, which suggests there is little concern about the safety of IVIG. Compared to ASA, high‐dose IVIG probably reduced the duration of fever, but there was little or no difference detected in the need for additional treatment. Compared to medium‐ or low‐dose IVIG, there may be reduced duration of fever and reduced need for additional treatment. We were unable to draw any conclusions regarding acute coronary syndromes, mortality, or length of hospital stay, or for the comparison IVIG versus prednisolone. Our findings are in keeping with current guideline recommendations and evidence from long‐term epidemiology studies.

## Plain language summary

**Immunoglobulin treatment for Kawasaki disease**

**Key message**

In children with Kawasaki disease, high‐dose intravenous immunoglobulin (IVIG) regimens: • probably reduce the risk of coronary artery abnormalities formation compared to aspirin or medium‐ or low‐dose IVIG regimens; • show no clear safety concerns; • probably reduce the duration of fever, but there was little or no difference detected in the need for additional treatment compared to aspirin; • may reduce the duration of fever and need for additional treatment compared to medium‐ or low‐dose IVIG.

**Why is this question important?**

Kawasaki disease is a condition that causes blood vessels to become swollen and inflamed. Symptoms include high temperature (fever) along with chapped lips, strawberry tongue (swollen, bumpy red tongue), red eyes, a rash, redness and swelling of hands and feet or skin peeling; and swollen glands in the neck. It is most often seen in young children. Swelling and inflammation of the vessels which supply blood to the heart (coronary arteries) is the most serious complication of the disease, causing coronary artery abnormalities. These abnormalities can result in acquired heart disease and sometimes death. Fast diagnosis and treatment can prevent these complications. Intravenous immunoglobulin and aspirin are the main medicines used to treat Kawasaki disease. Different combinations, doses, or timings (regimens) of these medicines are used to treat patients. It is important to know which treatments and regimens are the safest and most effective in preventing heart complications.

**What did we do?**

We searched for randomised controlled trials (a type of study where participants are randomly assigned to one of two or more treatment groups) that compared treatment of Kawasaki disease with IVIG to treatment with another medicine or regimen. We found 31 studies with a total of 4609 participants. The studies compared IVIG with aspirin, another regimen of IVIG, infliximab, or prednisolone. All studies reported on coronary artery abnormalities, but they did not all report on our other outcomes of interest: adverse effects, acute coronary syndromes (such as a heart attack), duration of fever, need for additional treatment, length of hospital stay, and mortality. Where appropriate, we combined data from the included studies.

**What did we find?**

We looked at studies that compared IVIG with aspirin. Our results showed that children who received IVIG probably developed fewer coronary artery abnormalities than children treated with aspirin alone. Reporting of adverse effects varied between studies, but there was little or no difference in the number of adverse effects between groups. Acute coronary syndromes were poorly reported, so we cannot say if IVIG affected this. IVIG treatment probably reduced the duration of fever compared to aspirin alone. There was little or no difference in the need for additional treatment between IVIG and aspirin groups, and no study reported on the length of hospital stay. No deaths were reported in either group.

We looked at studies that compared high‐dose IVIG regimens with medium‐ or low‐dose regimens. Our results showed that children who received high‐dose regimens probably developed fewer abnormalities. There was no apparent difference in the number of adverse effects between the high and medium‐ or low‐dose groups. Acute coronary syndromes were poorly reported, with little or no difference seen between groups. Higher‐dose regimens may reduce the duration of fever and need for additional treatment compared to medium‐ and lower‐dose regimens. We did not find a clear difference between regimens in length of hospital stay. One death was reported in one study, so we cannot say for sure if one regimen was better than another for this outcome.

We also looked at studies that compared IVIG with prednisolone. The evidence was very uncertain for number of coronary artery abnormalities and duration of fever, and there was little or no difference between groups in adverse effects, acute coronary syndromes, and deaths. Results for all outcomes were limited by the small number of children with Kawasaki disease involved. No study reported on need for additional treatment or length of hospital stay.

**What are the limitations of the evidence?**

Our confidence in the evidence provided from the included studies differed between outcomes and ranged from very low to moderate, due to concerns about how some of the studies were carried out, differences in the regimens used, and there were often only small numbers of events and participants providing data. Our findings are in keeping with current guideline recommendations and evidence from long‐term studies involving large numbers of people.

**How up‐to‐date is this evidence?**

The evidence is current to April 2022.

## Summary of findings

**Summary of findings 1:** Primary treatment with intravenous immunoglobulin (IVIG) compared to acetylsalicylic acid (ASA) for people with Kawasaki disease

**Intravenous immunoglobulin (IVIG) versus acetylsalicylic acid (ASA) for the primary treatment of Kawasaki disease**						
**Patient or population:** people undergoing initial treatment for KD **Settings:** hospital **Intervention:** IVIG^a^ **Comparison:** ASA^b^						
**Outcomes**	Anticipated absolute effects* (95% CI)		**Relative effect (95% CI)**	**No. of participants (studies)**	**Certainty of the evidence (GRADE)**	**Comments**
	**Risk with ASA**	**Risk with IVIG**				
**Incidence of CAAs** (up to 30 days)	**Study population**		**OR 0.60** (0.41 to 0.87)	1437 (11 RCTs)	⊕⊕⊕⊝ **Moderate**^c^	It is likely that the incidence of CAA was reduced in the IVIG group compared to the ASA group.
	**295 per 1000**	**201 per 1000** (147 to 267)				
**Incidence of any adverse effects after treatment initiation** (from 30 days to 30 months)	**Study population**		**OR 0.57** (0.17 to 1.89)	1376 (10 RCTs)	⊕⊝⊝⊝ **Very low**^d^	There was little or no difference in the numbers of adverse effects detected. See Table 2 for adverse effects reported by study.
	**67 per 1000**	**39 per 1000** (12 to 120)				
**Acute coronary syndrome** (such as MI or coronary thrombus) (to 30 months)	**Study population**		‐	202 (2 RCTs)	⊕⊕⊝⊝ **Low**^e^	Newburger 1986 reported acute coronary syndromes: 30 months 2/17 participants in the ASA group had coronary thrombus compared to 0/7 in the IVIG group. Matsushima 1985 confirmed no acute coronary events occurred.
	See comment	‐				
**Duration of fever** (days) (acute phase)	The mean duration of fever from treatment onset across ASA group ranged from **6 to 7.9 days**.	The mean duration of fever from treatment onset in the IVIG group was **4 days lower** (5.06 lower to 2.93 lower).	‐	307 (3 RCTs)	⊕⊕⊕⊝ **Moderate**^f^	It is likely that duration of fever was reduced in the IVIG group compared to the ASA group.
**Need for additional treatment** (up to 30 or 60 days)	**Study population**		**OR 0.27** (0.05 to 1.57)	272 (3 RCTs)	⊕⊕⊝⊝ **Low**^g^	There was little or no difference in the need for additional treatment between groups.
	**36 per 1000**	**10 per 1000** (2 to 55)				
**Length of hospital stay** (days)	See comment	‐	‐	‐	‐	None of the studies comparing IVIG with ASA reported on length of hospital stay.
**Mortality (all‐cause)**	**Study population**		‐	‐	⊕⊕⊝⊝ **Low**^e^	Details on the incidence of mortality were reported by 2 studies, with no deaths occurring in either study.
	See comment	‐				
*The risk in the intervention group (and its 95% confidence interval) is based on the assumed risk in the comparison group and the **relative effect** of the intervention (and its 95% CI). **ASA:** acetylsalicylic acid; **CAA:** coronary artery abnormality; **CI:** confidence interval; **IVIG:** intravenous immunoglobulin; **KD:** Kawasaki disease; **MI:** myocardial infarction; **OR:** odds ratio; **RCT:** randomised controlled trial						
**GRADE Working Group grades of evidence** **High certainty:** we are very confident that the true effect lies close to that of the estimate of the effect. **Moderate certainty:** we are moderately confident in the effect estimate: the true effect is likely to be close to the estimate of the effect, but there is a possibility that it is substantially different. **Low certainty:** our confidence in the effect estimate is limited: the true effect may be substantially different from the estimate of the effect. **Very low certainty:** we have very little confidence in the effect estimate: the true effect is likely to be substantially different from the estimate of effect.						

^a^IVIG total dose ranged from 100 mg/kg to 2 g/kg, via either single infusion or multiple infusions over three to five days. See Characteristics of included studies tables for specific details. All studies administered ASA to both arms, except for Furusho 1991B, where one of two IVIG study arms did not receive ASA. ^b^Most studies administered ASA initially at 30 mg/kg/day, except for Newburger 1986, who used 100 mg/kg/day every 6 hours to day 14 of illness, and Okuni 1987A, Okuni 1987B, and Onouchi 1988, who used 50 mg/kg/day. ^c^We downgraded by one level due to concerns related to risk of bias. ^d^We downgraded by one level due to concerns related to risk of bias, one level for inconsistency (I^2^ = 73%), and one level for imprecision (small number of events, and confidence intervals include appreciable benefit or harm). ^e^We downgraded by one level due to concerns related to risk of bias and one level for imprecision (small numbers of participants and events). ^f^We downgraded by a total of one level due to concerns related to risk of bias and imprecision (small number of participants). ^g^We downgraded by one level due to concerns related to risk of bias and one level for imprecision (small numbers of participants and events, and confidence intervals include appreciable benefit or harm).

**1:** Adverse events ‐ primary treatment with IVIG versus ASA

**Study**	**IVIG^a^**	**ASA^a^**	**Comments**
**Furusho 1984**	1/46 chills and fever	1/47 liver disorder	IVIG discontinued, and ASA changed to IVIG.
**Furusho 1991B**			Reports no SAE, AE not detailed
**Matsushima 1985**	1/17 pericardial effusion	5/15 pericardial effusion	
**Nagashima 1987**	8/69 chills and fever 1/69 urticaria	0/67	In all cases, symptoms were transient and disappeared in a short time.
**Newburger 1986**	0 SAE 3/84 mild congestive heart failure 1/84 shaking/itching 1/84 sepsis 1/84 rash, fever, lymphadenopathy and splenomegaly 1/84 neutropenia and splenomegaly	4/84 mild congestive heart failure 1/84 neutropenia	
**Ogawa 1987**	21/63 liver disorder	6/54 liver disorder	
**Ogino 1987**	0/50	6/42 liver disorder	
**Okuni 1987A**	0/139	0/75	
**Okuni 1987B**	0/196	0/99	
**Onouchi 1988**	1/97 erythema (200 mg/kg arm)	8/48 liver disorder 1/48 nasal haemorrhage	
**Yabiku 1989**	4/48 liver disorder	6/36 liver disorder	

AE: adverse event ASA: acetylsalicylic acid IVIG: intravenous immunoglobulin SAE: serious adverse event ^a^See Characteristics of included studies tables for doses given.

**Summary of findings 2:** Primary treatment with intravenous immunoglobulin (IVIG) compared to different infusion regimens of IVIG for people with Kawasaki disease

**High‐dose versus medium‐ or low‐dose intravenous immunoglobulin infusion (IVIG) regimens for the primary treatment of Kawasaki disease**						
**Patient or population:** people undergoing initial treatment for KD **Settings:** hospital **Intervention:** IVIG high‐dose regimens^a^ **Comparison:** IVIG medium‐ or low‐dose regimens^b^						
**Outcomes**	Anticipated absolute effects* (95% CI)		**Relative effect (95% CI)**	**No. of participants (studies)**	**Certainty of the evidence (GRADE)**	**Comments**
	**Risk with medium‐ or low‐dose IVIG**	**Risk with high‐dose IVIG**				
**Incidence of CAAs** (up to 30 days)	**Study population**		**OR 0.60** (0.40 to 0.89)	1824 (8 RCTs)	⊕⊕⊕⊝ **Moderate**^c^	It is likely that the incidence of CAAs is reduced in the high‐dose infusion regimens compared to the medium‐ or low‐dose regimens.
	**204 per 1000**	**133 per 1000** (93 to 186)				
**Incidence of any adverse effects after treatment initiation** (follow‐up ranged from 30 days to 5 years)	**Study population**		**OR 1.11** (0.52 to 2.37)	1659 (6 RCTs)	⊕⊕⊝⊝ **Low**^d^	There was little or no difference in the number of adverse effects detected. See Table 4 for adverse effects reported by study.
	**19 per 1000**	**21 per 1000** (10 to 44)				
**Acute coronary syndrome** (such as MI or coronary thrombus) (up to 7 weeks)	**Study population**		‐	‐	‐	No studies reported this outcome.
	See comment	‐				
**Duration of fever** (days) (acute phase)	The mean duration of fever in the medium‐ or low‐dose infusion regimens ranged from **5 days to 10.3 days**.	The mean duration of fever was **0.71 days lower** (1.36 lower to 0.06 lower) in the high‐dose infusion regimens.	‐	992 (4 RCTs)	⊕⊕⊝⊝ **Low**^e^	Duration of fever may be slightly reduced in the high‐dose regimens compared to the medium‐ or low‐dose regimens.
**Need for additional treatment** (up to 6 months)	**Study population**		**OR 0.29** (0.10 to 0.88)	1125 (4 RCTs)	⊕⊕⊝⊝ **Low**^f^	Need for additional treatment may be slightly reduced in the high‐dose regimens compared to the medium‐ or low‐dose regimens.
	**116 per 1000**	**37 per 1000** (13 to 103)				
**Length of hospital stay** (days) (up to 28 days)	The mean length of hospital stay (days) ranged from **8.3 days to 18.95 days** across medium‐ and low‐dose infusion regimens.	The mean length of hospital stay was **0.24 days lower** (0.78 lower to 0.3 higher) in the high‐dose infusion regimens.	‐	752 (3 RCTs)	⊕⊕⊝⊝ **Low**^g^	There was little to no difference detected in the length of hospital stay between regimens.
**Mortality (all‐cause)** (up to 7 weeks)	**Study population**		‐	549 (1 RCT)	⊕⊕⊕⊝ **Moderate**^h^	Newburger 1991 reported 1 death in the subacute phase of the 400 mg/kg/day for 4 days group. The cause of death was a giant aneurysm. The remaining studies did not report mortalities.
	See comment	‐				
*The risk in the intervention group (and its 95% confidence interval) is based on the assumed risk in the comparison group and the **relative effect** of the intervention (and its 95% CI). **CAA:** coronary artery abnormality; **CI:** confidence interval; **IVIG:** intravenous immunoglobulin; **KD:** Kawasaki disease; **MI:** myocardial infarction; **OR:** odds ratio; **RCT:** randomised controlled trial						
**GRADE Working Group grades of evidence** **High certainty:** we are very confident that the true effect lies close to that of the estimate of the effect. **Moderate certainty:** we are moderately confident in the effect estimate: the true effect is likely to be close to the estimate of the effect, but there is a possibility that it is substantially different. **Low certainty:** our confidence in the effect estimate is limited: the true effect may be substantially different from the estimate of the effect. **Very low certainty:** we have very little confidence in the effect estimate: the true effect is likely to be substantially different from the estimate of effect.						

^a^We included studies that compared different doses or infusion regimens of IVIG for the primary treatment of KD. We defined high dose as single‐ or multiple‐infusion regimens of over 1600 mg/kg total IVIG. ^b^We defined medium dose to be single‐ or multiple‐infusion regimens of 1600 mg/kg to 1000 mg/kg total IVIG, and low dose to be single‐ or multiple‐infusion regimens of less than 1000 mg/kg total IVIG. ^c^We downgraded by one level due to concerns related to risk of bias. ^d^We downgraded by one level due to concerns related to risk of bias and one level for imprecision (small numbers of events, and confidence intervals include appreciable benefit or harm). ^e^We downgraded by one level due to concerns related to risk of bias and one level for inconsistency (I^2^ = 71%). ^f^We downgraded by one level due to concerns related to risk of bias and one level for inconsistency (I^2^ = 69%). ^g^We downgraded by a total of two levels due to concerns related to risk of bias and imprecision (confidence intervals include appreciable benefit or harm). ^h^We downgraded by one level for imprecision (small number of events reported).

**2:** Adverse events ‐ primary treatment with IVIG versus IVIG

**Study**	**IVIG**	**IVIG**	**IVIG**	**Comments**
**Barron 1990**	10/22 1 g/kg single 4 mild flushing, chills, and nausea and vomiting, mild hypotension, morbilliform rash 6/22 pericardial effusion	6/22 400 mg/kg/day for 4 days 3 shaking chills, chills and noisy breathing, headache, flushing, and abdominal cramping 3/22 pericardial effusion	‐	Numbers of participants with pericardial effusion added separately to other AE, as it is not clear from text if these were different participants to those reporting other AE.
**Furusho 1991A**	0/41 100 mg/kg/day for 5 days	0/51 200 mg/kg/day for 5 days	0/53 400 mg/kg/day for 5 days	No serious AE were detected. Unclear if this means no AE or not reported
**Harada 1989**	2/125 100 mg/kg/day for 5 days rash, mild hypotension	1/117 400 mg/kg/day for 5 days anaphylactic shock	‐	
**He 2021**	1/129 1 g/kg/day for 2 days chickenpox	0/132 2 g/kg single	0/138 1 g/kg single	1/129 recovered without complications.
**Morikawa 1994**	13/310 200 mg/kg/day for 5 days	7/156 400 mg/kg/day for 5 days	‐	All reported AE were mild to moderate and included fever, rash, shivering, peripheral cyanosis, hepatic dysfunction.
**Newburger 1991**	9/273 2 g/kg single 6/273 new or worsening congestive heart failure 1/273 infusion was SC not IV – oedema and blistering 1/273 generalised oedema 1/273 nasal congestion, cough, nausea	6/276 400 mg/kg/day for 4 days 3/276 new or worsening congestive heart failure 2/276 had pruritis 1/276 had generalised oedema	‐	
**Nishihara 1988**	‐	‐	‐	Did not report
**Onouchi 1988**	1/49 200 mg/kg/day for 3 days erythema	0/48 400 mg/kg/day for 3 days	‐	
**Onouchi 1992**	0/57 100 mg/kg/day for 5 days	0/52 200 mg/kg/day for 5 days	1/56 400 mg/kg/day for 5 days nausea	
**Qin 2006**	2/122 2 g/kg single 2 cases of rash, nausea, and abdominal pain	2/120 1 g/kg single 2 cases of rash, nausea, and abdominal pain	‐	Related to an allergic reaction to IVIG
**Sakata 2007**	‐	‐	‐	Did not report
**Sato 1995**	0/72 2 g/kg single	0/73 400 mg/kg/day for 5 days	‐	Reported that there were no AE to IVIG treatment in either group

AE: adverse effects IV: intravenous IVIG: intravenous immunoglobulin SC: subcutaneous

**Summary of findings 3:** Primary treatment with intravenous immunoglobulin (IVIG) compared to prednisolone for people with Kawasaki disease

**Intravenous immunoglobulin (IVIG) versus prednisolone for the primary treatment of Kawasaki disease**						
**Patient or population:** people undergoing initial treatment for KD **Settings:** hospital **Intervention:** IVIG^a^ **Comparison:** prednisolone^b^						
**Outcomes**	Anticipated absolute effects* (95% CI)		**Relative effect (95% CI)**	**No. of participants (studies)**	**Certainty of the evidence (GRADE)**	**Comments**
	**Risk with prednisolone**	**Risk with IVIG**				
**Incidence of CAAs** (acute phase)	**Study population**		**OR 0.60** (0.24 to 1.48)	140 (2 RCTs)	⊕⊝⊝⊝ **Very low**^c^	The evidence is very uncertain.
	**200 per 1000**	**130 per 1000** (57 to 270)				
**Incidence of any adverse effects after treatment initiation** (up to 3 months)	**Study population**		**OR 4.18** (0.19 to 89.48)	90 (1 RCT)	⊕⊕⊝⊝ **Low**^d^	There is little to no difference in the incidence of adverse effects.
	0/40 in prednisolone group	Not estimable				
**Acute coronary syndrome** (such as MI or coronary thrombus) (up to 3 months)	**Study population**		‐	90 (1 RCT)	⊕⊕⊝⊝ **Low**^e^	Nonaka 1995 reported an MI in the IVIG group (1/50 vs 0/40 in the prednisolone group).
	See comment	‐				
**Duration of fever** (days)	See comment	‐	‐	140 (2 RCTs)	⊕⊝⊝⊝ **Very low**^f^	We did not pool, as considerable heterogeneity was detected.
**Need for additional treatment**	**Study population**		‐	‐	‐	Neither study comparing IVIG with prednisolone reported on the need for additional treatment.
	See comment	‐				
**Length of hospital stay** (days)	See comment	‐	‐	‐	‐	Neither study comparing IVIG with prednisolone reported on length of hospital stay.
**Mortality (all‐cause)** (up to 30 days)	**Study population**		‐	90 (1 RCT)	⊕⊕⊝⊝ **Low^e^**	Nonaka 1995 reported 1/50 deaths in the IVIG group compared to 0/40 in the prednisolone group. Cause of death was a giant aneurysm, intracranial bleeding, and MI.
	See comment	‐				
*The risk in the intervention group (and its 95% confidence interval) is based on the assumed risk in the comparison group and the **relative effect** of the intervention (and its 95% CI). **CAA:** coronary artery abnormality; **CI:** confidence interval; **IVIG:** intravenous immunoglobulin; **KD:** Kawasaki disease; **MI:** myocardial infarction; **OR:** odds ratio; **RCT:** randomised controlled trial						
**GRADE Working Group grades of evidence** **High certainty:** we are very confident that the true effect lies close to that of the estimate of the effect. **Moderate certainty:** we are moderately confident in the effect estimate: the true effect is likely to be close to the estimate of the effect, but there is a possibility that it is substantially different. **Low certainty:** our confidence in the effect estimate is limited: the true effect may be substantially different from the estimate of the effect. **Very low certainty:** we have very little confidence in the effect estimate: the true effect is likely to be substantially different from the estimate of effect.						

^a^IVIG dose was 400 mg/kg/day IVIG for 3 days, Nonaka 1995, and 1 g/kg/day IVIG for 2 days, Yuan 2000. See Characteristics of included studies tables for specific details. ^b^Prednisolone dose was 2 mg/kg/day for 5 days, Nonaka 1995, or 2 mg/kg/day intravenous methylprednisolone for 5 days, Yuan 2000, then oral prednisolone until C‐reactive protein‐negative. This group did not receive IVIG. ^c^We downgraded by two levels due to concerns related to risk of bias and one level for imprecision (small numbers of events and participants). ^d^We downgraded by one level due to concerns related to risk of bias and one level for imprecision (small numbers of events and participants, very wide confidence interval). ^e^We downgraded by one level due to concerns related to risk of bias and one level for imprecision (small numbers of events and participants). ^f^We downgraded by one level due to concerns related to risk of bias, one level for inconsistency (I^2^ = 82%), and one level for imprecision (small numbers of participants).

## Background

### Description of the condition

Kawasaki disease (KD) is an acute systemic vasculitis (inflammation of the blood vessels) first described in 1967 by Japanese paediatrician Tomisaku Kawasaki that mainly affects children (Kawasaki 1967; Yamaji 2019). The majority of cases are seen in children between six months and five years old, and more often in males than females (21 per 100,000 compared to 15 per 100,000, respectively) (Makino 2019). There is substantial ethnic variation, with the lowest rates seen amongst white children (13.7 per 100,000 children under five) and the highest rates seen in children of Asian descent (29.8 per 100,000 children under five) (Makino 2019), although a Japan‐wide survey reported that KD had increased between 2008 and 2015 (Makino 2015). Data are lacking for black/Hispanic ethnic groups due to too few cases being reported (Holman 2010; Maddox 2015).

There is no specific diagnostic test for KD, with diagnosis being made using clinical criteria and excluding other possible diagnoses. To be diagnosed with KD, individuals must have five or more days of fever as well as four or more of the five principal clinical features (chapped lips, strawberry tongue; bulbar conjunctival injection; rash, redness, and swelling of hands and feet or skin peeling; and enlarged cervical lymph nodes) (see Table 6) (Rife 2020). Individuals who meet the criteria are said to have complete KD (also known as typical or classic KD). Individuals who do not meet all the criteria may be diagnosed as having incomplete KD (also known as atypical KD) (Kobayashi 2020; McCrindle 2017).

**3:** Diagnosis of classic Kawasaki disease*

**Diagnosis of classic Kawasaki disease**
Classic KD is diagnosed in the presence of fever for at least 5 days (the day of fever onset is taken to be the first day of fever) together with at least 4 of the 5 following principal clinical features. In the presence of ≥ 4 principal clinical features, particularly when redness and swelling of the hands and feet are present, the diagnosis of KD can be made with 4 days of fever, although in rare cases experienced clinicians who have treated many patients with KD may establish the diagnosis with 3 days of fever. Erythema and cracking of lips, strawberry tongue, and/or erythema of oral and pharyngeal mucosa Bilateral bulbar conjunctival injection without exudate Rash: maculopapular, diffuse erythroderma, or erythema multiforme‐like Erythema and oedema of the hands and feet in acute phase and/or periungual desquamation in subacute phase Cervical lymphadenopathy (≥ 1.5‐centimetre diameter), usually unilateral
A careful history may reveal that ≥ 1 principal clinical features were present during the illness but had resolved by the time of presentation.
Patients who lack full clinical features of classic KD are often evaluated for incomplete KD. If coronary artery abnormalities are detected, the diagnosis of KD is considered confirmed in most cases.
Laboratory tests typically reveal normal or elevated white blood cell count with neutrophil predominance and elevated acute phase reactants such as C‐reactive protein and erythrocyte sedimentation rate during the acute phase. Low serum sodium and albumin levels, elevated serum liver enzymes, and sterile pyuria can be present. In the second week after fever onset, thrombocytosis is common.
**Other clinical findings may include the following:**
**Cardiovascular**
Myocarditis, pericarditis, valvular regurgitation, shock
Coronary artery abnormalities
Aneurysms of medium‐sized non‐coronary arteries
Peripheral gangrene
Aortic root enlargement
**Respiratory**
Peribronchial and interstitial infiltrates on chest x‐ray
Pulmonary nodules
**Musculoskeletal**
Arthritis, arthralgia (pleocytosis of synovial fluid)
**Gastrointestinal**
Diarrhoea, vomiting, abdominal pain
Hepatitis, jaundice
Gallbladder hydrops
Pancreatitis
**Nervous system**
Extreme irritability
Aseptic meningitis (pleocytosis of cerebrospinal fluid)
Facial nerve palsy
Sensorineural hearing loss
Genitourinary
Urethritis/meatitis, hydrocele
**Other**
Desquamating rash in groin
Retropharyngeal phlegmon
Anterior uveitis by slit lamp examination
Erythema and induration at BCG inoculation site
**The differential diagnosis includes other infectious and non‐infectious conditions, including the following:**
Measles
Other viral infections (e.g. adenovirus, enterovirus)
Staphylococcal and streptococcal toxin‐mediated diseases (e.g. scarlet fever and toxic shock syndrome)
Drug hypersensitivity reactions, including Stevens‐Johnson syndrome
Systemic onset juvenile idiopathic arthritis
**With epidemiologic risk factors:**
Rocky Mountain spotted fever or other rickettsial infections
Leptospirosis

*American Heart Association guidelines (McCrindle 2017). BCG: Bacillus Calmette–Guérin (used in vaccine for prevention of tuberculosis) KD: Kawasaki disease

Kawasaki disease is usually triphasic with an acute, subacute, and convalescent phase. The acute phase is characterised by high fevers (lasting from seven to 14 days if untreated), and systemic inflammation in the medium‐sized arteries, multiple organs, and tissues, resulting in the following common clinical findings: liver (hepatocyte damage), lung (interstitial pneumonitis), gastrointestinal tract (abdominal pain, vomiting, diarrhoea, gallbladder hydrops), meninges (aseptic meningitis, irritability), heart (myocarditis, pericarditis, valvulitis), urinary tract (pyuria), pancreas (pancreatitis), and lymph nodes (lymphadenopathy) (McCrindle 2017). Inflammation of the coronary arteries causes the most serious complication of the disease, that is coronary artery abnormalities (CAAs), which include dilatations and aneurysms. Close monitoring of CAA is important, as ischaemic symptoms or myocardial infarction (MI) due to thrombosis or stenosis can occur. In Japan between 2017 and 2018, coronary artery dilatation, aneurysm, and giant aneurysm (lumen size ≥ 8 mm) within 30 days after KD onset were reported to occur in 7.64%, 0.95%, and 0.11% of patients, respectively (Ae 2020). Kawasaki disease is believed to be a leading cause of acquired heart disease in children from high‐income countries, with male patients or those resistant to initial intravenous immunoglobulin (IVIG) treatment at increased risk of CAAs (Newburger 2004; Uehara 2003).

The subacute phase is often asymptomatic, lasting approximately four weeks. During this time there may be peeling of the skin of the hands and feet, joint pain, and abnormal clinical findings such as thrombocytosis (increase in the number of platelets) or joint pain. This is also when the patient is at greatest risk of developing a coronary artery aneurysm. The convalescent phase is typically asymptomatic, four to eight weeks after onset.

Echocardiography is the standard imaging technique used to evaluate coronary abnormalities, with coronary arteries classified according to size. In children less than five years old, a coronary artery lumen diameter of 3 mm or more is abnormal, whilst in children five years or older, 4 mm or more is considered to be abnormal (JMHW 1984). Coronary artery lesions (CAL) are classified using Z scores (the coronary artery dimensions adjusted for body surface area, as dimensions will change with the size of the child) (Dallaire 2010; JMHW 1984; Kobayashi 2016; Olivieri 2008).

The prognosis for children with KD is highly dependent on the severity of coronary artery involvement. The fatality rate in the USA and Japan is reported as less than 0.2%, with MI from coronary occlusion being the main cause of death (Hayasaka 2003).

### Description of the intervention

It is thought that KD may be caused by activation of the immune system after infection with an unknown agent, such as a virus, in a genetically susceptible child. This results in an inflammatory cascade where both the innate and adaptive arms of the immune system are activated (Franco 2010; Gedalia 2007; Rowley 1997). However, no infectious cause has yet been identified. A genetic role is indicated by the ethnic relationships and by increased incidence in children whose parents or siblings have also had KD (Uehara 2003; Yashiro 2004), as well as by polymorphisms identified in different genes and gene regions by family linkage and genome studies (Onouchi 2008; Onouchi 2010; Onouchi 2012).

The primary treatments for KD are IVIG and acetylsalicylic acid (ASA) (Newburger 2004; Rife 2020). Standard regimen of the primary treatment consists of a single infusion of high‐dose IVIG (2 g/kg) together with ASA (Newburger 1991). IVIG is most effective when administered within 10 days of the onset of fever, and has been reported to reduce the risk of coronary artery aneurysm formation from 20% to 25%, to 3% to 5% (Newburger 1986). As many as 20% of children are considered to be IVIG resistant (or refractory), as they develop recurrent or persistent fevers after primary treatment (Ashouri 2008; Mori 2004; Newburger 2004). Adjunctive therapy may benefit those patients who are at higher risk of coronary artery aneurysms. Adjuvant treatments may include the use of corticosteroids and tumour necrosis factor‐alpha (TNF‐alpha) blockers such as etanercept and infliximab. Corticosteroids have been shown to reduce the incidence of CALs in KD and decrease fever, duration of hospitalisation, and time to normalisation of C‐reactive protein (CRP) levels (Green 2022). Recent American Heart Association (AHA) guidelines state that giving high‐risk or IVIG‐resistant patients a longer course of corticosteroids should be considered as primary adjunctive therapy (McCrindle 2017). Compared with no treatment or additional treatment with IVIG, TNF‐alpha blockers may have beneficial effects on treatment resistance and the unwanted 'infusion reaction' after treatment initiation for KD (Yamaji 2019).

Other agents include interleukin‐1 (IL‐1) receptor inhibitors (Kone‐Paut 2018), calcineurin inhibition therapy (ciclosporin) (Hamada 2019), cyclophosphamide, methotrexate (Lee 2008), rituximab (Sauvaget 2012), and plasma exchange (Hokosaki 2012), but their use is not widespread due to a lack of evidence (Rife 2020). Statins are also undergoing investigation due to their effects on inflammation, platelet aggregation, coagulation, and endothelial function (Tremoulet 2019).

### How the intervention might work

Exactly how IVIG works as a treatment of KD is unknown, but it has a general anti‐inflammatory effect, probably by modulating cytokine and antibody production and by increasing regulatory T‐cell activity (Burns 2015). Acetylsalicylic acid has anti‐inflammatory activity in high dose or antiplatelet activity in low dose, but it does not appear to prevent the development of coronary abnormalities (Baumer 2006). The remaining adjunctive and additional therapeutic agents also act by suppressing the widespread immune response characterised in KD with the aim of minimising symptoms and preventing cardiac abnormalities (Zhang 2020).

### Why it is important to do this review

Kawasaki disease is an important cause of acquired heart disease in children in high‐income countries, with the majority of deaths resulting from damage to the coronary arteries. In addition, unexpected death from MI can happen many years later, with incidences of non‐fatal and fatal MI in young adults sometimes thought to result from 'missed' KD in childhood (Burns 1996; Daniels 2012). The primary aim of an accurate diagnosis is to help prevent these complications with quick and effective treatment, and IVIG is widely used for this purpose. In 2020, with the SARS‐CoV‐2 pandemic, increased numbers of KD symptoms have been reported (Verdoni 2020). This review will replace an earlier Cochrane Review on the same topic (Oates‐Whitehead 2003). A new review is planned due to significant changes in Cochrane methodology since the previous review was published. We also aim to include all currently available evidence for IVIG for the treatment of KD in children to aid decision‐making for healthcare providers internationally.

## Objectives

To evaluate the efficacy and safety of intravenous immunoglobulin (IVIG) in treating and preventing cardiac consequences of Kawasaki disease.

## Methods

### Criteria for considering studies for this review

#### Types of studies

We included all randomised controlled trials (RCTs) investigating the use of IVIG for the treatment of KD. We planned to include studies involving treatment for initial or refractory KD, or both. We excluded studies that did not investigate any of our outcomes of interest.

#### Types of participants

We included studies involving participants diagnosed with KD using Japanese or AHA guidelines (see Table 6) (Ayusawa 2005; McCrindle 2017).

#### Types of interventions

We included studies using IVIG to treat participants with KD. We included all doses and types of IVIG. We included studies with the following comparisons.

- IVIG versus placebo or no treatment.
- IVIG versus ASA.
- IVIG versus TNF‐alpha blockers.
- IVIG versus corticosteroids.
- IVIG versus IVIG (i.e. dose versus dose).
- IVIG versus any combination of the above providing IVIG was the only difference between the groups, and any treatment effect was not confounded with another co‐treatment.

We excluded studies comparing infusion speed when the same dose was used. We excluded studies that compared one type of IVIG versus another (i.e. different manufacturer or separated/sulphonated).

#### Types of outcome measures

We recorded the time points of outcomes reported by the included studies. We were interested in the acute phase (up to two weeks) and convalescent phase (four weeks or later after initial treatment).

##### Primary outcomes

- Incidence of CAAs diagnosed by echocardiography or coronary angiography defined by absolute diameter, JMHW 1984, or Z‐scores.
- Incidence of any adverse effects after treatment initiation.

##### Secondary outcomes

- Acute coronary syndromes, such as MI or coronary thrombus.
- Duration of fever (days).
- Need for additional treatment.
- Length of hospital stay (days).
- Mortality (all‐cause).

### Search methods for identification of studies

#### Electronic searches

The Cochrane Vascular Information Specialist conducted systematic searches of the following databases for RCTs and controlled clinical trials with no language, publication year, or publication status restrictions:

- Cochrane Vascular Specialised Register via the Cochrane Register of Studies (CRS‐Web; searched 26 April 2022);
- Cochrane Central Register of Controlled Trials (CENTRAL; Issue 3, 2022) via the Cochrane Register of Studies Online (CRSO);
- MEDLINE (Ovid MEDLINE Epub Ahead of Print, In‐Process & Other Non‐Indexed Citations, Ovid MEDLINE Daily and Ovid MEDLINE) 1946 to 26 April 2022;
- Embase Ovid (1974 to 26 April 2022);
- CINAHL EBSCO (Cumulative Index to Nursing and Allied Health Literature) (1982 to 26 April 2022).

We developed search strategies for other databases from the search strategy designed for MEDLINE. Where appropriate, these were combined with adaptations of the Highly Sensitive Search Strategy designed by the Cochrane for identifying RCTs and controlled clinical trials (as described in Chapter 4 of the *Cochrane Handbook for Systematic Reviews of Interventions*, Lefebvre 2022). Search strategies for the major databases are provided in Appendix 1.

We searched the following trials registries:

- World Health Organization International Clinical Trials Registry Platform (who.int/trialsearch);
- ClinicalTrials.gov (clinicaltrials.gov).

The most recent searches were carried out on 26 April 2022.

#### Searching other resources

We checked the reference lists of included studies and relevant review articles to identify other ongoing or published studies. We contacted relevant manufacturers for trial information (Baxter Healthcare, Teijin Ltd, Mitsubishi Pharma Corp, Japan Blood Products Organization, Nihon Pharmaceutical Co Ltd, N‐Bayer Yakuhin Ltd, KM Biologics, and Takeda Pharmaceuticals).

### Data collection and analysis

#### Selection of studies

We used Covidence software to screen all reports identified by the Information Specialist (Covidence). One of two review authors assessed reports by title or abstract (CB, SK), with any articles clearly not meeting the inclusion criteria (e.g. non‐RCTs) considered as 'not relevant'. We obtained the full‐text reports of all studies deemed potentially relevant. Two of three review authors (CB, SK, MS) independently assessed the full‐text reports for inclusion in the review. Any disagreements were resolved by discussion. We collated multiple reports of the same study so that each study, rather than each report, was the unit of interest in the review. We illustrated the study selection process in a PRISMA diagram (Liberati 2009). We listed all articles excluded after full‐text assessment in the Characteristics of excluded studies tables and provided the reasons for their exclusion.

#### Data extraction and management

We used a data collection form based on the form provided by Cochrane Vascular to record study characteristics and outcome data. One of three review authors (CB, SK, MS) extracted the following study characteristics from the included studies.

- Methods (study design, number of participants, exclusions postrandomisation, losses to follow‐up, intention‐to‐treat analysis, duration of study).
- Participants (country, setting, age, sex, inclusion and exclusion criteria).
- Interventions (intervention, comparison, concomitant medications).
- Outcomes (primary and secondary outcomes specified and collected, and time points reported).
- Funding source and declaration of interest of the study authors.

One of three review authors (CB, SK, MS) independently extracted outcome data from the included studies, which a second review author (CB, SK, or MS) checked. When multiple trial arms were reported in a single trial, we included only the relevant arms. Any disagreements were resolved by consensus or by involving a third review author (TK, SI). One review author (CB) transferred data into Review Manager Web (RevMan Web 2022). We double‐checked that data were entered correctly. In the case of unclear or incomplete information or data, we contacted the study authors to request clarification. Additional information was provided for Matsushima 1985.

#### Assessment of risk of bias in included studies

Two of three review authors (CB, SK, MS) independently assessed the risk of bias of each included study using the criteria outlined in the *Cochrane Handbook for Systematic Reviews of Interventions* (Higgins 2011). Any disagreements were resolved by discussion or by involving another review author (TK). We assessed risk of bias according to the following domains.

- Random sequence generation
- Allocation concealment
- Blinding of participants and personnel
- Blinding of outcome assessment
- Incomplete outcome data
- Selective outcome reporting
- Other bias

We graded each domain as low, high, or unclear risk of bias, and provided a statement to justify our judgement in the risk of bias table. We summarised the risk of bias judgements across different studies for each of the domains listed. Where necessary, we considered blinding separately for cardiac outcomes and non‐cardiac outcomes.

#### Measures of treatment effect

We analysed dichotomous data as odds ratios (OR) with 95% confidence intervals (CI), and continuous data as mean difference (MD) with 95% CI. We planned to use standardised mean difference (SMD) if outcomes were reported using different measurement scales, but this was not necessary.

#### Unit of analysis issues

The unit of analysis was each individual participant. Some papers reported multiple studies. We entered the separate studies into the Characteristics of included studies tables by author, followed by date, followed by either A, B, or C (where A is the earliest of the trials documented) (i.e. Smith 1990A, Smith 1990B, Smith 1990C). Some studies reported three comparison arms. We were careful there were no unit of analysis issues with double‐counting of participants if included studies used multiple intervention arms. If two comparisons (e.g. drug A versus control and drug B versus control) were combined in the same meta‐analysis, we halved the control group to avoid double‐counting. We used intention‐to‐treat analysis when possible.

#### Dealing with missing data

We contacted study authors to obtain missing study characteristics or outcome data when necessary. When this was not possible, and the missing data were considered sufficient to introduce bias, we would explore the impact of including these studies by sensitivity analysis. We considered missing data sufficient to introduce bias if the missing data were imbalanced between study arms or were potentially a result of the intervention.

#### Assessment of heterogeneity

We assessed heterogeneity visually by inspecting forest plots. We also used the Chi^2^ and I^2^ statistics and Tau^2^ in accordance with the *Cochrane Handbook for Systematic Reviews of Interventions* (Higgins 2021). We interpreted the I^2^ value approximately as follows:

- 0% to 40%: might not be important;
- 30% to 60%: may represent moderate heterogeneity;
- 50% to 90%: may represent substantial heterogeneity;
- 75% to 100%: considerable heterogeneity.

When assessing the importance of the observed value of I^2^, we considered (i) the magnitude and direction of effects and (ii) the strength of evidence for heterogeneity (e.g. P value from the Chi^2^ test, or a CI for I^2^) in accordance with the *Cochrane Handbook for Systematic Reviews of Interventions* (Higgins 2021). If we detected heterogeneity, we explored the reasons for it through subgroup analysis.

#### Assessment of reporting biases

We planned that when more than 10 studies were pooled for a given outcome, we would create a funnel plot to explore possible small‐study and publication biases.

#### Data synthesis

We carried out data synthesis using Review Manager Web (RevMan Web 2022). We planned to use a fixed‐effect model when there were no concerns about heterogeneity. As there were differences between many of the studies (doses, infusion regimen, concomitant medications, etc.), we used a random‐effects model for all analyses, not just in the case of substantial heterogeneity (I^2^ > 50%). We only undertook meta‐analyses where this was meaningful, that is if the treatments, participants, and the underlying clinical question were similar enough for pooling to make sense. When meta‐analysis was not possible, we reported the results using a narrative synthesis.

#### Subgroup analysis and investigation of heterogeneity

We had planned to undertake subgroup analyses by initial or refractory (secondary) IVIG treatment and by single‐ or multiple‐dose regimens. Given the range of regimens involved in the included studies, it was clinically more appropriate to present initial versus secondary IVIG treatment, and single versus multiple doses as separate comparisons and subgroup by the total dose administered. We investigated any impact on the geographical distribution of participants by subgrouping by trial country setting when possible. When only limited data were available, we considered whether it was appropriate to conduct subgroup analysis, as in such cases results may reflect a lack of information rather than a true effect (Deeks 2021). We used the formal test for subgroup interactions in Review Manager Web (RevMan Web 2022).

We had planned to undertake subgroup analyses on the day of IVIG treatment, age, and weight/body mass index (BMI), but these analyses were precluded by insufficient information. We investigated any impact of study risk of bias using sensitivity analysis (see below).

#### Sensitivity analysis

We undertook sensitivity analyses to check if the results were robust by excluding studies at high risk of bias from the analysis. We considered studies to be at high risk of bias when assessed as being at high risk of selection bias (i.e. high risk for either random sequence generation or allocation sequence concealment), or at high risk of detection bias for cardiac outcomes. We also undertook sensitivity analysis when it was not clear what KD diagnostic criteria had been used. We further undertook sensitivity analysis when we identified substantial heterogeneity by removing studies from the analyses to assess the individual impact of studies on the results.

#### Summary of findings and assessment of the certainty of the evidence

We created a summary of findings table to present the evidence in the review for the following outcomes.

- Incidence of CAAs.
- Incidence of any adverse effects after treatment initiation.
- Acute coronary syndrome such as MI or coronary thrombus.
- Duration of fever.
- Need for additional treatment.
- Length of hospital stay.
- Mortality (all‐cause).

We included a table for the most clinically relevant comparisons. These were: primary treatment with IVIG compared to ASA (see Table 1); primary treatment with IVIG compared to different infusion regimens of IVIG (see Table 3); and primary treatment with IVIG compared to prednisolone (see Table 5). We used the five GRADE considerations (risk of bias, inconsistency, imprecision, indirectness, and publication bias) to assess the certainty of the evidence as it relates to the studies which contributed data to the meta‐analyses for each prespecified outcome (Atkins 2004). We used the methods described in the *Cochrane Handbook for Systematic Reviews of Interventions* (Higgins 2021; Schünemann 2021), employing GRADEpro GDT software (GRADEpro GDT). We explained all decisions to downgrade the certainty of evidence using footnotes and added comments to aid the reader's understanding of the review when needed.

## Results

### Description of studies

#### Results of the search

The searches identified a total of 2679 articles. An additional 23 records were identified from citation screening. After deduplication, we screened 1957 articles by title and abstract. We assessed 237 full‐text articles for eligibility. We included 31 studies (49 records). Several records reported on more than one study. We excluded 13 studies (16 records) with reasons provided. We identified two ongoing studies (three records) and 23 studies as awaiting classification. See Figure 1.

**1:**
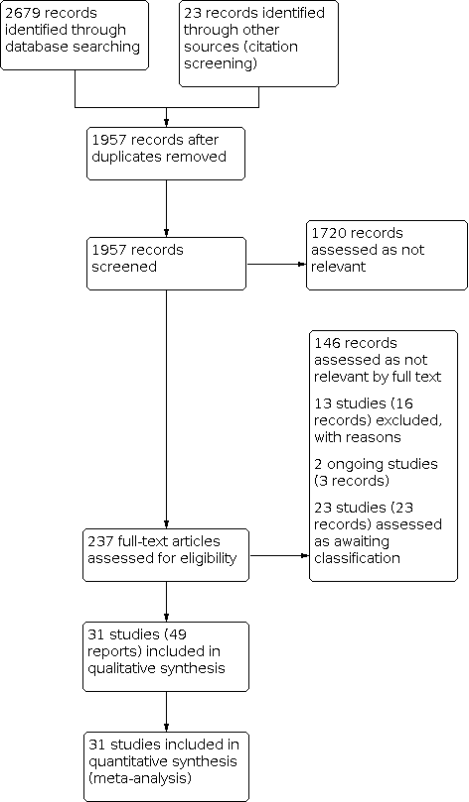
PRISMA flow diagram.

#### Included studies

See Characteristics of included studies tables.

We included 31 studies with a total of 4609 participants that investigated IVIG treatment for KD (Barron 1990; Burns 2008; Burns 2021; Furusho 1984; Furusho 1991A; Furusho 1991B; Harada 1989; Hashino 2001; He 2021; Matsushima 1985; Miura 2008; Mori 2017; Morikawa 1994; Nagashima 1987; Newburger 1986; Newburger 1991; Nishihara 1988; Nonaka 1995; Ogawa 1987; Ogino 1987; Okuni 1987A; Okuni 1987B; Onouchi 1988; Onouchi 1992; Qin 2006; Sakata 2007; Sato 1995; Wang 2020; Yabiku 1989; Youn 2016; Yuan 2000). The majority of studies (21) were carried out in Japan (Furusho 1984; Furusho 1991A; Furusho 1991B; Harada 1989; Hashino 2001; Matsushima 1985; Miura 2008; Mori 2017; Morikawa 1994; Nagashima 1987; Nishihara 1988; Nonaka 1995; Ogawa 1987; Ogino 1987; Okuni 1987A; Okuni 1987B; Onouchi 1988; Onouchi 1992; Sakata 2007; Sato 1995; Yabiku 1989). Five were carried out in the USA or the USA and Canada (Barron 1990; Burns 2008; Burns 2021; Newburger 1986; Newburger 1991), four in China (He 2021; Qin 2006; Wang 2020; Yuan 2000), and one in Korea (Youn 2016).

The size of the included studies varied, with the smallest having 17 participants, Hashino 2001, and the largest 549 participants, Newburger 1991. Nishihara 1988 did not report the numbers of male and female participants. As expected, clinically all studies reported a larger proportion of males overall, with one exception being Yabiku 1989, which reported equal numbers of males and females in the control group.

The age of participants ranged from two months, Hashino 2001, to 14 years, Qin 2006, with the majority of participants aged between 18 and 30 months (Barron 1990; Burns 2008; Furusho 1984; Furusho 1991A; Furusho 1991B; Harada 1989; He 2021; Matsushima 1985; Miura 2008; Mori 2017; Nagashima 1987; Newburger 1986; Newburger 1991; Nishihara 1988; Nonaka 1995; Ogawa 1987; Ogino 1987; Okuni 1987A; Okuni 1987B; Onouchi 1988; Onouchi 1992; Sakata 2007; Sato 1995; Wang 2020; Yabiku 1989). Four studies included some older participants (Burns 2021; Morikawa 1994; Youn 2016; Yuan 2000).

The majority of studies reported on the primary treatment of KD (Barron 1990; Furusho 1984; Furusho 1991A; Furusho 1991B; Harada 1989; He 2021; Matsushima 1985; Morikawa 1994; Nagashima 1987; Newburger 1986; Newburger 1991; Nishihara 1988; Nonaka 1995; Ogawa 1987; Ogino 1987; Okuni 1987A; Okuni 1987B; Onouchi 1988; Onouchi 1992; Qin 2006; Sato 1995; Yabiku 1989; Yuan 2000). One study reported on primary plus additional treatment (Sakata 2007). Six studies reported on secondary or refractory treatment for KD (Burns 2008; Burns 2021; Miura 2008; Mori 2017; Wang 2020; Youn 2016), and one study reported on tertiary treatment (Hashino 2001).

For those studies reporting on primary treatment, treatment was initiated within seven days of onset in 12 studies (Barron 1990; Furusho 1984; Furusho 1991A; Furusho 1991B; Harada 1989; Matsushima 1985; Nishihara 1988; Okuni 1987A; Okuni 1987B; Onouchi 1988; Onouchi 1992; Yabiku 1989), and 10 days in eight studies (He 2021; Morikawa 1994; Nagashima 1987; Newburger 1986; Newburger 1991; Nonaka 1995; Sakata 2007; Sato 1995). Initiation of treatment was not clear in Qin 2006 and Yuan 2000.

All studies investigating secondary treatment used IVIG as the initial treatment. Secondary treatment was initiated when fever did not decrease within 36 hours to seven days after initial treatment (Burns 2008; Burns 2021), or 24 to 48 hours after initial treatment (Miura 2008; Mori 2017; Wang 2020; Youn 2016).

Some studies reported three arms (Furusho 1991A; Furusho 1991B; He 2021; Nishihara 1988; Onouchi 1988; Onouchi 1992). When necessary, to be able to report these in analyses using Review Manager Web, we split one of the comparison arms between the other two arms to prevent double‐counting of participants.

We identified and included studies with the following comparisons:

- IVIG compared to ASA;
- IVIG compared to a different dose of IVIG;
- IVIG compared to infliximab;
- IVIG compared to methylprednisolone.

We did not identify any randomised studies comparing IVIG to placebo or no treatment.

The total dose and frequency of IVIG treatment varied between studies and ranged from 50 to 100 mg/kg/day to 2 g/kg/day as either a single dose or daily infusions for up to five days. Infusion times ranged from one to 24 hours. The majority of participants received ASA in addition to IVIG. One study also administered dipyridamole (Nonaka 1995). In Burns 2008, participants also received paracetamol and diphenhydramine; heparin was infused with methylprednisolone in Miura 2008; and Sakata 2007 administered flurbiprofen to participants with liver dysfunction.

The majority of studies compared initial IVIG to a different dose or regimen of initial IVIG, Barron 1990; Furusho 1991A; Harada 1989; He 2021; Morikawa 1994; Newburger 1991; Nishihara 1988; Onouchi 1992; Qin 2006; Sakata 2007; Sato 1995, or to ASA alone, Furusho 1984; Furusho 1991B; Matsushima 1985; Nagashima 1987; Newburger 1986; Ogawa 1987; Ogino 1987; Okuni 1987A; Okuni 1987B; Onouchi 1988; Yabiku 1989. Nonaka 1995 and Yuan 2000 compared IVIG to prednisolone. Studies investigating secondary treatment compared IVIG to infliximab, Burns 2008; Burns 2021; Mori 2017; Youn 2016, or methylprednisolone, Miura 2008; Wang 2020. Hashino 2001 investigated tertiary treatment and compared IVIG with methylprednisolone. For specific details on doses and regimens, see the Characteristics of included studies tables.

Coronary artery abnormalities were reported by all included studies, the majority of which described detection and classification of CAA with echocardiography (Barron 1990; Burns 2008; Burns 2021; Furusho 1984; Furusho 1991A; Furusho 1991B; Hashino 2001; He 2021; Miura 2008; Mori 2017; Morikawa 1994; Nagashima 1987; Newburger 1986; Newburger 1991; Nishihara 1988; Nonaka 1995; Okuni 1987A; Okuni 1987B; Onouchi 1988; Onouchi 1992; Sakata 2007; Sato 1995; Wang 2020; Youn 2016). Seven studies did not describe how they classified CAA (Harada 1989; Matsushima 1985; Ogawa 1987; Ogino 1987; Qin 2006; Yabiku 1989; Yuan 2000).

The remaining outcomes of interest were reported by only some studies:

- adverse effects (Barron 1990; Burns 2008; Burns 2021; Furusho 1984; Furusho 1991A; Harada 1989; He 2021; Matsushima 1985; Miura 2008; Mori 2017; Morikawa 1994; Nagashima 1987; Newburger 1986; Newburger 1991; Nonaka 1995; Ogawa 1987; Ogino 1987; Okuni 1987A; Okuni 1987B; Onouchi 1988; Onouchi 1992; Qin 2006; Sato 1995; Wang 2020; Yabiku 1989; Youn 2016);
- acute coronary syndromes (Matsushima 1985; Newburger 1986; Nonaka 1995);
- duration of fever (Barron 1990; Burns 2008; Burns 2021; Furusho 1984; Harada 1989; Hashino 2001; He 2021; Matsushima 1985; Miura 2008; Mori 2017; Morikawa 1994; Nagashima 1987; Newburger 1986; Newburger 1991; Nishihara 1988; Nonaka 1995; Ogawa 1987; Ogino 1987; Onouchi 1988; Onouchi 1992; Qin 2006; Sakata 2007; Sato 1995; Wang 2020; Yabiku 1989; Youn 2016; Yuan 2000);
- need for additional treatment (Burns 2008; Burns 2021; Furusho 1984; He 2021; Matsushima 1985; Mori 2017; Morikawa 1994; Newburger 1991; Onouchi 1988; Onouchi 1992; Sakata 2007; Wang 2020; Youn 2016);
- length of hospital stay (Barron 1990; Burns 2021; He 2021; Newburger 1991; Qin 2006; Sakata 2007; Sato 1995; Youn 2016);
- mortality (Burns 2021; Furusho 1984; Matsushima 1985; Miura 2008; Newburger 1991; Nonaka 1995).

Eleven studies reported public or government funding sources (Burns 2008; Burns 2021; Harada 1989; Hashino 2001; He 2021; Newburger 1986; Newburger 1991; Okuni 1987A; Okuni 1987B; Qin 2006; Wang 2020). Three studies received funding (or additional funding) from pharmaceutical companies that manufacture blood plasma products or immune‐regulating products (Barron 1990; Burns 2008; Mori 2017). Three studies acknowledged pharmaceutical companies for donating gammaglobulin (Furusho 1984; Ogawa 1987; Ogino 1987). The remaining studies did not report funding sources (Furusho 1991A; Furusho 1991B; Matsushima 1985; Miura 2008; Morikawa 1994; Nagashima 1987; Nishihara 1988; Nonaka 1995; Onouchi 1988; Onouchi 1992; Sakata 2007; Sato 1995; Yabiku 1989; Youn 2016; Yuan 2000). See Characteristics of included studies.

#### Excluded studies

We excluded a total of 13 studies based on full‐text assessment (ChiCTR2000035163; Hamada 2019; ISRCTN71987471; JPRN‐UMIN000014665; Lee 1996; Lin 2021; Muta 2002; Nanishi 2017; NCT02298062; Portman 2019; RAISE 2012; Sanati 2021; Seki 2021). The reasons for exclusion were as follows:

- IVIG was not the differentiating intervention (ChiCTR2000035163; Hamada 2019; Lin 2021; Nanishi 2017; RAISE 2012; Sanati 2021);
- study compared injection or infusion speed of IVIG (JPRN‐UMIN000014665; Seki 2021);
- study only reported surrogate outcomes (lymphocyte phenotypes), and so did not meet our inclusion criteria (Lee 1996);
- study compared different types of IVIG, and dosage was determined by KD severity (Muta 2002);
- study randomised to treatment (not IVIG) after initial IVIG treatment (ISRCTN71987471; Portman 2019);
- study not completed due to limited participants and COVID‐19 (NCT02298062).

For details of the excluded studies, see Characteristics of excluded studies tables.

#### Ongoing studies

We identified two ongoing studies (ChiCTR1900027954; EUCTR2020‐003194‐22‐FR). For details, see Characteristics of ongoing studies tables.

#### Studies awaiting classification

We assessed 23 reports as awaiting classification (Chang 2007; Chang 2008; Chen 2006; Fu 2010; Ho 2003; Ho 2004; Juan 2003; Juan 2005; Juan 2006; Kuo 2009; Li 2008; Li 2009; Liao 2007; Liu 2000; Liu 2004; Liu 2009; Lu 2003; Peng 2001; Teng 2005; Yao 2009; Yen 2007; Yuan 2009; Yueh 2006), which were identified by citation screening from published reviews and not by our database searches. We are currently unable to verify if they meet our inclusion criteria.

### Risk of bias in included studies

We assessed the risk of bias of each included study based on the seven domains of the Cochrane risk of bias tool (RoB 1). An overall summary of bias present within each of the included studies is presented in Figure 2 and Figure 3. No studies were at low risk of bias in all domains. Of the 31 included studies, 24 studies had at least one domain at high risk of bias. No study was at high risk of bias in all domains. All studies were at unclear risk of bias in at least one domain.

**2:**
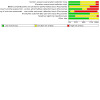
Risk of bias graph: review authors' judgements about each risk of bias item presented as percentages across all included studies.

**3:**
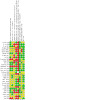
Risk of bias summary: review authors' judgements about each risk of bias item for each included study.

#### Allocation

We selected only RCTs for this review. However, we judged only eight studies to be at low risk of bias for both random sequence generation and allocation concealment domains (Burns 2021; Harada 1989; Mori 2017; Morikawa 1994; Newburger 1986; Newburger 1991; Sakata 2007; Wang 2020). We judged Yuan 2000 to be at high risk for both random sequence generation and allocation concealment domains, as hospital numbers were used to assign treatment. We judged Matsushima 1985 and Nagashima 1987 to be at high risk for both random sequence generation and allocation concealment domains, as odd and even numbers were used to assign treatment. Four studies provided sufficient details to be assessed as at low risk for random sequence generation, but insufficient information to be assessed as low or high risk for allocation concealment, and were therefore judged as unclear (Barron 1990; Burns 2008; He 2021; Sato 1995). Four studies stated that randomisation was arranged centrally, but did not provide details, so were assessed as unclear for random sequence generation and low for allocation concealment (Ogawa 1987; Ogino 1987; Onouchi 1992; Yabiku 1989). Twelve studies did not describe the method of randomisation and allocation at all or did not describe their methods in sufficient detail to permit an assessment, so were assessed as at unclear risk of bias for both domains (Furusho 1984; Furusho 1991A; Furusho 1991B; Hashino 2001; Miura 2008; Nishihara 1988; Nonaka 1995; Okuni 1987A; Okuni 1987B; Onouchi 1988; Qin 2006; Youn 2016).

#### Blinding

##### Performance bias

We assessed one study to be at low risk of performance bias, as blinding of clinicians was described (Sakata 2007). We judged nine studies to be at high risk of performance bias, as it was clearly stated that no blinding of participants and personnel had been undertaken (Burns 2008; Burns 2021; Hashino 2001; He 2021; Miura 2008; Mori 2017; Nagashima 1987; Sato 1995; Wang 2020). We assessed the remaining 21 studies as at unclear risk because they did not provide sufficient details to be assessed as at either low or high risk of performance bias. We acknowledge that given the different infusion times between some of the treatments, blinding of participants and personnel would have been difficult.

##### Detection bias

We assessed each study for detection bias for both cardiac abnormality outcomes and non‐cardiac abnormality outcomes. This was because blinding of cardiac outcome assessment was possible even in situations where blinding of personnel or participants was not.

Fourteen studies provided a clear description of blinding for evaluating cardiac abnormalities and so were judged to be at low risk of detection bias (Barron 1990; Harada 1989; He 2021; Mori 2017; Morikawa 1994; Newburger 1986; Newburger 1991; Nishihara 1988; Okuni 1987A; Okuni 1987B; Onouchi 1988; Onouchi 1992; Sakata 2007; Sato 1995). This was generally achieved by echocardiogram (ECG) recordings being assessed by clinicians who were not aware of the participant's identity. We judged 12 studies to be at high risk of detection bias for cardiac outcomes because there were no blinding measures, and the outcome could have been influenced by a lack of blinding (Burns 2008; Furusho 1984; Furusho 1991A; Furusho 1991B; Matsushima 1985; Miura 2008; Nagashima 1987; Nonaka 1995; Qin 2006; Wang 2020; Youn 2016; Yuan 2000). When blinding of outcome assessors was insufficiently described, we judged the risk of detection bias as unclear (Burns 2021; Hashino 2001; Ogawa 1987; Ogino 1987; Yabiku 1989).

For non‐cardiac outcomes, Onouchi 1988 and Onouchi 1992 reported that independent assessors were used for non‐cardiac outcomes, therefore we judged these studies to be at low risk of detection bias. We judged 24 studies to be at high risk of bias, as a lack of blinding was likely to influence outcomes (Barron 1990; Burns 2008; Burns 2021; Furusho 1984; Furusho 1991A; Furusho 1991B; Harada 1989; Hashino 2001; He 2021; Matsushima 1985; Miura 2008; Mori 2017; Morikawa 1994; Nagashima 1987; Newburger 1986; Newburger 1991; Nonaka 1995; Okuni 1987A; Okuni 1987B; Qin 2006; Sato 1995; Wang 2020; Youn 2016; Yuan 2000). We judged four studies to be at unclear risk of detection bias for non‐cardiac outcomes because insufficient details on how these outcomes were assessed were provided, or none of the outcomes measured was likely to be impacted by a lack of blinding (Ogawa 1987; Ogino 1987; Sakata 2007; Yabiku 1989). One study did not report on non‐cardiac outcomes, preventing a judgement of detection bias (Nishihara 1988).

#### Incomplete outcome data

We assessed no studies as being at high risk of attrition bias. The majority of included studies were at low risk of attrition bias, as data were all reported or accounted for (Barron 1990; Burns 2008; Burns 2021; Furusho 1984; Harada 1989; Hashino 2001; He 2021; Matsushima 1985; Miura 2008; Morikawa 1994; Nagashima 1987; Newburger 1986; Newburger 1991; Nishihara 1988; Ogino 1987; Okuni 1987A; Okuni 1987B; Onouchi 1992; Sakata 2007; Sato 1995; Wang 2020; Youn 2016). Nine studies were at unclear risk of attrition bias because missing data were sufficient to potentially impact the results (Mori 2017; Ogawa 1987; Yabiku 1989), or the reasons for missing data were unclear (Furusho 1991A; Furusho 1991B; Nonaka 1995; Onouchi 1988; Qin 2006; Yuan 2000).

#### Selective reporting

We assessed Furusho 1991A and Furusho 1991B as being at high risk of reporting bias, as no description of outcomes or measurements was provided in a methods section. Seven studies were at low risk of reporting bias, as all expected or planned outcomes per study protocols or trial databases were reported in the results (Burns 2008; Burns 2021; Hashino 2001; He 2021; Mori 2017; Newburger 1991; Wang 2020). We assessed the majority of included studies (22) as at unclear risk of reporting bias, as no protocol was available, or it was not clear what the planned outcomes were (Barron 1990; Furusho 1984; Harada 1989; Matsushima 1985; Miura 2008; Morikawa 1994; Nagashima 1987; Newburger 1986; Nishihara 1988; Nonaka 1995; Ogawa 1987; Ogino 1987; Okuni 1987A; Okuni 1987B; Onouchi 1988; Onouchi 1992; Qin 2006; Sakata 2007; Sato 1995; Yabiku 1989; Youn 2016; Yuan 2000).

#### Other potential sources of bias

We had no concerns about other potential biases in 21 studies (Burns 2008; Burns 2021; Furusho 1984; Furusho 1991A; Furusho 1991B; Harada 1989; Hashino 2001; He 2021; Matsushima 1985; Morikawa 1994; Nagashima 1987; Newburger 1986; Newburger 1991; Ogawa 1987; Ogino 1987; Okuni 1987A; Okuni 1987B; Onouchi 1992; Qin 2006; Sakata 2007; Yuan 2000). No studies were at high risk of other bias. We assessed the remaining studies as at unclear risk of other bias. Reasons for this included an imbalance in gender or ages between groups, and we were not sure if this could have affected the results (Barron 1990; Nonaka 1995; Onouchi 1988; Sato 1995; Yabiku 1989; Youn 2016); study was halted prematurely (Miura 2008); inconsistencies in reporting (Wang 2020); treatments were switched at physician's discretion because of worsening KD in a substantial proportion of participants in both groups (Mori 2017); and results were published as a letter or abstract and likely not peer reviewed (Nishihara 1988).

### Effects of interventions

See: Table 1; Table 3; Table 5

We have presented the results of the studies by each comparison and outcome of interest as pre‐planned. For the comparison of IVIG versus IVIG, the studies were heterogeneous in the dose of IVIG used and their infusion regimens. The total dose and frequency of IVIG treatment ranged from 50 to 100 mg/kg/day to 2 g/kg/day as either a single dose or daily infusions for up to five days. In order to present this information as clearly and as usefully as possible, we have indicated the dose and if this was over multiple days (e.g. 200 mg/kg/day for five days), as this is the most clinically familiar way. In the analysis tables, we presented the doses as the total IVIG received and indicated if this was on single or multiple days (e.g. 1000 mg/kg in five days). This was to facilitate a clearer comparison of regimens. We used a random‐effects model for all analyses due to the differences between studies. For specific details on interventions and concomitant medications, please see Characteristics of included studies.

#### Primary treatment ‐ intravenous immunoglobulin (IVIG) versus acetylsalicylic acid (ASA)

See Table 1.

Eleven studies involving 1396 participants compared IVIG treatment with ASA (Furusho 1984; Furusho 1991B; Matsushima 1985; Nagashima 1987; Newburger 1986; Ogawa 1987; Ogino 1987; Okuni 1987A; Okuni 1987B; Onouchi 1988; Yabiku 1989). All studies compared IVIG plus ASA versus ASA. One of two IVIG study arms in Furusho 1991B was not given ASA. Most studies administered ASA initially at 30 mg/kg/day, except for Newburger 1986, who used 100 mg/kg/day every 6 hours to day 14 of illness, and Okuni 1987A, Okuni 1987B, and Onouchi 1988, who used 50 mg/kg/day in both groups. Studies differed in total IVIG dose administered, number of doses, and time points measured.

##### Incidence of coronary artery abnormalities (CAAs)

All 11 studies reported on the incidence of CAA (Furusho 1984; Furusho 1991B; Matsushima 1985; Nagashima 1987; Newburger 1986; Ogawa 1987; Ogino 1987; Okuni 1987A; Okuni 1987B; Onouchi 1988; Yabiku 1989). Where possible, we have presented numbers of CAA excluding any CAA present at enrolment.

Overall, there were fewer CAA detected up to 30 days in the IVIG treatment group compared to the ASA group (odds ratio (OR) 0.60, 95% confidence interval (CI) 0.41 to 0.87; P = 0.008; 11 studies, 1437 participants; moderate‐certainty evidence). See Analysis 1.1.

Seven studies reported CAA at 60 days or 1 year (Furusho 1984; Newburger 1986; Ogawa 1987; Ogino 1987; Okuni 1987A; Okuni 1987B; Yabiku 1989). There was no clear difference detected at 60 days and over in the IVIG treatment group compared to the ASA group (OR 0.79, 95% CI 0.45 to 1.38; P = 0.41; 7 studies, 679 participants). See Analysis 1.2.

Furusho 1984 reported 6/40 (15%) CAA in the IVIG group at 30 days, compared to 19/45 (42%) in the ASA group. No new lesions were detected between day 30 and 60; 14 CAA persisted in the ASA group compared to three CAA in the IVIG group. At day 60, 1/6 CAA were detected in the IVIG group compared to 11/19 CAA in the ASA group.

Furusho 1991B investigated three groups: ASA, ASA + IVIG 200 mg/kg/day, and IVIG 200 mg/kg/day for five days. Before day 30, CAA was reported in 9/49, 10/53, and 19/49 participants respectively. At day 30, CAA was detected in 5/49, 4/51, and 9/47 participants respectively. We halved the ASA group data between subgroup analysis to prevent double‐counting.

Matsushima 1985 compared IVIG 400 mg/kg over 5 days with ASA. This study reported CAA incidence (dilations and aneurysms) at 1, 2, 3, and 4 weeks: 0/17, 3/17, 1/17, and 1/17 versus 0/17, 6/17, 6/17, and 3/17 in the IVIG and ASA groups, respectively.

Nagashima 1987 compared IVIG 400 mg/kg over three days with ASA. This study reported the number of CAA (dilations and aneurysms) at 1, 2, 3, and 4 weeks, and up to day 30, where 11/69 CAA were detected in the IVIG group compared to 25/67 in the ASA group.

Newburger 1986 compared IVIG 400 mg/kg over four days with ASA. They reported the number of CAA at enrolment, 2 and 7 weeks, and 1‐year follow‐up. At enrolment, 2/84 participants in the IVIG group and 4/84 in the ASA group had CAA. At 2 and 7 weeks, excluding those at enrolment, there were 5/74 versus 15/75 and 2/77 versus 11/75 CAA in the IVIG and ASA groups, respectively. At 2 and 7 weeks and 1 year, including those at enrolment, there were 6/75 versus 18/78, 3/79 versus 14/79, and 2/7 versus 9/17 CAA in the IVIG and ASA groups, respectively.

Ogawa 1987 compared IVIG (400 mg/kg/day for 3 consecutive days) with ASA (30 mg/kg/day) versus ASA (30 mg/kg/day). The reported number of CAA at enrolment (1 to 10 days), under 11 to 25 days, 30 days, and 60 days were: 5/63, 11/63, 6/63, and 3/63 in the IVIG with ASA group versus 2/54, 17/54, 9/54, and 7/54 in the ASA group.

Ogino 1987 compared IVIG (200 mg/kg/day for 3 consecutive days) with ASA (30 mg/kg/day) versus ASA (30 mg/kg/day). The reported number of CAA at enrolment (1 to 10 days), under 11 to 25 days, 30 days, 60 days, and 1 year were: 3/50, 15/50, 13/50, 9/50, and 4/50 in the IVIG with ASA group versus 5/42, 13/42, 10/42, 6/42, and 3/42 in the ASA group.

Okuni 1987A compared single 100 mg/kg of IVIG (either pepsin treated or intact) versus ASA. We combined the two types of IVIG used. They reported CAA at enrolment, under 30 days, 30 days, 60 days, 6 months, and 1 year: 13/139, 55/136, 31/139, 24/139, 15/139, and 9/138 in the IVIG group versus 7/75, 29/75, 15/74, 11/74, 8/74, and 3/73 in the ASA group. The study authors reported no difference between groups if CAA numbers at enrolment were excluded, but we do not have those data.

Okuni 1987B compared 100 mg/kg of IVIG for five days (either pepsin treated or intact) versus ASA. We combined the two types of IVIG used. They reported CAA at enrolment, under 30 days, 30 days, 60 days, 6 months, and 1 year: 17/196, 78/192, 36/188, and 21/186 in the IVIG group versus 11/99, 50/97, 30/95, and 20/90 in the ASA group. They also reported the data excluding those present at enrolment for under 30 days, 30 days, 60 days, 6 months, and 1 year: 61/175, 27/171, and 15/169 in the IVIG group versus 39/86, 21/85, and 13/81 in the ASA group.

Onouchi 1988 compared three arms: 200 mg/kg IVIG for 3 days, 400 mg/kg IVIG for 3 days, and ASA. They reported CAA detected before treatment, and at days 15, 30, and 60: IVIG 200 mg/kg: 6/38, 15/38, 10/38, and 7/38 versus IVIG 400 mg/kg: 5/42, 16/42, 10/42, and 6/42 versus ASA: 7/46, 19/46, 14/46, and 6/46. We included the day 15 data in the analysis. We split the ASA group between the analysis to prevent double‐counting.

Yabiku 1989 compared a single dose of IVIG 1 g/kg with ASA versus ASA. They reported the number of CAA at enrolment (1 to 10 days), under 11 to 25 days, 30 days, and 60 days: 4/48, 13/48, 8/48, and 3/48 in the IVIG with ASA group versus 4/36, 12/36, 6/36, and 3/36 in the ASA group, respectively.

##### Incidence of any adverse effects after treatment initiation

See Analysis 1.3 and Table 2.

Ten studies reported a wide range of adverse effects, including: liver disorder, chills and fever, pericardial effusion, urticaria, mild congestive heart failure, shaking/itching, sepsis, lymphadenopathy, splenomegaly, neutropenia, and nasal haemorrhage (Furusho 1984; Matsushima 1985; Nagashima 1987; Newburger 1986; Ogawa 1987; Ogino 1987; Okuni 1987A; Okuni 1987B; Onouchi 1988; Yabiku 1989). No serious adverse events were reported. Furusho 1991B did not report adverse effects.

We combined the data in a meta‐analysis and described specific adverse effects by study in Table 2. Overall, there was no clear difference in the number of adverse effects between the IVIG and ASA groups (OR 0.57, 95% CI 0.17 to 1.89; P = 0.35; 10 studies, 1376 participants; very low‐certainty evidence). See Analysis 1.3. We detected substantial heterogeneity (I^2^ = 73%). This may have been due to the different adverse effects reported by each study. The test for subgroup differences did not detect a difference in adverse effects between the different total IVIG doses used (P = 0.10).

##### Acute coronary syndromes, such as myocardial infarction (MI) or coronary thrombus

Only Newburger 1986 reported the incidence of acute coronary syndromes. At 30 months, no adverse sequelae were reported in the 7 participants in the IVIG group followed up to this point. Two of 17 participants in the ASA group had a coronary artery thrombus. Matsushima 1985 provided additional information upon request and confirmed that no acute coronary events had occurred. No other studies reported this outcome. We assessed this evidence to be of low certainty.

##### Duration of fever (days)

Eight studies reported duration of fever (Furusho 1984; Matsushima 1985; Nagashima 1987; Newburger 1986; Ogawa 1987; Ogino 1987; Onouchi 1988; Yabiku 1989). Four studies reported duration from onset of disease (Furusho 1984; Ogawa 1987; Ogino 1987; Yabiku 1989). Three studies reported duration both from disease onset and onset of treatment (Matsushima 1985; Nagashima 1987; Onouchi 1988). Newburger 1986 reported the average fall in temperature and maximum temperature in hospitalised children.

We combined the data from studies reporting duration of fever from treatment onset. Overall, the duration of fever in the IVIG group was shorter than in the ASA group (mean difference (MD) −4.00 days, 95% CI −5.06 to −2.93; P < 0.001; 3 studies, 307 participants; moderate‐certainty evidence). See Analysis 1.4.

We also combined the data from studies reporting duration from disease onset. Overall, the duration of fever in the IVIG group was shorter than in the ASA group (MD −1.60 days, 95% CI −2.69 to −0.52; P = 0.004; 7 studies, 693 participants). See Analysis 1.5.

Newburger 1986 also reported data from centres where participants were hospitalised for at least two days to prevent bias from home reporting. Between day 1 and 2, the average temperature (± standard error, SE) of the IVIG group fell by 1.3 ± 0.16 ˚C compared to 0.42 ± 0.11 ˚C in the ASA group. At day 2, the number of participants with a maximum temperature ≥ 38 ˚C was 16/35 in IVIG group compared to 30/35 in the ASA group.

##### Need for additional treatment

Details on the need for additional treatment were limited, with three studies reporting this outcome. Furusho 1984 reported that one IVIG participant was retreated with IVIG and one ASA participant was treated with IVIG because of worsening symptoms. Matsushima 1985 reported that no participants needed retreatment. Onouchi 1988 reported that three ASA participants were retreated, but did not provide details about the medication given. Overall, there was no clear difference in need for additional retreatment between IVIG and ASA groups (OR 0.27, 95% CI 0.05 to 1.57; P = 0.15; 3 studies, 272 participants; low‐certainty evidence). See Analysis 1.6.

Nagashima 1987 mentions that 1/69 participants in the IVIG group received additional IVIG, but it is unclear if this was the only participant to do so. The need for additional treatment was not clearly reported in the remaining studies in this comparison, and no further information was available upon contacting study authors (Furusho 1991B; Newburger 1986; Ogawa 1987; Ogino 1987; Okuni 1987A; Okuni 1987B; Yabiku 1989).

##### Length of hospital stay (days)

None of the studies comparing IVIG with ASA reported on length of hospital stay (Furusho 1984; Furusho 1991B; Matsushima 1985; Nagashima 1987; Newburger 1986; Ogawa 1987; Ogino 1987; Okuni 1987A; Okuni 1987B; Onouchi 1988; Yabiku 1989).

##### Mortality (all‐cause)

Two studies reported details on mortality incidence, with no deaths occurring in either study (Furusho 1984; Matsushima 1985). The remaining studies did not mention mortality, and although it is likely this means there were no deaths, we cannot be sure about this (Furusho 1991B; Nagashima 1987; Newburger 1986; Ogawa 1987; Ogino 1987; Okuni 1987A; Okuni 1987B; Onouchi 1988; Yabiku 1989). We assessed this evidence to be of low certainty.

#### Primary treatment ‐ IVIG versus IVIG

See Table 3.

Twelve studies compared IVIG with a different dose of IVIG or single versus multiple infusions, or multiple versus multiple infusions (Barron 1990; Furusho 1991A; Harada 1989; He 2021; Morikawa 1994; Newburger 1991; Nishihara 1988; Onouchi 1988; Onouchi 1992; Qin 2006; Sakata 2007; Sato 1995).

Eight studies with a total of 1824 participants compared a high‐dose regimen versus a medium‐ or low‐dose regimen (Furusho 1991A; Harada 1989; He 2021; Morikawa 1994; Nishihara 1988; Onouchi 1992; Qin 2006; Sakata 2007). Sakata 2007 administered additional IVIG if needed after the first dose. We included data from the first infusion only in this analysis. We considered a high dose to be more than 1900 mg/kg total IVIG; medium to be 1900 mg/kg to 900 mg/kg total IVIG; and low to be less than 900 mg/kg total IVIG. These dose categories are the same as the nationwide survey in Japan (Ae 2020; Ae 2021). Some studies reported on three groups (Furusho 1991A; He 2021; Nishihara 1988; Onouchi 1992). Where appropriate, we split the data between subgroups to prevent double‐counting.

All eight studies infused a total of 2 g/kg using either a single infusion, He 2021; Qin 2006; Sakata 2007, or multiple infusions over two days, He 2021, or five days, Furusho 1991A; Harada 1989; Morikawa 1994; Nishihara 1988; Onouchi 1992. The medium and low doses ranged from 1000 mg/kg to 500 mg/kg total IVIG by single, He 2021; Qin 2006; Sakata 2007, or multiple infusions over five days, Furusho 1991A; Harada 1989; Morikawa 1994; Nishihara 1988; Onouchi 1992.

##### Incidence of CAAs

Meta‐analysis showed that fewer CAA were reported in the higher infusion regimens compared to the medium or lower regimens (OR 0.60, 95% CI 0.40 to 0.89; P = 0.01; 8 studies, 1824 participants; moderate‐certainty evidence). See Analysis 2.1. No differences were detected between subgroups (test for subgroup differences P = 0.07).

Three studies compared higher‐dose single‐infusion regimens with medium‐dose single infusion (He 2021; Qin 2006; Sakata 2007). No clear difference in incidence of CAA was detected between these regimens (OR 0.88, 95% CI 0.59 to 1.32; P = 0.53; 3 studies, 621 participants). See Analysis 2.2.

Six studies investigated higher‐dose multiple‐infusion versus lower‐dose multiple‐infusion regimens (Furusho 1991A; Harada 1989; Morikawa 1994; Nishihara 1988; Onouchi 1988; Onouchi 1992). Higher multiple‐infusion doses were 2 g/kg, Furusho 1991A; Harada 1989; Morikawa 1994; Nishihara 1988; Onouchi 1992, or 1000 mg/kg total, Furusho 1991A; Nishihara 1988; Onouchi 1988; Onouchi 1992, over five days. Overall, fewer CAA were detected in groups receiving the higher‐dose multiple infusions (OR 0.46, 95% CI 0.32 to 0.66; P < 0.001; 6 studies, 1154 participants). See Analysis 2.3. No differences were detected between subgroups (test for subgroup differences P = 0.32).

Four studies investigated single‐infusion regimens versus multiple‐infusion regimens (Barron 1990; He 2021; Newburger 1991; Sato 1995). Sato 1995 only included participants scoring ≥ 4 on Harada score. Single doses ranged from 2 g/kg, He 2021; Newburger 1991; Sato 1995, to 1000 mg/kg, Barron 1990, total dose of IVIG. Multiple doses ranged from 2 g/kg total over two days, He 2021, or five days, Sato 1995, and 1600 mg/kg total over four days (Barron 1990; Newburger 1991). Overall, there was no clear difference in incidence of CAA between the single‐infusion and multiple‐infusion groups (OR 0.61, 95% CI 0.28 to 1.34; P = 0.22; 4 studies, 962 participants). See Analysis 2.4. No differences were detected between subgroups (test for subgroup differences P = 0.31).

When splitting participants between subgroup analyses, if groups contained an uneven number of participants so that numbers could not be equally divided, we carried out the analysis in both ways to detect possible differences in the results caused by an unequal division of the numerator and denominator. This did not cause a significant change in size or direction of effect in any of the analyses.

##### Incidence of any adverse effects after treatment initiation

Ten studies reported adverse effects (or lack of) after treatment initiation (Barron 1990; Furusho 1991A; Harada 1989; He 2021; Morikawa 1994; Newburger 1991; Onouchi 1988; Onouchi 1992; Qin 2006; Sato 1995). These included a range of reactions such as mild flushing, chills, nausea and vomiting, mild hypotension, rash, pericardial effusion, chills and noisy breathing, headache, flushing, abdominal cramping, fever, shivering, anaphylactic shock, peripheral cyanosis, hepatic dysfunction, new or worsening congestive heart failure or aortic insufficiency, pruritis, oedema and blistering, nasal congestion, cough, and erythema. No serious adverse events related to treatment were reported.

As above, we have combined data from studies following similar infusion regimens and presented all adverse effects by study in Table 4.

Two studies did not report adverse reactions (Nishihara 1988; Sakata 2007). We cannot be sure if none occurred, or if they were not recorded.

Six studies with a total of 1659 participants compared a high‐dose regimen versus a medium‐ or low‐dose regimen (described above) and reported adverse effects (Furusho 1991A; Harada 1989; He 2021; Morikawa 1994; Onouchi 1992; Qin 2006). Overall, no clear difference in the number of adverse effects was detected between regimens (OR 1.11, 95% CI 0.52 to 2.37; P = 0.78; 6 studies, 1659 participants; low‐certainty evidence). See Analysis 2.5. No differences were detected between subgroups (test for subgroup differences P = 0.93).

Two studies compared higher‐dose single‐infusion with lower‐dose single‐infusion regimens (He 2021; Qin 2006). No clear difference in incidence of adverse effects was detected between regimens (OR 1.02, 95% CI 0.14 to 7.34; P = 0.99; 2 studies, 512 participants). See Analysis 2.6.

Five studies investigated higher‐dose multiple‐infusion versus lower‐dose multiple‐infusion regimens as described above and reported adverse effects (Furusho 1991A; Harada 1989; Morikawa 1994; Onouchi 1988; Onouchi 1992). Overall, there was no clear difference detected between regimens (OR 0.98, 95% CI 0.43 to 2.22; P = 0.96; 5 studies, 1115 participants). See Analysis 2.7. No differences were detected between subgroups (test for subgroup differences P = 0.67).

Four studies investigated single‐infusion regimens versus multiple‐infusion regimens as described above and reported adverse effects (Barron 1990; He 2021; Newburger 1991; Sato 1995). Overall, there was no clear difference detected between regimens (OR 1.61, 95% CI 0.74 to 3.51; P = 0.23; 4 studies, 1005 participants). See Analysis 2.8. No differences were detected between subgroups (test for subgroup differences P = 0.52).

##### Acute coronary syndromes, such as MI or coronary thrombus

No study mentioned acute coronary syndromes, and we cannot be sure if this means none occurred, or if they were not reported (Barron 1990; Furusho 1991A; Harada 1989; He 2021; Morikawa 1994; Newburger 1991; Nishihara 1988; Onouchi 1988; Onouchi 1992; Qin 2006; Sakata 2007; Sato 1995).

##### Duration of fever (days)

Four studies comparing a high‐dose regimen versus a medium‐ or low‐dose regimen (as described above) reported on duration of fever such that data could be pooled (Harada 1989; He 2021; Qin 2006; Sakata 2007). All studies reported the mean duration of fever days (± standard deviation (SD)). Harada 1989 reported the duration of fever from treatment onset in days (± SD). The remaining three studies reported mean duration of fever days (± SD) from disease onset (He 2021; Qin 2006; Sakata 2007).

Overall, duration of fever was slightly reduced with higher‐dose regimens (MD −0.71, 95% CI −1.36 to −0.06; P = 0.03; 4 studies, 992 participants; low‐certainty evidence). Heterogeneity was detected (I^2^ = 71%), which was reduced (I^2^ = 0%) following sensitivity analysis to exclude Harada 1989 (which measured duration from treatment onset) and Sakata 2007 (study protocol was to administer different additional treatments depending on the group). No differences were detected between subgroups (test for subgroup differences P = 0.10). See Analysis 2.9.

Morikawa 1994 reported that 194/300 (65%) participants were afebrile by 72 hours in the 200 mg/kg/day for 5 days groups compared to 121/152 (80%) in the 400 mg/kg/day for 5 days group. Nishihara 1988 did not provide data, but stated that duration of febrile stages was similar between groups during treatment. Onouchi 1992 reported that more than 50% of participants in the 200 mg/kg/day for 5 days and 400 mg/kg/day for 5 days groups were afebrile by day 2. The 100 mg/kg/day for 5 days group did not reach 50% afebrile until day 4.

Furusho 1991A did not report duration of fever.

Two studies compared higher‐dose single‐infusion with lower‐dose single‐infusion regimens and reported mean duration of fever days (± SD) from disease onset (He 2021; Qin 2006). No clear difference in incidence of adverse effects was detected between regimens (MD −0.12, 95% CI −0.68 to 0.45; P = 0.69; 2 studies, 512 participants). See Analysis 2.10.

Two studies investigated higher‐dose multiple‐infusion versus lower‐dose multiple‐infusion regimens (described above) and reported mean duration of fever days (± SD) from treatment onset (Harada 1989; Onouchi 1988). Overall, no clear difference was detected between regimens (MD −0.64, 95% CI −2.88 to 1.61; P = 0.58; 2 studies, 322 participants). Heterogeneity was detected (I^2^ = 80%), and a difference was detected between subgroups (test for subgroup differences P = 0.02); this was likely due to the different infusion regimen doses compared in the subgroups. See Analysis 2.11.

Four studies investigated single‐infusion regimens versus multiple‐infusion regimens (described above) and reported on duration of fever. Newburger 1991 and Sato 1995 reported mean duration of fever from enrolment (days ± SD) and treatment onset (days ± SD), respectively. He 2021 reported mean duration of fever from disease onset (days ± SD). Barron 1990 did not report the total duration of fever, instead reporting that the maximum temperature fell an average of 1.41 ˚C in the 1 g/kg single‐infusion group compared to 0.78 ˚C in the 400 mg/kg/day multiple‐infusion group between day 1 and day 2 of treatment. For Newburger 1991, we calculated SD from SE using the RevMan Web calculator. Overall, there was no clear difference between regimens (MD −0.49, 95% CI −1.42 to 0.44; P = 0.30; 3 studies, 961 participants). Heterogeneity was detected (I^2^ = 87%), which was not reduced by sensitivity analysis to remove each study in turn. See Analysis 2.12.

##### Need for additional treatment

Four studies compared a high‐dose regimen versus a medium‐ or low‐dose regimen and reported on the need for additional treatment (He 2021; Morikawa 1994; Onouchi 1992; Sakata 2007). In He 2021, resistant participants (fever persisting for more than 24 hours after completion of IVIG infusion or recrudescent fever associated with at least two symptoms of KD after an afebrile period) received a second dose of IVIG at 2 g/kg. The numbers of participants who received a second IVIG dose were 14/141 in the 2 g/kg group compared to 12/132 in the 1000 mg/kg group. In Morikawa 1994, 17/299 participants in the 200 mg/kg/day for 5 days group compared to 2/151 in the 400 mg/kg/day for 5 days group required additional treatment with further IVIG due to recrudescent or persistent fever. In Onouchi 1992, 6/57 participants in the 500 mg/kg group and 2/52 participants in the 1000 mg/kg group received additional IVIG (based on two‐dimensional echocardiography data). No participants in the 2 g/kg group received additional IVIG. In Sakata 2007, resistant participants in the 2 g/kg group were given an additional 2 g/kg of IVIG (4/54). Participants in the 1 g/kg group were given an additional 1 g/kg (26/55), then a further 2 g/kg if required (6/55). Furusho 1991A, Harada 1989, Nishihara 1988, and Qin 2006 did not report additional treatment. We cannot be sure if this means no participants needed additional treatment, or if this was not recorded.

Pooling of data from the four studies showed a reduced need for additional treatment with the high‐dose regimens compared to the medium‐ or low‐dose regimens (OR 0.29, 95% CI 0.10 to 0.88; P = 0.03; 4 studies, 1125 participants; low‐certainty evidence). No difference was detected between subgroups (test for subgroup differences P = 0.69). Heterogeneity was detected (I^2^ = 69%), which was no longer detected (I^2^ = 8%) when sensitivity analysis was undertaken by removing Sakata 2007 from the analysis, suggesting the heterogeneity was caused by including data from this study. See Analysis 2.13.

Two studies compared higher‐dose single‐infusion with lower‐dose single‐infusion regimens (He 2021; Sakata 2007). No clear difference was detected between groups (OR 0.26, 95% CI 0.01 to 4.80; P = 0.36; 2 studies, 382 participants). Heterogeneity was again detected (I^2^ = 94%), likely for the reason described above. We were unable to undertake sensitivity analysis, as only two studies were included in the analysis. See Analysis 2.14.

Two studies comparing higher‐dose multiple‐infusion versus lower‐dose multiple‐infusion regimens reported on the need for additional treatment (Morikawa 1994; Onouchi 1992). Our analysis showed a reduced need for additional treatment in the higher‐dose multiple‐infusion compared to the lower‐dose multiple‐infusion regimens (OR 0.24, 95% CI 0.08 to 0.70; P = 0.009; 2 studies, 615 participants). No differences were detected between subgroups (test for subgroup differences P = 0.88). See Analysis 2.15.

Onouchi 1988 compared 200 mg/kg versus 400 mg/kg regimens and reported that no additional treatment was needed in either group.

Two studies comparing single‐infusion regimens versus multiple‐infusion regimens reported on the need for additional treatment (He 2021; Newburger 1991). In Newburger 1991, 5/273 participants in the 2 g/kg single‐infusion group had additional infusion compared to 8/276 in the 400 mg/kg for 4 days group. Our analysis did not detect a clear difference in the need for additional treatment between groups (OR 1.14, 95% CI 0.38 to 3.39; P = 0.81; 2 studies, 818 participants). See Analysis 2.16.

Barron 1990 and Sato 1995 did not report on additional treatment. We cannot be sure if this means no participants needed additional treatment, or if this was not recorded.

##### Length of hospital stay (days)

Three studies compared a high‐dose regimen versus a medium‐ or low‐dose regimen and reported on hospital stay in days (± SD) (He 2021; Qin 2006; Sakata 2007). Our analysis did not detect a clear difference in the length of hospital stay between groups (MD −0.24, 95% CI −0.78 to 0.30; P = 0.39; 3 studies, 752 participants; low‐certainty evidence). No differences were detected between subgroups (test for subgroup differences P = 0.40). See Analysis 2.17.

Furusho 1991A, Harada 1989, Morikawa 1994, Nishihara 1988, and Onouchi 1992 did not report this outcome.

Three studies compared higher‐dose single‐infusion with lower‐dose single‐infusion regimens (He 2021; Qin 2006; Sakata 2007). Our analysis did not detect a clear difference in the length of hospital stay between groups (MD −0.36, 95% CI −1.09 to 0.37; P = 0.33; 3 studies, 621 participants). See Analysis 2.18.

Onouchi 1988 did not report length of hospital stay, so we have no data to compare higher‐dose multiple‐infusion versus lower‐dose multiple‐infusion regimens that are not already reported above.

Four studies investigated single‐infusion regimens versus multiple‐infusion regimens, ranging from 1000 mg/kg to 2 g/kg single IVIG versus 1600 mg/kg to 2 g/kg IVIG over 2 or 4 days, and reported on hospital stay (Barron 1990; He 2021; Newburger 1991; Sato 1995).

Barron 1990 reported that participants were discharged at a median of 4 days after entry into the study in the 1 g/kg group, and at a median of 5 days in the 400 mg/kg group (P = 0.01, study author calculation). This difference at least partially reflects the fact that participants receiving the four doses required a minimum of four days of hospitalisation.

Newburger 1991 recorded that although the study protocol described 4 days of hospitalisation, 6% of participants in the 2 g/kg single‐infusion group compared to 1% of the 400 mg/kg/day for 4 days group were discharged early, the majority at day 3. Again, this must reflect that four doses required a minimum of four days of hospitalisation.

Sato 1995 only included participants scoring ≥ 4 on the Harada score and reported that the mean duration of hospital stay was 13.1 ± 6.0 days in the 2 g/kg single‐infusion group compared to 15.9 ± 7.2 days in the 400 mg/kg/day for 5 days group. It was not clear if the study authors reported SE or SD, but we have assumed these numbers to be SD. He 2021 reported that the mean ± SD of hospital stay in the 2 g/kg single‐infusion group was 8.3 ± 2.7 days compared to 8.4 ± 2.5 days in the 1000 mg/kg/day for 2 days group. We combined the data, and found no clear overall difference between single and multiple IVIG infusions (MD −1.24, 95% CI −3.86 to 1.37; P = 0.35; 2 studies, 414 participants). Heterogeneity was detected (I^2^ = 82%), which was likely due to the different participant characteristics (≥ 4 on Harada score). We were unable to undertake sensitivity analysis, as only two studies were included in the analysis. See Analysis 2.19.

##### Mortality (all‐cause)

Newburger 1991 reported details on incidence of mortality, with one death in the subacute phase of the 400 mg/kg/day for 4 days group. The cause of death was a giant aneurysm. The remaining studies did not mention mortality, and although it is likely this means there were no deaths, we cannot be sure of this (Barron 1990; Furusho 1991A; Harada 1989; He 2021; Morikawa 1994; Nishihara 1988; Onouchi 1988; Onouchi 1992; Qin 2006; Sakata 2007; Sato 1995). We assessed this evidence to be of moderate certainty.

#### Primary treatment ‐ IVIG versus prednisolone

See Table 5.

Two studies compared IVIG with prednisolone for the treatment of KD (Nonaka 1995; Yuan 2000). Nonaka 1995 randomised participants to 400 mg/kg/day IVIG for 3 days or 2 mg/kg/day of prednisolone for 5 days then 2 mg/kg/day orally until C‐reactive protein (CRP) fell to negative. Yuan 2000 randomised participants to 1 g/kg/day IVIG for 2 days or 2 mg/kg/day intravenous methylprednisolone (IVMP) for 5 days then 2 mg/kg/day orally until CRP negative. Oral dipyridamole was administered if CAA was detected.

##### Incidence of CAAs

Both studies reported CAAs. Nonaka 1995 reported CAA in the acute period, 30 days, and 3 months: 7/50, 6/50, 3/41 in the IVIG group versus 10/40, 3/31, 3/31 in the prednisolone group.

Yuan 2000 reported CAA at 2 weeks, 3, 6, 9 months, and 1‐year post‐treatment: 3/25, 2/25, 1/25, 0/25 in the IVIG group versus 3/25, 1/25, 0/25, 0/25 in the IVMP group.

Overall, there was no clear difference in the incidence of CAA in the acute phase (OR 0.60, 95% CI 0.24 to 1.48; P = 0.27; 2 studies, 140 participants; very low‐certainty evidence). See Analysis 3.1. No difference was detected by the test for subgroup differences (P = 0.49).

##### Incidence of any adverse effects after treatment initiation

Nonaka 1995 reported that no adverse effects occurred in the prednisolone group (0/40). There was one report of mild shock and one death (2/50) in the IVIG group (OR 4.18, 95% CI 0.19 to 89.48; P = 0.36; 1 study; 90 participants; low‐certainty evidence). See Analysis 3.2. Yuan 2000 did not report adverse effects. We assessed this evidence to be of low certainty.

##### Acute coronary syndromes, such as MI or coronary thrombus

Nonaka 1995 reported one MI in the IVIG group (1/50) as a cause of death. Yuan 2000 did not report any acute coronary syndromes, but we are not certain whether none occurred or if these were not reported. We assessed this evidence to be of low certainty.

##### Duration of fever (days)

Nonaka 1995 reported the time to return to normal temperature after treatment started and the overall febrile period from KD onset and found a benefit of prednisolone treatment. Yuan 2000 reported the duration of fever from treatment and found no difference between groups. We pooled the time to return to normal/duration of fever from treatment onset. Overall, there was no clear difference in duration of fever (days) after treatment was started (MD 0.68, 95% CI −0.59 to 1.94; P = 0.29; 2 studies, 140 participants; very low‐certainty evidence). Considerable heterogeneity was detected (I^2^ = 82%), likely due to differences in study design. A difference was detected by the test for subgroup differences, but given the size of the subgroups, this should be interpreted with caution (P = 0.02). See Analysis 3.3.

##### Need for additional treatment

Neither Nonaka 1995 nor Yuan 2000 reported this outcome.

##### Length of hospital stay (days)

Neither Nonaka 1995 nor Yuan 2000 reported this outcome.

##### Mortality (all‐cause)

Nonaka 1995 reported one death in the IVIG group. Cause of death was a giant aneurysm, intracranial bleeding, and MI. Yuan 2000 did not report any deaths, but we are not certain whether none occurred or if these were not reported. We assessed this evidence to be of low certainty.

#### Secondary treatment ‐ IVIG versus infliximab

Four studies compared IVIG with infliximab as a secondary treatment for KD (Burns 2008; Burns 2021; Mori 2017; Youn 2016). All participants had received primary treatment of 2 g/kg IVIG but were resistant. Resistance was described as persistent fever 48 hours to 7 days, 36 to 48 hours, 24 to 36 hours, and 36 to 48 hours respectively, after initial treatment. Participants were then randomised to treatment with further IVIG (2 g/kg) or infliximab at 5 mg/kg, Burns 2008; Mori 2017; Youn 2016, or 10 mg/kg, Burns 2021.

##### Incidence of CAAs

All four studies reported incidence of CAA, but results were difficult to interpret as many participants had CAA at start of secondary treatment.

In Burns 2008, 24 participants were resistant to initial IVIG treatment, and 12 were randomised to 5 mg/kg infliximab and 12 to further IVIG. At baseline (start of second treatment), 3/12 in the infliximab group had CAA compared to 1/12 in the IVIG group. In total, 4/12 CAA were detected in the infliximab group compared to 1/12 in the IVIG group.

In Burns 2021, IVIG‐resistant KD patients were randomised to either further treatment with a second IVIG or 10 mg/kg infliximab. At baseline (start of second treatment), 1/50 participants in the infliximab group had CAA compared to 7/49 in the IVIG group. In total, 4/50 CAA were detected in the infliximab group compared to 1/49 in the IVIG group before receiving cross‐over treatment (or third treatment).

In Mori 2017, the number of CAA detected at the start of the second treatment was unclear. They report that CAAs were found in 1/16 participants receiving 5 mg/kg infliximab (6.3%) and 3/15 participants receiving IVIG (20.0%) through to day 21. No participant had a new CAA after day 21. Mori 2017 reports that no difference in the Z max score (largest of the right coronary artery, left main coronary artery, left anterior descending artery, and left circumflex coronary artery internal diameters) was detected in participants who were evaluated for coronary artery internal diameters (Z‐score) after the start of treatment to day 56 between the infliximab group and the IVIG group.

In Youn 2016, 43 patients who were resistant to initial IVIG treatment were randomised to either a further 2 g/kg IVIG or 5 mg/kg infliximab. No CAA was detected at the start of the second treatment. CAAs developed in 4/32 participants retreated with IVIG and in 1/11 participants treated with infliximab.

Overall, there was no clear difference in the incidence of CAA between the IVIG and infliximab groups (OR 1.31, 95% CI 0.46 to 3.74; P = 0.62; 4 studies, 197 participants). See Analysis 4.1. We have presented data by the total infliximab dose as subgroups. No difference was detected by the test for subgroup differences (P = 0.60).

##### Incidence of any adverse effects after treatment initiation

All four studies reported adverse effects (Burns 2008; Burns 2021; Mori 2017; Youn 2016).

Burns 2008 reported that 5 participants (3 infliximab, 2 IVIG) experienced one or more serious adverse events (SAEs). No SAEs were related to the study medication. Eighteen participants (9 infliximab, 4 IVIG, 1 infliximab cross‐over to IVIG, and 4 IVIG cross‐overs to infliximab) experienced one or more adverse events (AEs) attributable to KD (including eczema, CAA, diastolic dysfunction, and pericardial effusion), but it was not clear in which group these occurred. Ten participants experienced 18 AE not attributable to KD: 5 participants were treated with infliximab, 1 with IVIG, 3 with IVIG followed by infliximab, and 1 with infliximab followed by IVIG. Of these AEs, only transient hepatomegaly was deemed possibly related to study drug infusion; 5/12 participants who received infliximab and 1/12 who received IVIG experienced this AE.

Burns 2021 reported 45 adverse events in the infliximab group and 65 adverse events in the second IVIG group: 24/54 participants in the infliximab group and 33/49 participants in the second IVIG group experienced at least one adverse event. There were a total of 51 SAEs: 15/54 in the infliximab group and 36/49 in second IVIG group; 10/54 participants in the infliximab group and 27/49 in the second IVIG group experienced at least one SAE. SAEs that were deemed definitely or likely related to study treatment were experienced by 0/54 participants who received only infliximab and 9/58 participants who received IVIG as either their first or second study treatment; in all nine participants the SAE experienced was haemolytic anaemia.

Mori 2017 reported that AEs occurred in 15/16 participants in the infliximab group and 15/15 participants in the IVIG group. Adverse drug reactions (anti‐double stranded DNA (anti‐dsDNA), rash, and neuralgia) occurred in 11/16 participants in the infliximab group and 10/15 participants in the IVIG group. There were no discontinuations due to AEs. There was one serious AE (relapse of KD) in an IVIG‐treated participant. Infusion reactions were reported in 0/16 infliximab and 2/15 IVIG‐treated participants. In participants with elevated anti‐dsDNA antibodies, this decreased to normal levels by the end of the trial and in participants who were followed after completion of the trial. Lupus‐like syndrome was not observed in any participant.

Youn 2016 reported that no serious AEs such as anaphylactoid reaction, severe infections, or heart failure were observed. In the IVIG group, 5/32 participants experienced infusion reaction defined as fever with or without chill that requires transient interruption of infusion. In the infliximab group, 1/11 participants experienced skin rash developed during infusion.

Overall, there was no clear difference in the incidence of AE between the IVIG and infliximab groups (OR 1.13, 95% CI 0.35 to 3.61; P = 0.84; 4 studies, 201 participants). See Analysis 4.2. We have presented data by the total infliximab dose as subgroups. No difference was detected by the test for subgroup differences (P = 0.09).

##### Acute coronary syndromes, such as MI or coronary thrombus

Burns 2008, Burns 2021, and Mori 2017 did not report acute coronary syndromes, and Youn 2016 stated that no SAEs such as heart failure were observed.

##### Duration of fever (days)

All four studies reported on duration of fever but in different ways (Burns 2008; Burns 2021; Mori 2017; Youn 2016), preventing data pooling. We have reported the results narratively below.

Burns 2008 reported the median area (and range) under temperature curve (AUC) as 1331 (1286 to 1367) in the IVIG group compared to 1333 (1313 to 1354) in the infliximab group. The study authors reported that 1/12 participants had 2 days of fever in the infliximab‐treated group; 2/12 participants had 2 days of fever in the second IVIG‐treated group; and 2/12 participants had > 2 days of fever post‐study drug administration. Participants who did not respond to treatment were crossed over to receive the second treatment. The study authors reported that the participants who crossed over had a higher AUC than those who did not cross over (P = 0.003).

Burns 2021 reported the mean (± SD) days of fever from enrolment as 1.5 (± 1.4) days in the infliximab group versus 2.5 (± 2.5) days in the IVIG group (P = 0.014). They also reported resolution of fever within 24 hours of treatment and no recurrence within 7 days in 40/52 (77%) participants in the infliximab group compared to 25/49 (51%) in the IVIG group.

Mori 2017 reported the median febrile period from start of treatment was 16 hours in the infliximab group compared to 56.1 hours in the IVIG group (no range provided). They also reported that the defervescence rate within 48 hours was greater in the infliximab group (76.7%, 95% CI 56.6 to 96.7%) compared to the IVIG group (37.0%, 95% CI 11.9 to 62.1%; P = 0.02).

Youn 2016 reported the median duration of fever and range was 6 hours (1 to 30 hours) in the infliximab group compared to 17 hours (1 to 154 hours) in the IVIG group (P = 0.044). Defervescence occurred within 24 hours in 9/11 (81.8%) participants treated with infliximab and in 18/32 (56.3%) retreated with IVIG (P = 0.098).

##### Need for additional treatment

All four studies reported on the need for additional treatment (Burns 2008; Burns 2021; Mori 2017; Youn 2016).

In Burns 2008, fever resolved in 1/12 participants who did not respond to infliximab after crossing over to IVIG. From the IVIG group, 4/12 participants required additional infliximab, and two of these went on to receive further treatment with steroids.

In Burns 2021, 9/52 participants in the infliximab group crossed over to IVIG, and 22/49 in the IVIG group crossed over to the infliximab group. Six participants with persistent fever (4/22 who received infliximab and 2/9 who received second IVIG as their cross‐over treatment) were then further treated with either ciclosporin or steroids at the discretion of the treating physician.

Mori 2017 reported that 5/16 participants in the infliximab group and 9/15 in the IVIG group were switched to another treatment (at physician's discretion) due to worsening KD.

Youn 2016 reported that 11/32 participants in the IVIG group required additional treatment (7 IVMP and 4 crossed to infliximab) compared to 1/11 in the infliximab group who crossed over to IVIG.

We were able to combine these data. Overall, fewer participants receiving infliximab required further (tertiary) treatment (OR 3.99, 95% CI 1.98 to 8.02; P < 0.001; 4 studies, 199 participants). See Analysis 4.3. We have presented data by the total infliximab dose as subgroups. No difference was detected by the test for subgroup differences (P = 0.94).

##### Length of hospital stay (days)

Presentation of the data for this outcome in the different studies precluded data pooling. We have reported the results narratively below.

Burns 2021 reported the mean ± SD days in hospital from randomisation in the infliximab group as 3.2 ± 2.1 days compared to 4.5 ± 2.5 days in the IVIG group (P < 0.001).

Youn 2016 reported the median number (and range) of days in hospital as 8 days (6 to 12) in the infliximab group compared to 10 days (6 to 14) in the IVIG group (P = 0.046).

Length of hospital stay was not reported by Burns 2008 or Mori 2017.

##### Mortality (all‐cause)

Burns 2021 reported that no deaths occurred. Burns 2008, Mori 2017, and Youn 2016 did not report incidence of mortality; although this likely means there were no deaths, we cannot be sure of this.

#### Secondary treatment ‐ IVIG versus prednisolone

Two studies compared IVIG with prednisolone as secondary treatment for KD (Miura 2008; Wang 2020). All participants received primary treatment of 2 g/kg IVIG but were resistant. Resistance was described as recrudescent or persistent fever 36 or 48 hours after initial IVIG infusion, respectively (Miura 2008; Wang 2020). Participants were then randomised to treatment with further IVIG (2 g/kg) or IVMP at 30 mg/kg/day for 3 days, Miura 2008, or intravenous methylprednisolone pulse therapy (MPT) at 15 mg/kg/day for 3 days, Wang 2020.

##### Incidence of CAAs

Both studies reported incidence of CAA (Miura 2008; Wang 2020). Results for these studies were difficult to interpret as many participants had CAA at start of secondary treatment.

Overall, there was no clear difference in the incidence of CAA between the IVIG and prednisolone groups (OR 0.46, 95% CI 0.02 to 9.18; P = 0.61; 2 studies, 75 participants). See Analysis 5.1. We have presented data by the total prednisolone dose as subgroups. No difference was detected by the test for subgroup differences (P = 0.1).

In Miura 2008, 22 participants resistant to initial IVIG treatment received secondary treatment with either IVIG or IVMP. Coronary artery dimensions and the prevalence of CAA were similar in the two groups (IVMP 2/11 versus IVIG 3/11). It is not clear at what time point the CAAs were assessed or if they were present at baseline. Miura 2008 was halted prematurely due to adverse events following IVMP infusion.

In Wang 2020, 80 IVIG‐resistant patients were randomised to either IVIG or MPT, and 33/80 had CAA before secondary treatment. They reported CAA at 7 days and 1, 3, 6, 12, and 24 months. No dropouts were CAA‐positive. The numbers of CAA in the IVIG group compared to the MPT group at each time point were as follows: 7 days: 15/40 versus 18/40; 1 month: 8/39 versus 10/40; 3 months: 4/39 versus 3/39; 6 months: 2/35 versus 4/36; 12 months: 0/28 versus 4/25; 24 months: 0/18 versus 4/15). Fewer participants returned for follow‐up ECGs as time progressed.

##### Incidence of any adverse effects after treatment initiation

Presentation of the data for this outcome in the different studies precluded data pooling. We have reported the results narratively below.

Miura 2008 was halted prematurely due to adverse events following IVMP infusion. The study authors reported increased sinus bradycardia (P = 0.01) and hyperglycaemia (P = 0.01) in the IVMP group. All adverse effects were transient. There were no convulsions, gastrointestinal symptoms, infection, or malignant arrhythmia.

Wang 2020 reported that 5/40 participants in the MPT group developed bradycardia and that there were no adverse reactions in the IVIG group (0/40) (OR 0.08, 95% CI 0.00 to 1.49; P = 0.09; 1 study, 80 participants). See Analysis 5.2.

##### Acute coronary syndromes, such as MI or coronary thrombus

Miura 2008 reported that there were no embolisms in either group. Wang 2020 did not report any acute coronary syndromes, meaning it is likely that none occurred.

##### Duration of fever (days)

Presentation of the data for this outcome in the different studies precluded data pooling. We have reported the results narratively below.

Miura 2008 reported that the proportion of febrile participants was lower in the IVMP group compared to the IVIG group until day 3 (1/11 versus 8/11; P < 0.001), but not on or after day 4 (6/11 versus 6/11). Miura 2008 also reported that the mean (SD) minimum body temperature (˚C) at 72 hours after treatment started in IVMP was 35.4˚C (± 0.4) compared to 36.1˚C (± 0.5) in the IVIG group (P = 0.002). Given the data on the proportion of febrile participants, it is likely this difference disappeared after 72 hours.

Wang 2020 reported that the mean (± SD) duration of fever (≥ 38˚C) after additional treatment was 11 ± 6.3 hours in the MPT group compared to 18 ± 4.4 hours in the IVIG group (P < 0.05).

##### Need for additional treatment

Miura 2008 did not report if any participants required additional treatment. Wang 2020 reported that 4/40 participants in the IVIG group required readmission compared to 11/40 in the MPT group. The same four participants resistant to second IVIG crossed over to MPT or received third‐line IVIG. Those not responsive to MPT received either oral methylprednisolone or IVIG.

##### Length of hospital stay (days)

Length of hospital stay was not reported by Miura 2008 or Wang 2020.

##### Mortality (all‐cause)

Miura 2008 reported that there were no sudden deaths in either group. Wang 2020 did not report any deaths, meaning it is likely that none occurred.

#### Tertiary treatment ‐ IVIG versus prednisolone

One study compared IVIG with prednisolone for the tertiary treatment of KD (Hashino 2001). Hashino 2001 randomised 17 participants who did not respond to initial IVIG 2 g/kg or secondary IVIG 1 g/kg treatment for KD to either a third dose of IVIG 1 g/kg or IVMP at 20 mg/kg/day for 3 days within the acute phase.

##### Incidence of CAAs

At baseline (before third‐line therapy), 4/17 participants had CAA (2 in each group). During the acute phase, CAA was detected in 5/8 participants in the IVIG group (2 giant aneurysms, 3 small aneurysms, 3 intact coronary arteries) and 7/9 in the IVMP group (2 giant aneurysms, 2 small aneurysms, 3 transient dilations, 2 intact coronary arteries). Given the small numbers in each group, our analysis detected no difference (OR 0.48, 95% CI 0.06 to 3.99; P = 0.49; 1 study, 17 participants). See Analysis 6.1.

##### Incidence of any adverse effects after treatment initiation

Hashino 2001 did not report adverse events, so we cannot be certain whether any occurred.

##### Acute coronary syndromes, such as MI or coronary thrombus

Hashino 2001 did not report acute coronary syndromes, so we cannot be certain whether any occurred.

##### Duration of fever (days)

Hashino 2001 reported the duration of fever (> 38.5 °C) after retreatment. In the IVIG group, the mean (± SD) duration was 4.8 ± 0.7 days compared to 1.4 ± 0.7 days in the IVMP group. Analysis suggests fever duration is reduced with IVMP treatment compared to a third dose of IVIG (MD 3.40, 95% CI 2.73 to 4.07; P < 0.001; 1 study, 17 participants). See Analysis 6.2.

##### Need for additional treatment

Hashino 2001 did not report on additional treatment.

##### Length of hospital stay (days)

Hashino 2001 did not report on the length of hospital stay.

##### Mortality (all‐cause)

Hashino 2001 did not report on mortality.

#### Additional subgroup analyses

We performed additional analyses to investigate CAA incidence by geographic distribution unless data for CAA incidence were only provided by studies from one country. Using the test for subgroup differences, we found no differences between countries for CAA incidence for the comparisons IVIG versus ASA (P = 0.17, see Analysis 7.1). For IVIG versus IVIG CAA: high‐dose versus medium‐ or low‐dose regimens (Analysis 2.1) included Chinese and Japanese studies, and subgrouping by geographical distribution did not indicate any differences (P = 0.66, see Analysis 8.1). For high‐dose single‐infusion versus lower‐dose single‐infusion regimens, subgrouping Analysis 2.2 by geographical distribution did not indicate any differences (P = 0.61, see Analysis 8.2). For higher‐dose multiple‐infusion versus lower‐dose multiple‐infusion regimens, only Japanese studies provided data, and no further subgrouping was carried out. For single‐infusion versus multiple‐infusion regimens, subgrouping Analysis 2.4 by geographical distribution did not indicate any difference (P = 0.15, see Analysis 8.3).

When splitting participants between subgroup analyses, if groups contained an uneven number of participants so that numbers could not be equally divided, we carried out the analysis in both ways to detect possible differences in the results caused by an unequal division of the numerator and denominator. This did not cause a significant change in size or direction of effect in any analysis.

#### Sensitivity analysis

As planned, we undertook sensitivity analyses to exclude studies at high risk of selection bias. This included analyses with data provided by Matsushima 1985, Nagashima 1987, or Yuan 2000.

Analysis 1.1: there was no change in overall direction or size of effect for CAA incidence (up to 30 days) in IVIG versus ASA (OR 0.66, 95% CI 0.44 to 0.98; P = 0.04).

Analysis 1.3: there was no change in overall direction or size of effect for adverse events in IVIG versus ASA (OR 0.48, 95% CI 0.13 to 1.73; P = 0.26).

Analysis 1.4: there was no change in overall direction or size of effect for duration of fever from treatment in IVIG versus ASA (MD −2.60, 95% CI −4.48 to −0.73; P = 0.007).

Analysis 1.5: there was no change in overall direction or size of effect for duration of fever from KD onset in IVIG versus ASA (MD −0.80, 95% CI −1.50 to −0.11; P = 0.02).

Analysis 3.1: there was no change in overall direction or size of effect for CAA incidence in IVIG versus prednisolone (OR 0.49, 95% CI 0.17 to 1.43; P = 0.19).

Analysis 3.3: there was a change in effect to show a possible benefit of prednisolone for duration of fever in IVIG versus prednisolone (MD 1.40, 95% CI 0.42 to −2.38; P = 0.005).

We carried out sensitivity analysis on incidence of CAA where analyses included data from studies that were at high risk of detection bias (cardiac outcomes). This included analyses with data from Burns 2008, Furusho 1984, Furusho 1991A, Furusho 1991B, Matsushima 1985, Miura 2008, Nagashima 1987, Nonaka 1995, Qin 2006, Wang 2020, Youn 2016, or Yuan 2000.

Analysis 1.1: there was no longer a difference in incidence of CAA between groups (up to 30 days) in IVIG versus ASA (OR 0.78, 95% CI 0.51 to 1.19; P = 0.24).

Analysis 1.2: there was no change in overall direction or size of effect for CAA incidence (over 6 months) in IVIG versus ASA (OR 0.88, 95% CI 0.49 to 1.56; P = 0.66).

Analysis 2.1: there was no change in overall direction or size of effect for CAA incidence high‐dose regimens versus medium‐ or low‐dose regimens (OR 0.5, 95% CI 0.30 to 0.82; P = 0.007). A difference was detected between subgroups (test for subgroup differences P = 0.01).

Analysis 2.2: there was no change in overall direction or size of effect for CAA incidence higher‐dose single‐infusion versus lower‐dose single‐infusion regimens (OR 1.01, 95% CI 0.58 to 1.79; P = 0.96).

Analysis 2.3: there was no change in overall direction or size of effect for CAA incidence higher‐dose multiple‐infusion versus lower‐dose multiple‐infusion regimens (OR 0.41, 95% CI 0.28 to 0.61; P < 0.001).

Analysis 3.1: both studies reporting on CAA incidence after treatment with IVIG versus prednisolone were excluded, so no longer estimable.

Analysis 4.1: there was no change in overall direction or size of effect for CAA incidence in IVIG versus infliximab (OR 2.04, 95% CI 0.70 to 5.95; P = 0.19).

Analysis 5.1: both studies reporting on CAA incidence after secondary treatment with IVIG versus prednisolone were excluded, so no longer estimable.

We carried out sensitivity analysis on incidence of CAA where analyses included data from studies lacking a clear description of the criteria used to diagnose KD. This included analyses with data from Miura 2008, Nagashima 1987, Nishihara 1988, Nonaka 1995, Okuni 1987A, Okuni 1987B, or Sakata 2007.

Analysis 1.1: there was no change in overall direction or size of effect for CAA incidence (up to 30 days) in IVIG versus ASA (OR 0.60, 95% CI 0.37 to 0.97; P = 0.04).

Analysis 1.2: there was no change in overall direction or size of effect for CAA incidence (over 6 months) in IVIG versus ASA (OR 0.68, 95% CI 0.37 to 1.26; P = 0.22).

Analysis 2.1: the overall effect for CAA incidence high‐dose regimens versus medium‐ or low‐dose regimens now includes both possible benefit and harm (OR 0.62, 95% CI 0.38 to 1.02; P = 0.06).

Analysis 2.2: there was no change in overall direction or size of effect for CAA incidence higher‐dose single‐infusion versus lower‐dose single‐infusion regimens (OR 0.92, 95% CI 0.59 to 1.45; P = 0.72).

Analysis 2.3: there was no change in overall direction or size of effect for CAA incidence higher‐dose multiple‐infusion versus lower‐dose multiple‐infusion regimens (OR 0.47, 95% CI 0.30 to 0.73; P = 0.001).

Analysis 3.1: there was no change in overall direction or size of effect for CAA incidence in IVIG versus prednisolone (OR 1.00, 95% CI 0.18 to 5.51; P = 1.00).

Analysis 5.1: there was no change in overall direction or size of effect for CAA incidence after secondary treatment with IVIG versus prednisolone (OR 0.08, 95% CI 0.00 to 1.64; P = 0.1).

## Discussion

We identified 31 randomised controlled trials (RCTs) involving a total of 4609 participants with Kawasaki disease (KD) that compared intravenous immunoglobulin (IVIG) with either acetylsalicylic acid (ASA), another dose or regimen of IVIG, prednisolone, or infliximab. Most studies had at least one domain at high risk of bias (blinding of participants and personnel, or blinding of outcome assessment (cardiac or non‐cardiac outcomes, or both)), and detailed descriptions of the randomisation process were infrequently provided. See Table 1; Table 3; and Table 5.

### Summary of main results

#### Primary treatment with IVIG compared to ASA for people with KD

Compared to ASA, IVIG probably reduces the incidence of coronary artery abnormalities (CAA) in people with KD. The individual studies reported a range of adverse effects, but there was little to no difference in numbers of these between groups. Evidence for incidence of acute coronary syndromes was limited, so we are uncertain of any effects. Duration of fever was probably shorter in the IVIG group, but there was little or no difference between groups in need for additional treatment. No study reported on length of hospital stay, and there was little to no difference between groups in mortality.

#### Primary treatment with IVIG compared to different infusion regimens of IVIG for people with KD

We considered a high dose to be more than 1900 mg/kg total IVIG; medium to be 1900 mg/kg to 900 mg/kg total IVIG; and low to be less than 900 mg/kg total IVIG (Ae 2020; Ae 2021). Probably fewer CAA occurred in participants treated with higher‐dose IVIG regimens compared to medium‐ or lower‐dose IVIG regimens. There was little to no difference in CAA incidence between higher‐dose single‐infusion and lower‐/medium‐dose single‐infusion regimens. Likely fewer CAA were detected in higher‐dose multiple‐infusion regimens compared to lower‐dose multiple‐infusion regimens. No clear differences were detected between single‐infusion and multiple‐infusion regimens. There was little to no difference in number of adverse effects between groups irrespective of the regimens compared. No study reported on acute coronary syndromes. Higher‐dose IVIG may reduce the duration of fever compared to medium‐ or lower‐dose regimens. No differences in duration of fever were detected between high‐dose versus medium‐dose single infusions, multiple infusions, or single‐ versus multiple‐infusion regimens. Higher‐dose regimens may reduce the need for additional treatment. We did not detect a difference in length of hospital stay between infusion regimens, and it is likely that there is little to no difference in mortality.

#### Primary treatment with IVIG compared to prednisolone for people with KD

The evidence for the comparison IVIG versus prednisolone on incidence of CAA is very uncertain, and there was little to no difference between groups in adverse effects, acute coronary syndrome, and mortality. No study reported the need for additional treatment or length of hospital stay. We are very uncertain of the impact on duration of fever, as two studies reported the duration differently and had conflicting results.

#### Secondary treatment with IVIG compared to infliximab for people with KD

We did not detect a clear difference in CAA incidence between groups treated with IVIG or infliximab following resistance to initial treatment with IVIG. Some participants had CAA at the start of secondary treatment. Similarly, no clear differences in the number of adverse effects were detected. Limited data were available for acute coronary syndromes and duration of fever, which prevented us from drawing any conclusions. Analysis indicated that there may be a reduced need for further (tertiary) treatment in the infliximab group. Given that the participants in this analysis were IVIG resistant, this is not surprising. We are unable to draw any conclusions about length of hospital stay because of differences in outcome reporting between studies. It is likely there were no deaths in either group.

#### Secondary treatment with IVIG compared to prednisolone for people with KD

We did not detect a clear difference in CAA incidence between groups treated with IVIG or prednisolone following resistance to initial treatment with IVIG. Some participants had CAA at the start of secondary treatment. One study was halted due to adverse effects in the prednisolone group, and the second study reported increased events in the prednisolone group. Data for acute coronary syndromes were limited, and we could not pool data for duration of fever, though both studies reported a shorter fever duration in the prednisolone group. One study reported that fewer participants required additional (tertiary) treatment in the IVIG group. Length of hospital stay was not reported, and no deaths were reported.

#### Tertiary treatment with IVIG compared to prednisolone for people with KD

One study investigated IVIG compared to prednisolone in participants who were resistant to both initial and secondary IVIG treatment. Evidence was limited, and no clear differences between groups in CAA incidence were detected. The study authors reported that duration of fever was reduced in the prednisolone group; however, this result should be interpreted with caution given the study limitations. No other outcomes of interest were reported.

### Overall completeness and applicability of evidence

We have attempted to find all available published and unpublished randomised trials for the use of IVIG in people with KD. We included a total of 31 studies in quantitative synthesis, and we believe that the included trials are comprehensive. However, it should be noted that we were not able to obtain data for all outcomes defined in this systematic review.

As we restricted our inclusion criteria to evidence provided by RCTs, the identified studies provided a low overall number of events, and outcomes tended not to be reported for later time points, as study durations were generally short. We identified a variety of comparisons using a range of doses, recording different time points and reporting different outcomes. Specific details on baseline measurements of outcomes, for example CAA, were not always provided. These factors limit the applicability and completeness of our findings, as we are not accounting for the large amount of non‐randomised evidence gathered by long‐term cohort studies such as the Kawasaki Disease Nationwide Survey (Ae 2021).

#### Primary treatment

The available evidence indicates that high‐dose IVIG regimens are associated with a low risk of CAA formation, need for additional therapy, and prolonged duration of fever. This suggests that high‐dose IVIG regimens would be beneficial compared to medium‐ or low‐dose IVIG regimens, which are superior to ASA regimens. There were no clinically significant differences in incidence of adverse events between groups, which suggests there is little concern about the safety of IVIG. Regarding acute coronary syndromes and mortality, we could not find signals of effectiveness because the number of events was rarely observed during the observation period of the trials.

#### Secondary or tertiary treatment

We found no clear evidence to support the effectiveness of IVIG compared to infliximab or prednisolone for the incidence of CAA, adverse effects, duration of fever, length of hospital stay, acute coronary syndrome, or mortality due to lack of sufficient statistical power in the obtained data.

### Quality of the evidence

#### Primary treatment with IVIG compared to ASA for people with KD

See Table 1.

For the outcome CAA, we downgraded the certainty of the evidence by one level to moderate, as four studies included in the analysis were at high risk of bias (selection, performance, detection of cardiac outcomes, or reporting bias), and sensitivity analysis to exclude those studies at high risk of detection bias caused a change in effect size. For adverse effects, we downgraded the certainty of the evidence by one level due to concerns related to risk of bias (six studies were at high risk of selection, performance, or detection of non‐cardiac outcomes bias), one level for inconsistency (heterogeneity was high: I^2^ = 73%), and one level for imprecision (confidence intervals include appreciable benefit or harm) to very low. For acute coronary syndromes, we downgraded the certainty of the evidence by one level due to concerns related to risk of bias (one study at high risk of selection/detection bias) and one level for imprecision (small numbers of events and participants, and confidence intervals include appreciable benefit or harm) to low. For duration of fever, we downgraded the certainty of the evidence by a total of one level due to concerns related to risk of bias (three studies were at high risk of selection/performance/detection bias) and imprecision (small number of participants) to moderate. For the outcome need for additional treatment, we downgraded the certainty of the evidence by one level due to concerns related to risk of bias (two studies at high risk of selection/detection of non‐cardiac outcomes bias) and one level for imprecision (small numbers of participants and events, and confidence intervals include appreciable benefit or harm) to low. No studies reported length of hospital stay, preventing an assessment of the certainty of the evidence. For mortality, we downgraded the certainty of the evidence by one level due to concerns related to risk of bias (two studies at high risk of selection/detection bias) and one level for imprecision (small numbers of participants and events) to low.

#### Primary treatment with IVIG compared to different infusion regimens of IVIG for people with KD

See Table 3.

For CAA, we downgraded the certainty of the evidence by one level due to concerns related to risk of bias (two studies at high risk of detection of cardiac outcomes or performance bias) to moderate. For adverse effects, we downgraded the certainty of the evidence by one level due to concerns related to risk of bias (five studies at high risk of detection of non‐cardiac outcomes or performance bias, or both) and one level for imprecision (small numbers of events, and confidence intervals include appreciable benefit or harm) to low. No studies reported acute coronary syndromes, preventing an assessment of the certainty of the evidence. For duration of fever, we downgraded the certainty of the evidence by one level due to concerns related to risk of bias (three studies at high risk of detection of non‐cardiac outcomes) and one level for inconsistency (I^2^ = 71%) to low. Sensitivity analysis indicated that this was explained by study differences (reporting from KD onset or treatment onset). For need for additional treatment, we downgraded the certainty of the evidence by one level due to concerns related to risk of bias (two studies at high risk of detection of cardiac outcomes or performance bias) and one level for inconsistency (I^2^ = 69%) to low. For length of hospital stay, we downgraded the certainty of the evidence by a total of two levels to low due to concerns related to risk of bias and imprecision (confidence intervals include appreciable benefit or harm) to low. For mortality, we downgraded the certainty of the evidence by one level due to imprecision (small number of events reported) to moderate.

#### Primary treatment with IVIG compared to prednisolone for people with KD

See Table 5.

For CAA, we downgraded the certainty of the evidence by two levels due to concerns related to risk of bias (both studies at high risk of selection/detection bias) and one level for imprecision (small numbers of participants) to very low. For adverse effects, we downgraded the certainty of the evidence by one level due to concerns related to risk of bias (study at risk of detection of non‐cardiac outcome bias) and one level for imprecision (small numbers of events and participants, and wide confidence interval) to low. For acute coronary syndrome, we downgraded the certainty of the evidence by one level due to concerns related to risk of bias (study at high risk of detection bias) and one level for imprecision (small numbers of events and participants) to low. For duration of fever, we downgraded the certainty of the evidence by one level due to concerns related to risk of bias (both studies at high risk of selection or detection bias), one level for inconsistency (I^2^ = 82%), and one level for imprecision (small numbers of participants) to very low. Need for additional treatment and length of hospital stay were not reported for this comparison. For mortality, we downgraded the certainty of the evidence by one level for risk of bias and one level for imprecision (small numbers of events and participants) to low.

### Potential biases in the review process

We followed standard Cochrane procedures when developing the protocol for this review and in conducting the review. We attempted to contact the study authors where possible to obtain further information. We included studies irrespective of language. We identified additional studies in reference lists of reviews that had not been detected by our searches. These studies have been placed in awaiting classification until we are able to confirm that they meet the inclusion criteria of this Cochrane Review. It is possible that not including data from these studies has impacted the overall review findings. We decided to pool studies that evaluated different doses of treatments using subgroup analysis. We used subgroups for high‐/medium‐/low‐dose IVIG groups, as they represent clinically relevant regimens and allowed important comparisons to be investigated. We decided to include all adverse effects reported by studies in the data analyses, even though studies likely had different definitions of what an adverse effect was. We believe any bias overall would have been minimal, and any differences between treatment groups in individual studies would have been detected. We were limited in only including data from RCTs, and no large‐scale randomised studies have been carried out. This makes detection of rare events unlikely and decreased our confidence in the review findings. As discussed below, further evidence is available from large epidemiological studies.

### Agreements and disagreements with other studies or reviews

This review replaces an earlier Cochrane Review (Oates‐Whitehead 2003). Our findings are in agreement with that review. We have included data from more recent studies and widened the scope to include studies also comparing IVIG with tumour necrosis factor‐alpha blockers and corticosteroids, which reflects current clinical options. This review indicates that high‐dose IVIG regimens are probably effective in preventing the formation of CAAs up to 30 days. In Japan, the nationwide survey of Kawasaki disease, which includes more than 90% of new KD patients, has been ongoing since 1970. Based on the latest Kawasaki Disease Nationwide Survey (Ae 2021), chronological changes in the proportion of KD patients treated with IVIG by dose regimens and incidences of CAAs are shown in Figure 4. The chronological trend based on large epidemiological studies shows clearly an inverse relationship between a decrease in CAAs and an increase in the proportion of IVIG administration, especially IVIG 2 g/kg single‐infusion regimen. In the 1980s, most KD patients were treated with ASA alone, and 16% to 18% of KD patients had CAAs. In the early 1990s, more than 80% of KD patients received low‐ to medium‐dose IVIG multiple‐infusion regimens, and the incidence of CAAs decreased to approximately 12%. Since the late 1990s, IVIG had been replaced by 2 g/kg single infusion, and the incidence of CAAs decreased to approximately 4% in the 2000s. Based on these data, the incidence of CAAs decreased by approximately 30% from the ASA era to the low‐ to medium‐dose IVIG era, and then by approximately 60% in the high‐dose IVIG era. The results from this review showed an odds ratio (OR) of 0.60 (95% confidence interval (CI) 0.41 to 0.87) for ASA regimens versus IVIG regimens and an OR of 0.60 (95% CI 0.40 to 0.89) for high‐dose IVIG regimens versus low‐ or medium‐dose IVIG regimens. Based on these findings, the evidence for the preventive effect of IVIG on CAAs is robust and reproducible in large epidemiological studies. Several recent guidelines recommend high‐dose IVIG single infusion as the primary treatment for acute KD (de Graeff 2019; McCrindle 2017; Miura 2021).

**4:**
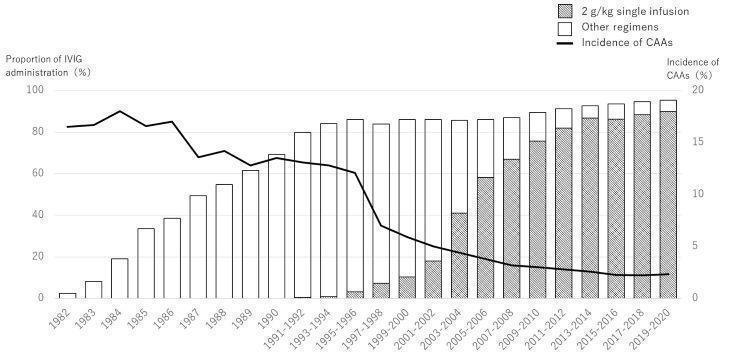
Chronological changes of proportion of intravenous immunoglobulin (IVIG) administration and incidence of coronary artery abnormalities (CAAs) from the Nationwide Survey of Kawasaki Disease in Japan. Created by review author (TK) with data from the Nationwide Survey (with permission from the Director of the Nationwide Survey Kawasaki Disease in Japan).

In this review, we could not show an association between IVIG and mortality because of the short observation period of the clinical trial and the few events that occurred. The National Kawasaki Disease Study has continuously investigated the mortality of people with KD since 1967 and found that mortality was about 2% in the 1960s, 0.8% in the 1970s, 0.2% in the 1980s, 0.1% in the 1990s, 0.04% in the 2000s, and 0.02% in the 2010s (Ae 2021). The rate of death has been declining at a rate of about half a per cent per decade. It is reasonable to assume that the reduction in mortality rates observed in these large observational studies is related to the widespread use of immunoglobulins.

With regard to need for additional treatment, adverse effects, and duration of fever, no large‐scale epidemiological studies have been conducted continuously since the 1980s, therefore it is difficult to compare the association between IVIG and these effects on the basis of large studies.

## Authors' conclusions

**Implications for practice**

Our findings are in keeping with current guideline recommendations. The strength of our conclusions is limited by restricting to randomised controlled trial (RCT) data. The included RCTs investigated a variety of comparisons, and the small number of events observed during the study periods limited the detection of effects. The available evidence indicated that high‐dose intravenous immunoglobulin (IVIG) regimens are probably associated with a reduced risk of coronary artery abnormalities (CAAs) formation compared to acetylsalicylic acid (ASA) or medium‐ or low‐dose IVIG regimens. Incidence of adverse events did not show clinically significant differences, which suggests there is little concern about the safety of IVIG. Compared to ASA, high‐dose IVIG probably reduced duration of fever, but there was little or no difference detected in the need for additional treatment. Compared to medium‐ or low‐dose IVIG, there may be reduced duration of fever and reduced need for additional treatment. We were unable to draw any conclusions regarding acute coronary syndromes, mortality, and length of hospital stay. We were also unable to determine any effects when comparing IVIG treatment with tumour necrosis factor (TNF)‐blockers or corticosteroids, or in people who were resistant to initial IVIG treatment.

**Implications for research**

Although we included 31 RCTs in the review, we found limited short‐ and long‐term RCT evidence for most outcomes, especially acute coronary syndromes, mortality, and length of hospital stay. Further well‐designed RCTs would both provide missing evidence and strengthen our certainty in the effects detected. This is the case for IVIG compared to ASA, other IVIG regimens, and other treatments for Kawasaki disease including TNF‐blockers and corticosteroids. Any such studies should report clinically relevant, clearly defined outcomes in a standardised way and report both short‐ and long‐term follow‐up periods. In order to assess these outcomes appropriately, randomised trials would need to include very large numbers of people. However, as large epidemiological studies are ongoing, it is unlikely that new RCTs investigating different IVIG regimens or comparing IVIG with ASA will begin. Ongoing RCTs identified in our searches compare IVIG with newer treatments (interleukin‐1 receptor antagonists) or are focused on IVIG‐resistant patients. Evidence from RCTs comparing IVIG with other treatments is important for the proportion of patients who fail to respond to initial IVIG treatment, as they are at most risk of serious complications from Kawasaki disease.

## History

Protocol first published: Issue 6, 2021

## Notes

For the purpose of open access, the author has applied a Creative Commons Attribution (CC‐BY) licence to any Author Accepted Manuscript version arising from this submission.

Parts of the Methods section of this review are based on a standard template established by Cochrane Vascular.

## Appendices

### Appendix 1. Sources searched and search strategies

**Table:**

**Source**	**Search strategy**	**Hits retrieved**
VASCULAR REGISTER IN CRSW (date of most recent search 26 April 2022)	#1 Mucocutaneous Lymph Node Syndrome AND INREGISTER #2 kawasaki* AND INREGISTER #3 #1 OR #2 #4 Immunoglobulin* AND INREGISTER #5 Ig* AND INREGISTER #6 IVIG AND INREGISTER #7 civacir or flebogamma or gamunex or carimune or gammagard or octagam or privigen AND INREGISTER #8 #7 OR #6 OR #5 OR #4 #9 #3 AND #8	July 2021: 110 April 2022: 3
CENTRAL via CRSO (date of most recent search 26 April 2022)	#1 MESH DESCRIPTOR Mucocutaneous Lymph Node Syndrome EXPLODE ALL TREES 105 #2 (mucocutan* adj syndrome*):TI,AB,KY 195 #3 kawasaki*:TI,AB,KY 320 #4 (Mucocutaneous Lymph Node Syndrome):TI,AB,KY 189 #5 #1 OR #2 OR #3 OR #4 339 #6 MESH DESCRIPTOR Immunoglobulins, Intravenous EXPLODE ALL TREES 831 #7 immunoglobulin*:TI,AB,KY 13976 #8 Ig*:TI,AB,KY 25033 #9 IVIG:TI,AB,KY 1331 #10 (civacir or flebogamma or gamunex or carimune or gammagard or octagam or privigen):TI,AB,KY 145 #11 #6 OR #7 OR #8 OR #9 OR #10 32457 #12 #5 AND #11 163	July 2021: 163 April 2022: 11
ClinicalTrials.gov (date of most recent search 26 April 2022)	Mucocutaneous Lymph Node Syndrome OR kawasaki OR Mucocutaneous Syndrome | Immunoglobulins OR Immunoglobulin OR Ig OR IVIG OR civacir or flebogamma or gamunex or carimune or gammagard or octagam or privigen	July 2021: 22 April 2022: 1
ICTRP Search Portal (date of most recent search 26 April 2022)	Mucocutaneous Lymph Node Syndrome OR kawasaki OR Mucocutaneous Syndrome | Immunoglobulins OR Immunoglobulin OR Ig OR IVIG OR civacir or flebogamma or gamunex or carimune or gammagard or octagam or privigen	July 2021: 38 April 2022: 9
MEDLINE (Ovid MEDLINE Epub Ahead of Print, In‐Process & Other Non‐Indexed Citations, Ovid MEDLINE Daily and Ovid MEDLINE) 1946 to present (date of most recent search 26 April 2022)	1 Mucocutaneous Lymph Node Syndrome/ 2 (mucocutan* adj syndrome*).ti,ab. 3 kawasaki*.ti,ab. 4 "Mucocutaneous Lymph Node Syndrome".ti,ab. 5 or/1‐4 6 exp Immunoglobulins, Intravenous/ 7 immunoglobulin*.ti,ab. 8 Ig*.ti,ab. 9 IVIG.ti,ab. 10 (civacir or flebogamma or gamunex or carimune or gammagard or octagam or privigen).ti,ab. 11 or/6‐10 12 5 and 11 13 randomized controlled trial.pt. 14 controlled clinical trial.pt. 15 randomized.ab. 16 placebo.ab. 17 drug therapy.fs. 18 randomly.ab. 19 trial.ab. 20 groups.ab. 21 or/13‐20 22 exp animals/ not humans.sh. 23 21 not 22 24 12 and 23	July 2021: 1156 April 2022: 196
Embase via Ovid (date of most recent search 26 April 2022)	1 exp mucocutaneous lymph node syndrome/ 2 (mucocutan* adj syndrome*).ti,ab. 3 kawasaki*.ti,ab. 4 "Mucocutaneous Lymph Node Syndrome".ti,ab. 5 or/1‐4 6 exp immunoglobulin/ 7 immunoglobulin*.ti,ab. 8 Ig*.ti,ab. 9 IVIG.ti,ab. 10 (civacir or flebogamma or gamunex or carimune or gammagard or octagam or privigen).ti,ab. 11 or/6‐10 12 5 and 11 13 randomized controlled trial/ 14 controlled clinical trial/ 15 random$.ti,ab. 16 randomization/ 17 intermethod comparison/ 18 placebo.ti,ab. 19 (compare or compared or comparison).ti. 20 ((evaluated or evaluate or evaluating or assessed or assess) and (compare or compared or comparing or comparison)).ab. 21 (open adj label).ti,ab. 22 ((double or single or doubly or singly) adj (blind or blinded or blindly)).ti,ab. 23 double blind procedure/ 24 parallel group$1.ti,ab. 25 (crossover or cross over).ti,ab. 26 ((assign$ or match or matched or allocation) adj5 (alternate or group$1 or intervention$1 or patient$1 or subject$1 or participant$1)).ti,ab. 27 (assigned or allocated).ti,ab. 28 (controlled adj7 (study or design or trial)).ti,ab. 29 (volunteer or volunteers).ti,ab. 30 trial.ti. 31 or/13‐30 32 12 and 31	July 2021: 698 April 2022: 143
CINAHL via EBSCO (date of most recent search 26 April 2022)	S28 S12 AND S27 S27 S13 OR S14 OR S15 OR S16 OR S17 OR S18 OR S19 OR S20 OR S21 OR S22 OR S23 OR S24 OR S25 OR S26 S26 MH "Random Assignment" S25 MH "Triple‐Blind Studies" S24 MH "Double‐Blind Studies" S23 MH "Single‐Blind Studies" S22 MH "Crossover Design" S21 MH "Factorial Design" S20 MH "Placebos" S19 MH "Clinical Trials" S18 TX "multi‐centre study" OR "multi‐center study" OR "multicentre study" OR "multicenter study" OR "multi‐site study" S17 TX crossover OR "cross‐over" S16 AB placebo* S15 TX random* S14 TX trial* S13 TX "latin square" S12 S5 AND S11 S11 S6 OR S7 OR S8 OR S9 OR S10 S10 TX civacir or flebogamma or gamunex or carimune or gammagard or octagam or privigen S9 TX IVIG S8 TX Ig* S7 TX immunoglobulin* S6 (MH "Immunoglobulins, Intravenous") S5 S1 OR S2 OR S3 OR S4 S4 TX Mucocutaneous Lymph Node Syndrome S3 TX kawasaki* S2 TX mucocutan* N2 syndrome* S1 (MH "Mucocutaneous Lymph Node Syndrome")	July 2021: 113 April 2022: 16
TOTAL before deduplication		July 2021: 2300 April 2022: 379
TOTAL after deduplication		July 2021: 1747 April 2022: 320
TOTAL 2021 and 2022 after deduplication		April 2022: 1935

## Data and analyses

**Comparison 1:** Primary treatment ‐ IVIG (and ASA) versus ASA

Outcome or subgroup title	No. of studies	No. of participants	Statistical method	Effect size
1.1 CAA: total dose of IVIG subgrouped by single or multiple infusion (up to 30 days)	11	1437	Odds Ratio (M‐H, Random, 95% CI)	0.60 [0.41, 0.87]
1.1.1 100 mg/kg over single infusion	1	211	Odds Ratio (M‐H, Random, 95% CI)	1.08 [0.60, 1.92]
1.1.2 1000 mg/kg over single infusion	1	84	Odds Ratio (M‐H, Random, 95% CI)	0.74 [0.29, 1.90]
1.1.3 600 mg/kg over 3 days	2	153	Odds Ratio (M‐H, Random, 95% CI)	1.77 [0.58, 5.41]
1.1.4 1200 mg/kg over 3 days	3	318	Odds Ratio (M‐H, Random, 95% CI)	0.46 [0.27, 0.76]
1.1.5 1600 mg/kg over 4 days	1	149	Odds Ratio (M‐H, Random, 95% CI)	0.29 [0.10, 0.84]
1.1.6 500 mg/kg over 5 days	1	256	Odds Ratio (M‐H, Random, 95% CI)	0.57 [0.30, 1.09]
1.1.7 1 g/kg over 5 days	1	72	Odds Ratio (M‐H, Random, 95% CI)	0.54 [0.13, 2.23]
1.1.8 1 g/kg over 5 days (no ASA)	1	75	Odds Ratio (M‐H, Random, 95% CI)	0.32 [0.08, 1.34]
1.1.9 2 g/kg over 5 days	2	119	Odds Ratio (M‐H, Random, 95% CI)	0.25 [0.10, 0.65]
1.2 CAA: total dose of IVIG subgrouped by single or multiple infusion (≥ 6 months)	7	679	Odds Ratio (M‐H, Random, 95% CI)	0.79 [0.45, 1.38]
1.2.1 100 mg/kg over single infusion	1	211	Odds Ratio (M‐H, Random, 95% CI)	1.63 [0.43, 6.21]
1.2.2 1000 mg/kg over single infusion	1	84	Odds Ratio (M‐H, Random, 95% CI)	0.73 [0.14, 3.87]
1.2.3 600 mg/kg over 3 days	2	153	Odds Ratio (M‐H, Random, 95% CI)	1.32 [0.45, 3.82]
1.2.4 1200 mg/kg over 3 days	2	182	Odds Ratio (M‐H, Random, 95% CI)	0.59 [0.18, 1.92]
1.2.5 1600 mg/kg over 4 days	1	24	Odds Ratio (M‐H, Random, 95% CI)	0.36 [0.05, 2.37]
1.2.6 2 g/kg over 5 days	1	25	Odds Ratio (M‐H, Random, 95% CI)	0.15 [0.01, 1.50]
1.3 Adverse effects: total dose of IVIG subgrouped by single or multiple infusion	10	1376	Odds Ratio (M‐H, Random, 95% CI)	0.57 [0.17, 1.89]
1.3.1 100 mg/kg over single infusion	1	214	Odds Ratio (M‐H, Random, 95% CI)	Not estimable
1.3.2 1000 mg/kg over single infusion	1	84	Odds Ratio (M‐H, Random, 95% CI)	0.45 [0.12, 1.75]
1.3.3 600 mg/kg over 3 days	2	165	Odds Ratio (M‐H, Random, 95% CI)	0.08 [0.01, 0.49]
1.3.4 1200 mg/kg over 3 days	3	325	Odds Ratio (M‐H, Random, 95% CI)	1.63 [0.08, 34.63]
1.3.5 1600 mg/kg over 4 days	1	168	Odds Ratio (M‐H, Random, 95% CI)	1.44 [0.44, 4.72]
1.3.6 500 mg/kg over 5 days	1	295	Odds Ratio (M‐H, Random, 95% CI)	Not estimable
1.3.7 2 g/kg over 5 days	2	125	Odds Ratio (M‐H, Random, 95% CI)	0.30 [0.04, 2.34]
1.4 Duration of fever from treatment onset (days): total dose of IVIG subgrouped by single or multiple infusion	3	307	Mean Difference (IV, Random, 95% CI)	‐4.00 [‐5.06, ‐2.93]
1.4.1 600 mg/kg over 3 days	1	72	Mean Difference (IV, Random, 95% CI)	‐2.90 [‐5.54, ‐0.26]
1.4.2 1200 mg/kg over 3 days	2	201	Mean Difference (IV, Random, 95% CI)	‐3.84 [‐6.35, ‐1.34]
1.4.3 2 g/kg over 5 days	1	34	Mean Difference (IV, Random, 95% CI)	‐4.20 [‐5.77, ‐2.63]
1.5 Duration of fever from KD onset (days): total dose of IVIG subgrouped by single or multiple infusion	7	693	Mean Difference (IV, Random, 95% CI)	‐1.60 [‐2.69, ‐0.52]
1.5.1 1000 mg/kg over single infusion	1	84	Mean Difference (IV, Random, 95% CI)	0.00 [‐1.54, 1.54]
1.5.2 600 mg/kg over 3 days	2	164	Mean Difference (IV, Random, 95% CI)	‐1.05 [‐3.56, 1.45]
1.5.3 1200 mg/kg over 3 days	3	318	Mean Difference (IV, Random, 95% CI)	‐2.40 [‐4.62, ‐0.19]
1.5.4 2 g/kg over 5 days	2	127	Mean Difference (IV, Random, 95% CI)	‐1.79 [‐2.99, ‐0.60]
1.6 Need for additional treatment: total dose of IVIG subgrouped by single or multiple infusion	3	272	Odds Ratio (M‐H, Random, 95% CI)	0.27 [0.05, 1.57]
1.6.1 600 mg/kg over 3 days	1	73	Odds Ratio (M‐H, Random, 95% CI)	0.16 [0.01, 4.03]
1.6.2 1200 mg/kg over 3 days	1	72	Odds Ratio (M‐H, Random, 95% CI)	0.09 [0.00, 2.01]
1.6.3 2 g/kg over 5 days	2	127	Odds Ratio (M‐H, Random, 95% CI)	1.02 [0.06, 16.85]

**Comparison 2:** Primary treatment ‐ IVIG versus IVIG

Outcome or subgroup title	No. of studies	No. of participants	Statistical method	Effect size
2.1 CAA: high‐dose regimens vs medium‐ or low‐dose regimens	8	1824	Odds Ratio (M‐H, Random, 95% CI)	0.60 [0.40, 0.89]
2.1.1 High‐dose single infusion vs medium‐dose single infusion	3	555	Odds Ratio (M‐H, Random, 95% CI)	0.85 [0.55, 1.31]
2.1.2 High‐dose multiple infusion vs medium‐dose single infusion	1	195	Odds Ratio (M‐H, Random, 95% CI)	1.09 [0.47, 2.57]
2.1.3 High‐dose multiple infusion vs medium‐dose multiple infusion	4	649	Odds Ratio (M‐H, Random, 95% CI)	0.53 [0.20, 1.41]
2.1.4 High‐dose multiple infusion vs low‐dose multiple infusion	4	425	Odds Ratio (M‐H, Random, 95% CI)	0.36 [0.20, 0.64]
2.2 CAA: higher‐dose single‐infusion regimens vs lower‐dose single‐infusion regimens	3		Odds Ratio (M‐H, Random, 95% CI)	Subtotals only
2.2.1 High‐dose single infusion versus medium‐dose single infusion	3	621	Odds Ratio (M‐H, Random, 95% CI)	0.88 [0.59, 1.32]
2.3 CAA: higher‐dose multiple‐infusion regimens vs lower‐dose multiple‐infusion regimens	6	1154	Odds Ratio (M‐H, Random, 95% CI)	0.46 [0.32, 0.66]
2.3.1 High‐dose multiple infusion vs medium‐dose multiple infusion	4	583	Odds Ratio (M‐H, Random, 95% CI)	0.49 [0.20, 1.19]
2.3.2 High‐dose multiple infusion vs low‐dose multiple infusion	4	366	Odds Ratio (M‐H, Random, 95% CI)	0.35 [0.20, 0.62]
2.3.3 Medium‐dose multiple infusion vs low‐dose multiple infusion	4	205	Odds Ratio (M‐H, Random, 95% CI)	0.66 [0.36, 1.23]
2.4 CAA: single‐infusion regimens vs multiple‐infusion regimens	4	962	Odds Ratio (M‐H, Random, 95% CI)	0.61 [0.28, 1.34]
2.4.1 High‐dose single infusion vs high‐dose multiple infusion	3	918	Odds Ratio (M‐H, Random, 95% CI)	0.54 [0.23, 1.27]
2.4.2 Medium‐dose single infusion vs high‐dose multiple infusion	1	44	Odds Ratio (M‐H, Random, 95% CI)	2.10 [0.18, 25.01]
2.5 Adverse effects: high‐dose regimens vs medium‐ or low‐dose regimens	6	1659	Odds Ratio (M‐H, Random, 95% CI)	1.11 [0.52, 2.37]
2.5.1 High‐dose single infusion vs medium‐dose single infusion	2	446	Odds Ratio (M‐H, Random, 95% CI)	1.02 [0.14, 7.34]
2.5.2 High‐dose multiple infusion vs medium‐dose single infusion	1	195	Odds Ratio (M‐H, Random, 95% CI)	1.55 [0.06, 38.63]
2.5.3 High‐dose multiple infusion vs medium‐dose multiple infusion	3	623	Odds Ratio (M‐H, Random, 95% CI)	1.22 [0.50, 3.01]
2.5.4 High‐dose multiple infusion vs low‐dose multiple infusion	3	395	Odds Ratio (M‐H, Random, 95% CI)	0.53 [0.05, 5.93]
2.6 Adverse effects: higher‐dose single‐infusion regimens vs lower‐dose single‐infusion regimens	2		Odds Ratio (M‐H, Random, 95% CI)	Subtotals only
2.6.1 High‐dose single infusion vs medium‐dose single infusion	2	512	Odds Ratio (M‐H, Random, 95% CI)	1.02 [0.14, 7.34]
2.7 Adverse effects: higher‐dose multiple‐infusion regimens vs lower‐dose multiple‐infusion regimens	5	1115	Odds Ratio (M‐H, Random, 95% CI)	0.98 [0.43, 2.22]
2.7.1 High‐dose multiple infusion vs medium‐dose multiple infusion	3	572	Odds Ratio (M‐H, Random, 95% CI)	1.16 [0.47, 2.86]
2.7.2 High‐dose multiple infusion vs low‐dose multiple infusion	3	345	Odds Ratio (M‐H, Random, 95% CI)	0.53 [0.05, 5.93]
2.7.3 Medium‐dose multiple infusion vs low‐dose multiple infusion	3	198	Odds Ratio (M‐H, Random, 95% CI)	0.33 [0.01, 8.39]
2.8 Adverse effects: single‐infusion regimens vs multiple‐infusion regimens	4	1005	Odds Ratio (M‐H, Random, 95% CI)	1.61 [0.74, 3.51]
2.8.1 High‐dose single infusion vs high‐dose multiple infusion	3	961	Odds Ratio (M‐H, Random, 95% CI)	1.32 [0.49, 3.56]
2.8.2 Medium‐dose single infusion vs high‐dose multiple infusion	1	44	Odds Ratio (M‐H, Random, 95% CI)	2.22 [0.63, 7.82]
2.9 Duration of fever (days): high‐dose regimens vs medium‐ or low‐dose regimens	4	992	Mean Difference (IV, Random, 95% CI)	‐0.71 [‐1.36, ‐0.06]
2.9.1 High‐dose single infusion vs medium‐dose single infusion	3	555	Mean Difference (IV, Random, 95% CI)	‐0.62 [‐1.56, 0.32]
2.9.2 High‐dose multiple infusion vs medium‐dose single infusion	1	195	Mean Difference (IV, Random, 95% CI)	‐0.30 [‐0.94, 0.34]
2.9.3 High‐dose multiple infusion vs low‐dose multiple infusion	1	242	Mean Difference (IV, Random, 95% CI)	‐1.70 [‐2.81, ‐0.59]
2.10 Duration of fever (days): higher‐dose single‐infusion regimens vs lower‐dose single‐infusion regimens	2		Mean Difference (IV, Random, 95% CI)	Subtotals only
2.10.1 High‐dose single infusion vs medium‐dose single infusion	2	512	Mean Difference (IV, Random, 95% CI)	‐0.12 [‐0.68, 0.45]
2.11 Duration of fever (days): higher‐dose multiple‐infusion regimens vs lower‐dose multiple‐infusion regimens	2	322	Mean Difference (IV, Random, 95% CI)	‐0.64 [‐2.88, 1.61]
2.11.1 High‐dose multiple infusion vs low‐dose multiple infusion	1	242	Mean Difference (IV, Random, 95% CI)	‐1.70 [‐2.81, ‐0.59]
2.11.2 Medium‐dose multiple infusion vs low‐dose multiple infusion	1	80	Mean Difference (IV, Random, 95% CI)	0.60 [‐1.07, 2.27]
2.12 Duration of fever (days): single‐infusion regimens vs multiple‐infusion regimens	3	961	Mean Difference (IV, Random, 95% CI)	‐0.49 [‐1.42, 0.44]
2.12.1 High‐dose single infusion vs high‐dose multiple infusion ‐ duration from onset of disease	1	267	Mean Difference (IV, Random, 95% CI)	0.40 [‐0.09, 0.89]
2.12.2 High‐dose single infusion vs high‐dose multiple infusion ‐ duration from onset of treatment	2	694	Mean Difference (IV, Random, 95% CI)	‐1.09 [‐2.63, 0.45]
2.13 Need for additional treatment: high‐dose regimens vs medium‐ or low‐dose regimens	4	1125	Odds Ratio (M‐H, Random, 95% CI)	0.29 [0.10, 0.88]
2.13.1 High‐dose single infusion vs medium‐dose single infusion	2	316	Odds Ratio (M‐H, Random, 95% CI)	0.26 [0.01, 4.67]
2.13.2 High‐dose multiple infusion vs medium‐dose single infusion	1	194	Odds Ratio (M‐H, Random, 95% CI)	0.58 [0.19, 1.80]
2.13.3 High‐dose multiple infusion vs medium‐dose multiple infusion	2	530	Odds Ratio (M‐H, Random, 95% CI)	0.24 [0.06, 0.92]
2.13.4 High‐dose multiple infusion vs low‐dose multiple infusion	1	85	Odds Ratio (M‐H, Random, 95% CI)	0.14 [0.01, 2.56]
2.14 Need for additional treatment: higher‐dose single‐infusion regimens vs lower‐dose single‐infusion regimens	2		Odds Ratio (M‐H, Random, 95% CI)	Subtotals only
2.14.1 High‐dose single infusion vs medium‐dose single infusion	2	382	Odds Ratio (M‐H, Random, 95% CI)	0.26 [0.01, 4.80]
2.15 Need for additional treatment: higher‐dose multiple‐infusion regimens vs lower‐dose multiple‐infusion regimens	2	615	Odds Ratio (M‐H, Random, 95% CI)	0.24 [0.08, 0.70]
2.15.1 High‐dose multiple infusion vs medium‐dose multiple infusion	2	504	Odds Ratio (M‐H, Random, 95% CI)	0.23 [0.06, 0.90]
2.15.2 High‐dose multiple infusion vs low‐dose multiple infusion	1	56	Odds Ratio (M‐H, Random, 95% CI)	0.13 [0.01, 2.60]
2.15.3 Medium‐dose multiple infusion vs low‐dose multiple infusion	1	55	Odds Ratio (M‐H, Random, 95% CI)	0.35 [0.03, 3.56]
2.16 Need for additional treatment: single‐infusion regimens vs multiple‐infusion regimens	2	818	Odds Ratio (M‐H, Random, 95% CI)	1.14 [0.38, 3.39]
2.16.1 High‐dose single infusion vs high‐dose multiple infusion	2	818	Odds Ratio (M‐H, Random, 95% CI)	1.14 [0.38, 3.39]
2.17 Length of hospital stay (days): high‐dose regimens vs medium‐ or low‐dose regimens	3	752	Mean Difference (IV, Random, 95% CI)	‐0.24 [‐0.78, 0.30]
2.17.1 High‐dose single infusion vs medium‐dose single infusion	3	558	Mean Difference (IV, Random, 95% CI)	‐0.41 [‐1.15, 0.33]
2.17.2 High‐dose multiple infusion vs medium‐dose single infusion	1	194	Mean Difference (IV, Random, 95% CI)	0.10 [‐0.83, 1.03]
2.18 Length of hospital stay (days): higher‐dose single‐infusion regimens vs lower‐dose single‐infusion regimens	3		Mean Difference (IV, Random, 95% CI)	Subtotals only
2.18.1 High‐dose single infusion vs medium‐dose single infusion	3	621	Mean Difference (IV, Random, 95% CI)	‐0.36 [‐1.09, 0.37]
2.19 Length of hospital stay (days): single‐infusion regimens vs multiple‐infusion regimens	2		Mean Difference (IV, Random, 95% CI)	Subtotals only
2.19.1 High‐dose single infusion vs high‐dose multiple infusion	2	414	Mean Difference (IV, Random, 95% CI)	‐1.24 [‐3.86, 1.37]

**Comparison 3:** Primary treatment ‐ IVIG versus prednisolone

Outcome or subgroup title	No. of studies	No. of participants	Statistical method	Effect size
3.1 CAA: total dose of IVIG vs total dose of IV prednisolone	2	140	Odds Ratio (M‐H, Random, 95% CI)	0.60 [0.24, 1.48]
3.1.1 1200 mg/kg IVIG over 3 days vs 10 mg/kg prednisolone over 5 days	1	90	Odds Ratio (M‐H, Random, 95% CI)	0.49 [0.17, 1.43]
3.1.2 2 g/kg IVIG over 2 days vs 10 mg/kg prednisolone over 5 days	1	50	Odds Ratio (M‐H, Random, 95% CI)	1.00 [0.18, 5.51]
3.2 Adverse events: total dose of IVIG vs total dose of IV prednisolone	1		Odds Ratio (M‐H, Random, 95% CI)	Subtotals only
3.2.1 1200 mg/kg IVIG over 3 days vs 10 mg/kg prednisolone over 5 days	1	90	Odds Ratio (M‐H, Random, 95% CI)	4.18 [0.19, 89.48]
3.3 Duration of fever (days) : total dose of IVIG vs total dose of IV prednisolone	2	140	Mean Difference (IV, Random, 95% CI)	0.68 [‐0.59, 1.94]
3.3.1 1200 mg/kg IVIG over 3 days vs 10 mg/kg prednisolone over 5 days	1	90	Mean Difference (IV, Random, 95% CI)	1.40 [0.42, 2.38]
3.3.2 2 g/kg IVIG over 2 days vs 10 mg/kg prednisolone over 5 days	1	50	Mean Difference (IV, Random, 95% CI)	0.10 [‐0.39, 0.59]

**Comparison 4:** Secondary treatment ‐ IVIG versus infliximab

Outcome or subgroup title	No. of studies	No. of participants	Statistical method	Effect size
4.1 CAA: total dose of single IVIG vs single infliximab	4	197	Odds Ratio (M‐H, Random, 95% CI)	1.31 [0.46, 3.74]
4.1.1 2 g/kg IVIG vs infliximab 5 mg/kg	3	98	Odds Ratio (M‐H, Random, 95% CI)	0.99 [0.17, 5.63]
4.1.2 2 g/kg IVIG vs infliximab 10 mg/kg	1	99	Odds Ratio (M‐H, Random, 95% CI)	1.76 [0.53, 5.80]
4.2 Adverse effects: total dose of single IVIG vs single infliximab	4	201	Odds Ratio (M‐H, Random, 95% CI)	1.13 [0.35, 3.61]
4.2.1 2 g/kg IVIG vs infliximab 5 mg/kg	3	98	Odds Ratio (M‐H, Random, 95% CI)	0.66 [0.17, 2.61]
4.2.2 2 g/kg IVIG vs infliximab 10 mg/kg	1	103	Odds Ratio (M‐H, Random, 95% CI)	2.58 [1.16, 5.75]
4.3 Need for additional treatment: total dose of single IVIG vs single infliximab	4	199	Odds Ratio (M‐H, Random, 95% CI)	3.99 [1.98, 8.02]
4.3.1 2 g/kg IVIG vs infliximab 5 mg/kg	3	98	Odds Ratio (M‐H, Random, 95% CI)	4.12 [1.39, 12.23]
4.3.2 2 g/kg IVIG vs infliximab 10 mg/kg	1	101	Odds Ratio (M‐H, Random, 95% CI)	3.89 [1.56, 9.70]

**Comparison 5:** Secondary treatment ‐ IVIG versus prednisolone

Outcome or subgroup title	No. of studies	No. of participants	Statistical method	Effect size
5.1 CAA: single IVIG vs multiple prednisolone	2	75	Odds Ratio (M‐H, Random, 95% CI)	0.46 [0.02, 9.18]
5.1.1 Total dose of IVIG 2 g/kg vs prednisolone 45 mg/kg	1	53	Odds Ratio (M‐H, Random, 95% CI)	0.08 [0.00, 1.64]
5.1.2 Total dose of IVIG 2 g/kg vs prednisolone 90 mg/kg	1	22	Odds Ratio (M‐H, Random, 95% CI)	1.69 [0.22, 12.81]
5.2 Adverse effects: IVIG vs multiple prednisolone	1		Odds Ratio (M‐H, Random, 95% CI)	Subtotals only
5.2.1 2 g/kg IVIG vs prednisolone 45 mg/kg	1	80	Odds Ratio (M‐H, Random, 95% CI)	0.08 [0.00, 1.49]

**Comparison 6:** Tertiary treatment ‐ IVIG versus prednisolone

Outcome or subgroup title	No. of studies	No. of participants	Statistical method	Effect size
6.1 CAA: IVIG vs prednisolone	1		Odds Ratio (M‐H, Random, 95% CI)	Subtotals only
6.1.1 Total dose of IVIG 1 g/kg vs 20 mg/kg prednisolone over 3 days	1	17	Odds Ratio (M‐H, Random, 95% CI)	0.48 [0.06, 3.99]
6.2 Duration of fever (days): IVIG vs prednisolone	1		Mean Difference (IV, Random, 95% CI)	Subtotals only
6.2.1 Total dose of 1 g/kg IVIG vs 20 mg/kg prednisolone over 3 days	1	17	Mean Difference (IV, Random, 95% CI)	3.40 [2.73, 4.07]

**Comparison 7:** Additional subgroup analysis: primary treatment ‐ IVIG (and ASA) versus ASA

Outcome or subgroup title	No. of studies	No. of participants	Statistical method	Effect size
7.1 Analysis 1.1 subgrouped by geographic distribution	11	1437	Odds Ratio (M‐H, Random, 95% CI)	0.60 [0.41, 0.87]
7.1.1 Japan	10	1288	Odds Ratio (M‐H, Random, 95% CI)	0.64 [0.43, 0.94]
7.1.2 USA and Canada	1	149	Odds Ratio (M‐H, Random, 95% CI)	0.29 [0.10, 0.84]

**Comparison 8:** Additional subgroup analysis: primary treatment ‐ IVIG versus IVG

Outcome or subgroup title	No. of studies	No. of participants	Statistical method	Effect size
8.1 Analysis 2.1.1 subgrouped by geographical distribution	3	555	Odds Ratio (M‐H, Random, 95% CI)	0.85 [0.55, 1.31]
8.1.1 China	2	446	Odds Ratio (M‐H, Random, 95% CI)	0.90 [0.55, 1.45]
8.1.2 Japan	1	109	Odds Ratio (M‐H, Random, 95% CI)	0.70 [0.26, 1.89]
8.2 Analysis 2.2 subgrouped by geographical distribution	3	621	Odds Ratio (M‐H, Random, 95% CI)	0.88 [0.59, 1.32]
8.2.1 China	2	512	Odds Ratio (M‐H, Random, 95% CI)	0.92 [0.59, 1.45]
8.2.2 Japan	1	109	Odds Ratio (M‐H, Random, 95% CI)	0.70 [0.26, 1.89]
8.3 Analysis 2.4 subgrouped by geographical distribution	4	962	Odds Ratio (M‐H, Random, 95% CI)	0.61 [0.28, 1.34]
8.3.1 China	1	267	Odds Ratio (M‐H, Random, 95% CI)	1.04 [0.53, 2.04]
8.3.2 Japan	1	145	Odds Ratio (M‐H, Random, 95% CI)	0.25 [0.07, 0.92]
8.3.3 USA and Canada	2	550	Odds Ratio (M‐H, Random, 95% CI)	0.61 [0.16, 2.43]

## Characteristics of studies

### Characteristics of included studies [ordered by study ID]

**Table:** Barron 1990

***Study characteristics***		
Methods	Design: multicentre RCT Exclusions postrandomisation: 7 (3 no pre‐infusion ECG, 1 pre‐existing CAA, 3 no ECG at 42 to 49 days) Losses to follow‐up: 51 enrolled, 44 completed Intention‐to‐treat analysis: no Duration of study: January 1987 to April 1988	
Participants	Country: USA and Canada Setting: hospitals (7 centres) Number: 47 (intervention n = 25; comparison n = 22) Age (months, mean (SD)): intervention 38.2 (22.2); comparison 30.1 (19.1) Sex (M/F): intervention 17/8; comparison 9/13 Diagnostic criteria described: yes Inclusion criteria: enrolled within 7 days of onset of KD (1st day of fever) Exclusion criteria: clinical or laboratory evidence of disease known to mimic KD; known heart disease; recurrent KD, known immunological defects; prior treatment with any immunomodulating agent	
Interventions	Primary or secondary KD treatment: primary Intervention: 1 g/kg (5 to 8 hours) single‐dose IVIG Comparison: 400 mg/kg (4 days), once daily Concomitant medications: both groups ASA 80 to 100 mg/kg/day (every 6 hours); reduced to 3 to 5 mg/kg/day after resolution of fever (for minimum of 2 months)	
Outcomes	Incidence of CAA Differences in laboratory values Duration of fever Reduction of clinical symptoms Changes in vital signs noted Cardiac evaluation: ECG of coronary arteries, left ventricular dimension and shortening fraction, pericardial space, mitral or aortic insufficiency. Cardiac segments graded as normal ‐ enlarged or dilated ‐ aneurysmal Laboratory evaluations: included complete blood cell, differential cell counts, PLT count, total serum bilirubin concentration Time points: entry, days 4 to 6, 14 to 21, 42 to 49	
Funding	"Supported by a grant from Baxter Healthcare Corporation, Hyland Division, Glendale, Calif., and the American Red Cross."	
Declaration of interest	Not reported	
Notes	IVIG used was Gammagard (Baxter Healthcare Corp, Hyland Division, Glendale, California); human IgG 3 participants excluded for no ECG at 42 to 49 days, were included in adverse event data.	
***Risk of bias***		
**Bias**	**Authors' judgement**	**Support for judgement**
Random sequence generation (selection bias)	Low risk	Quote " ..randomly assigned in blocks of four to receive one of two dosage regimens of IVIG. Each institution was provided with a randomization schedule, and patients were assigned in sequential order to the proper treatment group."
Allocation concealment (selection bias)	Unclear risk	Institutions received randomisation schedule, but unclear if this was open or not.
Blinding of participants and personnel (performance bias) All outcomes	Unclear risk	No blinding of participants or personnel described. Unclear if this could impact outcomes
Blinding of outcome assessment ‐ cardiac abnormalities (detection bias) All outcomes	Low risk	Quote "Each echocardiogram was recorded on videotape and reviewed by two observers who were unaware of patient identity"
Blinding of outcome assessment ‐ non‐cardiac outcomes (detection bias) All outcomes	High risk	No blinding of outcome assessment described, and the outcome measurement may be influenced by lack of blinding.
Incomplete outcome data (attrition bias) All outcomes	Low risk	No concerns over missing data, all accounted for
Selective reporting (reporting bias)	Unclear risk	All expected outcomes reported (no protocol).
Other bias	Unclear risk	There is an imbalance of genders between groups: M/F 17/8 group 1 and 9/13 group 2 (P = 0.06). Unclear if this is sufficient to affect the results

**Table:** Burns 2008

***Study characteristics***		
Methods	Design: multicentre, prospective RCT Exclusions postrandomisation: none Losses to follow‐up: none Intention‐to‐treat analysis: yes Duration of study: September 2004 to September 2006	
Participants	Country: USA Setting: hospitals (6) Number: 24 (intervention n = 12; comparison n = 12) Age (months, median (range)): intervention 22 (3 to 85); comparison 20 (2.4 to 75) Sex (M/F): intervention 9/3; comparison 8/4 Diagnostic criteria described: yes Inclusion criteria: children with acute KD treated with IVIG within 14 days of fever who had persistent or recrudescent fever 48 hours to 7 days after initial IVIG infusion Exclusion criteria: history of TB, histoplasmosis, or coccidiomycosis, recent TB exposure, a chest radiograph suggestive of pulmonary infection obtained within 1 week of study enrolment, immunisation with BCG in the previous 6 months, steroid or other immunomodulatory treatment in previous 1 week	
Interventions	Primary or secondary KD treatment: secondary Intervention: IVIG 2 g/kg Comparison: infliximab 5 mg/kg Concomitant medications: all participants received an initial IVIG infusion (2 g/kg) prior to study enrolment. Paracetamol (15 mg/ kg orally) and diphenhydramine (1 mg/kg orally or IV) were administered before study drug infusion. ASA was administered orally at 80 to 100 mg/kg/day in 4 doses until the first clinical evaluation at week 1, when the ASA dose was reduced to 3 to 5 mg/kg/day. All other antipyretic or anti‐inflammatory therapies were prohibited.	
Outcomes	Primary: pharmacokinetics and safety/tolerability of a single infliximab infusion (5 mg/kg), AE Secondary: cessation of fever, cytokine and CRP levels, WBC count, serum antibodies to infliximab, complete blood count Time points: laboratory assessments were performed at baseline and 2 h, 24 h, 1 week, 2 weeks, and 4 weeks in all study participants CAA reported as AE Cardiac evaluation: ECG at baseline and 2 to 4 weeks after intervention. "The internal lumen diameters of the left anterior descending and right coronary arteries adjusted for body surface and expressed as standard deviation units (Z scores) were recorded. Arteries were classed as normal (Z < 2.5), dilated (Z ≥ 2.5), or aneurysmal (Z ≥ 4.0) with focal dilation of the vessel ≥ 1.5 times the adjacent segment)"	
Funding	Quote "Supported by a grant from Centocor, Inc. and by the National Institute for Child Health and Human Development, Pediatric Pharmacology Research Unit (PPRU) Network (5U10 HD031318). Centocor Inc. performed the determinations of infliximab levels and the assays for anti‐infliximab antibodies. Dr. Gregory F. Keenan and Khalid Mamun participated in editing the manuscript. " GFK and KM employed by Centocor.	
Declaration of interest	Quote "The authors declare no potential conflicts of interest."	
Notes	Pilot study No details on IVIG	
***Risk of bias***		
**Bias**	**Authors' judgement**	**Support for judgement**
Random sequence generation (selection bias)	Low risk	Quote "Subjects were assigned 1:1 to receive intravenous (IV) infliximab 5 mg/kg or IVIG 2 g/ kg using randomly permuted blocks stratified by age"
Allocation concealment (selection bias)	Unclear risk	Insufficient information to permit judgement of low risk or high risk
Blinding of participants and personnel (performance bias) All outcomes	High risk	Participants and personnel were not blinded. "Family members and investigators were aware of the treatment assignment"
Blinding of outcome assessment ‐ cardiac abnormalities (detection bias) All outcomes	High risk	No blinding of outcome assessment described, and the outcome measurement could have been influenced by lack of blinding.
Blinding of outcome assessment ‐ non‐cardiac outcomes (detection bias) All outcomes	High risk	No blinding of outcome assessment described, and the outcome measurement could have been influenced by lack of blinding.
Incomplete outcome data (attrition bias) All outcomes	Low risk	No concerns over missing data, all accounted for
Selective reporting (reporting bias)	Low risk	All expected outcomes reported (NCT00271570).
Other bias	Low risk	The study appears to be free of other sources of bias.

**Table:** Burns 2021

***Study characteristics***		
Methods	Design: multicentre RCT Exclusions postrandomisation: 2 (withdrew consent) Losses to follow‐up: 3 Intention‐to‐treat analysis: yes Duration of study: March 2017 to August 2020	
Participants	Country: USA Setting: 30 hospitals Number: 103 (105 randomised) intervention n = 49; comparison n = 54 Age (years, median (IQR): intervention 2.1 (1.7 to 5.0) ; comparison 3.6 (2.0 to 6.4) Sex (M/F): intervention 30/19; comparison 29/25 Diagnostic criteria described: yes Inclusion criteria: infants and children aged 4 weeks to 17 years who met AHA criteria for complete or incomplete KD and were resistant to initial treatment for KD with IVIG (2 g/kg) within the first 10 days after fever onset were eligible for enrolment Exclusion criteria: initial treatment with IVIG after day 10 of fever and treatment with steroids or other medication for intensification of initial therapy; chronic disease, except asthma, atopic dermatitis, autism, or controlled seizure disorder, known previous infection with TB, coccidioidomycosis, or histoplasmosis, or household contact with active TB; use of TNF α‐blocker within 3 months before enrolment; and patients with a history of hypersensitivity to infliximab	
Interventions	Primary or secondary KD treatment: secondary Intervention: IVIG 2 g/kg over 8 to 12 hours (according to local practice) Comparison: infliximab 10 mg/kg IV over 2 hours Concomitant medications: ASA 30 to 50 mg/kg/day or 80 to 100 mg/kg/day until discharge and then 3 to 5 mg/kg/day until the participant was off study	
Outcomes	Primary outcome: resolution of fever 24 hours after treatment with no recurrence 7 days after discharge Secondary outcomes: duration of fever from enrolment duration of hospitalisation after randomisation decreased inflammation as measured by the change in WBC count, absolute neutrophil count, and CRP concentration from baseline, at 24 hours (plus or minus 2 hours) after completion of the first study treatment, and study completion change of at least 0.5 SD units between baseline and study completion for the right or left anterior descending coronary artery Z score as measured by ECG comparison of therapy‐related AE Cardiac evaluation: participant height, weight, and internal dimension of the right coronary artery, left anterior descending coronary artery, and circumflex artery were recorded at baseline and at study completion. Z scores (internal diameter of the right and left anterior descending normalised for body surface area and expressed as SD units from the mean) were calculated by the Data Coordinating Center in the REDCap database. Z‐worst was defined as the highest Z score for the right coronary artery, left anterior descending coronary artery, or circumflex artery from the echocardiograms at baseline and at study completion.	
Funding	Quote "The study sponsor, Patient‐Centered Outcomes Research Institute, had no role in study design, data collection, data analysis, data interpretation, or writing of the report"	
Declaration of interest	Quote "We declare no competing interests"	
Notes	Quote "...all infliximab or IVIG products were permitted and the manufacturer’s name was recorded" Not specifically reported in article	
***Risk of bias***		
**Bias**	**Authors' judgement**	**Support for judgement**
Random sequence generation (selection bias)	Low risk	Quote "Patients were randomly assigned in a 1:1 ratio to infliximab or second IVIG according to a prespecified randomisation scheme stratified by site, sex (male vs female), and age (>12 months or ≤12 months) by using a randomly permuted block randomisation design with block size of two or four, which was created by the Data Coordinating Center."
Allocation concealment (selection bias)	Low risk	Quote "Patient enrolment and randomisation through the REDCap database was done by the study site investigators who could not predict the next treatment assignment."
Blinding of participants and personnel (performance bias) All outcomes	High risk	Open‐label study, no blinding was undertaken
Blinding of outcome assessment ‐ cardiac abnormalities (detection bias) All outcomes	Unclear risk	There was no blinding of outcome assessment. Steps were taken to standardise ECG imaging across sites, and Z‐scores were calculated by the Data Coordinating Centre in the REDCap database. Quote "...a single echocardiogram of a non‐study patient with KD was submitted by every site and the technical quality assessed by the Director of Non‐Invasive Imaging (BFP) at Rady Children’s Hospital San Diego (San Diego, CA, USA). A written assessment of the ECG quality with suggestions for improvement as needed was sent to every site. ... Z scores (internal diameter of the right and left anterior descending normalised for body surface area and expressed as SD units from the mean) were calculated by the Data Coordinating Center in the REDCap database"
Blinding of outcome assessment ‐ non‐cardiac outcomes (detection bias) All outcomes	High risk	There was no blinding of outcome assessment, and the outcome measurement could have been influenced by lack of blinding.
Incomplete outcome data (attrition bias) All outcomes	Low risk	No concerns over missing data, all accounted for
Selective reporting (reporting bias)	Low risk	All expected outcomes reported (NCT03065244).
Other bias	Low risk	The study appears to be free of other sources of bias.

**Table:** Furusho 1984

***Study characteristics***		
Methods	Design: multicentre RCT Exclusions postrandomisation: 93 randomised (8 not managed by protocol and excluded from study analysis: 4 participants were given IVGG at doses outside the range of 400 ± 50 mg/kg daily, in 1 case IVGG was discontinued because of a chill, 1 case retreated with IVGG during the study period, 1 participant was removed from the trial because of a hepatic disorder attributed to ASA, and 1 participant in the ASA group was treated with IVGG because of worsening symptoms) Losses to follow‐up: none Intention‐to‐treat analysis: no Duration of study: April 1983 to April 1984	
Participants	Country: Japan Setting: 14 hospitals Number: 85 (intervention n = 40; comparison n = 45) Age (months, mean (SD)): intervention 27 (20); comparison 22 (16) Sex (M/F): intervention 24/16; comparison 25/20 Diagnostic criteria described: yes Inclusion criteria: treated for KD within 7 days of onset Exclusion criteria: recurrent or incomplete KD, if treatment could not begin within 7 days of the onset of KD and those with a CAL on admission	
Interventions	Primary or secondary KD treatment: primary Intervention: IVGG plus ASA (IVIG 400 mg/kg daily, for 5 days from day 1 of treatment; ASA 30 to 50 mg/kg daily in 3 divided doses, reduced to 10 to 30 mg/kg once a day once fever gone) Comparison: ASA (30 to 50 mg/kg daily in 3 divided doses, reduced to 10 to 30 mg/kg once a day when fever gone) Concomitant medications: none reported	
Outcomes	CAL (up to 60 days) Fever Laboratory findings (CRP, ESR, PLT count, serum IgG, side effects) Cardiac evaluation: 2‐dimensional ECG 3/week for 60 days after KD onset The development of even a transient dilation of the coronary artery was regarded as indicating a CAL. ECG criteria were those of the KDRC (increase in coronary lumen diameter of 1.5 times or more as compared with adjacent vessel diameter or coronary arteries with diameters of 3 mm or more). A positive report was followed by selective coronary arteriography to confirm the presence of a lesion. Time points: 3 times per week to 60 days for CAA, weeks 1, 2, 3, and 4 for remaining outcomes	
Funding	None reported. Quote "We thank Chemo‐Sero‐Therapeutic Research Institute and TelJin Ltd for donating S‐sulphonated gammaglobulin (Venilon)"	
Declaration of interest	Not reported	
Notes	IVIG: S‐sulphonated intact gammaglobulin; Venilon, Teijin Ltd, Japan This is study 1 of 3 also reported in Furusho 1991.	
***Risk of bias***		
**Bias**	**Authors' judgement**	**Support for judgement**
Random sequence generation (selection bias)	Unclear risk	Eligible participants were randomly allocated to treatment, but details are insufficient to permit a judgement of low risk or high risk.
Allocation concealment (selection bias)	Unclear risk	Insufficient information to permit judgement of low risk or high risk
Blinding of participants and personnel (performance bias) All outcomes	Unclear risk	Blinding is not described. Insufficient information to permit judgement of low risk or high risk
Blinding of outcome assessment ‐ cardiac abnormalities (detection bias) All outcomes	High risk	No blinding of outcome assessment described, and the outcome measurement is likely to be influenced by lack of blinding.
Blinding of outcome assessment ‐ non‐cardiac outcomes (detection bias) All outcomes	High risk	No blinding of outcome assessment described, and the outcome measurement could have been influenced by lack of blinding.
Incomplete outcome data (attrition bias) All outcomes	Low risk	No concerns over missing data. Exclusions postrandomisation explained.
Selective reporting (reporting bias)	Unclear risk	All expected outcomes reported (no protocol).
Other bias	Low risk	The study appears to be free of other sources of bias.

**Table:** Furusho 1991A

***Study characteristics***		
Methods	Design: multicentre prospective RCT Exclusions postrandomisation: 20 (not managed according to the trial protocol) Losses to follow‐up: none Intention‐to‐treat analysis: no Duration of study: May 1984 to September 1985	
Participants	Country: Japan Setting: 16 hospitals Number: 145 (IVGG100 n = 41; IVGG200 n = 51; IVGG400 n = 53) Age (months, mean (SD)): IVGG100 23.7 (23.3); IVGG200 25.7 (21.6); IVGG400 29.7 (23.7) Sex (M/F): IVGG100 21/20; IVGG200 30/21; IVGG400 36/17 Diagnostic criteria described: yes Inclusion criteria: treated for KD within 7 days of onset Exclusion criteria: recurrent or incomplete KD, if treatment could not begin within 7 days of the onset of KD, and those with a CAL on admission	
Interventions	Primary or secondary KD treatment: primary Interventions: IVGG100: IVIG 100 mg/kg/day for 5 days from day 1 of treatment (5% solution 50 drops/min) IVGG200: IVIG 200 mg/kg/day for 5 days from day 1 of treatment (5% solution 50 drops/min) IVGG400: IVIG 400 mg/kg/day for 5 days from day 1 of treatment (5% solution 50 drops/min) Concomitant medication: ASA 30 to 50 mg/kg in 3 doses whilst fever present. After fever 10 to 30 mg/kg/day	
Outcomes	CAL (up to 60 days) Fever Laboratory findings (CRP, ESR, PLT count, serum IgG, adverse effects) Cardiac evaluation: 2‐dimensional ECG 3/week for 60 days after KD onset. The development of even a transient dilation of the coronary artery was regarded as indicating a CAL. ECG criteria were those of the JKDRC (increase in coronary lumen diameter of 1.5 times or more as compared with adjacent vessel diameter or coronary arteries with diameters of 3 mm or more). A positive report was followed by selective coronary arteriography to confirm the presence of a lesion. Time points: 3 times per week to 60 days for CAA, weeks 1, 2, 3, and 4 for remaining outcomes	
Funding	None	
Declaration of interest	Not reported	
Notes	IVIG details: S‐sulphonated intact γ‐globulin (Venilon, Teijin Ltd, Japan) This is study 2 of 3 reported in Furusho 1991.	
***Risk of bias***		
**Bias**	**Authors' judgement**	**Support for judgement**
Random sequence generation (selection bias)	Unclear risk	Quote "Eligible patients were allocated at random to three treatment groups." Insufficient information to permit judgement of low risk of high risk
Allocation concealment (selection bias)	Unclear risk	Quote "Eligible patients were allocated at random to three treatment groups." Insufficient information to permit judgement of low risk of high risk
Blinding of participants and personnel (performance bias) All outcomes	Unclear risk	Blinding is not described. Insufficient information to permit judgement of low risk of high risk
Blinding of outcome assessment ‐ cardiac abnormalities (detection bias) All outcomes	High risk	No blinding of outcome assessment described, and the outcome measurement is likely to be influenced by lack of blinding.
Blinding of outcome assessment ‐ non‐cardiac outcomes (detection bias) All outcomes	High risk	No blinding of outcome assessment described, and the outcome measurement could have been influenced by lack of blinding.
Incomplete outcome data (attrition bias) All outcomes	Unclear risk	No ITT analysis, 20/145 exclusions after randomisation
Selective reporting (reporting bias)	High risk	No description of outcomes or measurements in the methods section. Insufficient information (no protocol)
Other bias	Low risk	The study appears to be free of other sources of bias.

**Table:** Furusho 1991B

***Study characteristics***		
Methods	Design: multicentre, prospective RCT Exclusions postrandomisation: 12 (not managed according to the trial protocol) Losses to follow‐up: none Intention‐to‐treat analysis: no Duration of study: October 1985 to June 1986	
Participants	Country: Japan Setting: 16 hospitals Number: 151 (Group ASA + IVGG n = 49; Group IVGG n = 53; Group ASA n = 49) Age (months, mean (SD)): Group ASA + IVGG 24.3 (21.2); Group IVGG 24.1 (20.0); Group ASA 21.9 (15.1) Sex (M/F): Group ASA + IVGG 28/21; Group IVGG 27/26; Group ASA 26/23 Diagnostic criteria described: yes Inclusion criteria: treated for KD within 7 days of onset Exclusion criteria: recurrent or incomplete KD, if treatment could not begin within 7 days of the onset of KD, and those with a CAL on admission	
Interventions	Primary or secondary KD treatment: primary Interventions: Group ASA + IVGG: IVIG 200 mg/kg/day for 5 days from day 1 of treatment (5% solution 50 drops/min) plus ASA 30 to 50 mg/kg in 3 doses whilst fever present. After fever 10 to 30 mg/kg/day Group IVGG: IVIG 200 mg/kg/day for 5 days from day 1 of treatment (5% solution 50 drops/min). Without ASA Group ASA: ASA 30 to 50 mg/kg in 3 doses whilst fever present. After fever 10 to 30 mg/kg/day Concomitant medications: none stated	
Outcomes	CAL (up to 60 days) Adverse effects Cardiac evaluation: 2‐dimensional ECG 3/week for 60 days after KD onset. The development of even a transient dilation of the coronary artery was regarded as indicating a CAL. ECG criteria were those of the JKDRC (increase in coronary lumen diameter of 1.5 times or more as compared with adjacent vessel diameter or coronary arteries with diameters of 3 mm or more). A positive report was followed by selective coronary arteriography to confirm the presence of a lesion. Time points: 3 times per week to 60 days for CAA, then to 24 months if CAA detected. Remaining outcomes unclear	
Funding	None	
Declaration of interest	Not reported	
Notes	IVIG details: S‐sulphonated intact γ‐globulin (Venilon, Teijin Ltd, Japan) This is study 3 of 3 reported in Furusho 1991.	
***Risk of bias***		
**Bias**	**Authors' judgement**	**Support for judgement**
Random sequence generation (selection bias)	Unclear risk	Quote "Eligible patients were allocated at random to three treatment groups." Insufficient information to permit judgement of low risk or high risk
Allocation concealment (selection bias)	Unclear risk	Quote "Eligible patients were allocated at random to three treatment groups." Insufficient information to permit judgement of low risk or high risk
Blinding of participants and personnel (performance bias) All outcomes	Unclear risk	Blinding is not described. Insufficient information to permit judgement of low risk or high risk
Blinding of outcome assessment ‐ cardiac abnormalities (detection bias) All outcomes	High risk	No blinding of outcome assessment described, and the outcome measurement is likely to be influenced by lack of blinding.
Blinding of outcome assessment ‐ non‐cardiac outcomes (detection bias) All outcomes	High risk	No blinding of outcome assessment described, and the outcome measurement could have been influenced by lack of blinding.
Incomplete outcome data (attrition bias) All outcomes	Unclear risk	No ITT analysis, 12/151 excluded postrandomisation
Selective reporting (reporting bias)	High risk	No description of outcomes or measurements in the methods section. Insufficient information (no protocol)
Other bias	Low risk	The study appears to be free of other sources of bias.

**Table:** Harada 1989

***Study characteristics***		
Methods	Design: multicentre RCT Exclusions postrandomisation: none Losses to follow‐up: 6 (intervention n = 4; comparison n = 2) Intention‐to‐treat analysis: no Duration of study: February 1987 to March 1988	
Participants	Country: Japan Setting: 15 hospitals Number: 242 (intervention n = 125; comparison n = 117) Age (months, mean (SD)): intervention 22.4 (15.0); comparison 19.8 (13.7) Sex (M/F): intervention 76/49; comparison 57/60 Diagnostic criteria described: yes Inclusion criteria: satisfied diagnostic criteria for KD, < 7 days onset, ≤ 4 years old Exclusion criteria: recurrent or incomplete KD, use of steroid or indometacin before treatment	
Interventions	Primary or secondary KD treatment: primary Intervention: IVGG (100 mg/kg/day for 5 consecutive days) plus ASA (50 mg/kg/day) Comparison: IVGG (400 mg/kg/day for 5 consecutive days) plus ASA (50 mg/kg/day) Concomitant medications: ASA (50 mg/kg/day)	
Outcomes	CAL (enrolment, 30 days, 60 days after KD onset, and at 1 year if present at 60 days) Laboratory test (enrolment ‐ 60 days) Cardiac evaluation: 2‐dimensional ECG at enrolment, 1/week from before IVIG treatment for 60 days after KD onset. Echo results were recorded on video and judged by independent staff. Coronary artery diameter of 3 to 5 mm were considered dilations, and 6 mm or greater were considered aneurysms.	
Funding	Quote "I gratefully acknowledge the support of the Kawasaki Disease Research Committee of Japan for this study" (from Harada 1991)	
Declaration of interest	None reported.	
Notes	IVIG details: full molecular type immunoglobulin This study is the same as study 3 (of 3) reported in Harada 1991.	
***Risk of bias***		
**Bias**	**Authors' judgement**	**Support for judgement**
Random sequence generation (selection bias)	Low risk	States central randomisation. Groups were assigned under the direction of an independent controller.
Allocation concealment (selection bias)	Low risk	States central randomisation. Groups were assigned under the direction of an independent controller.
Blinding of participants and personnel (performance bias) All outcomes	Unclear risk	No blinding of participants and personnel described.
Blinding of outcome assessment ‐ cardiac abnormalities (detection bias) All outcomes	Low risk	Harada 1991 reports that ECGs were recorded (stop motion/video) and interpreted by independent observers.
Blinding of outcome assessment ‐ non‐cardiac outcomes (detection bias) All outcomes	High risk	No description of blinding for assessing any other outcome. Unclear if this could have affected other outcome reporting
Incomplete outcome data (attrition bias) All outcomes	Low risk	No concerns over missing data
Selective reporting (reporting bias)	Unclear risk	Expected outcomes reported, but no protocol.
Other bias	Low risk	The study appears to be free of other sources of bias.

**Table:** Hashino 2001

***Study characteristics***		
Methods	Design: RCT Exclusions postrandomisation: none Losses to follow‐up: none Intention‐to‐treat analysis: yes Duration of study: January 1993 to December 1998	
Participants	Country: Japan Setting: hospital Number: 17 (intervention n = 8; comparison n = 9) Age: between 2 months and 10.6 years (no information for groups) Sex (M/F): no information. Quote "no significant difference in gender, age or duration of illness between the patients in these two groups" Diagnostic criteria described: yes Inclusion criteria: diagnosis of KD according to JKDRC criteria, non‐responders to initial and secondary treatment Exclusion criteria: responders to initial or secondary treatment	
Interventions	Primary or secondary KD treatment: tertiary (non‐responders to initial and second IVIG treatment) Intervention: IVIG 1 g/kg Comparison: IV methylprednisolone 20 mg/kg/day for 3 days Concomitant medications: initial treatment was IVIG 2 g/kg; second treatment was IVIG 1 g/kg. All participants were given ASA 30 mg/kg per day.	
Outcomes	CAA Duration of fever Laboratory tests (WBC count, haematocrit, haemoglobin, PLT, CRP, and albumin) Costs of hospitalisation Cardiac evaluation: 2‐dimensional ECG within 24 hours of hospitalisation, then every 24 hours during retreatment and every 7 days after. Change in coronary artery diameter calculated (% change = (diameter after retreatment − diameter before retreatment)/diameter before retreatment X 100) Time points: 24, 48 hours, 7, 14, 21, 28, 35 days	
Funding	Quote "This study was supported, in part, by Grants‐in‐Aid 10470183 and 09770586 from the Ministry of Education, Science and Culture, Japan."	
Declaration of interest	Not reported	
Notes	No details on IVIG manufacturer No specific baseline details provided, but states "no significant difference in gender, age or duration of illness between the patients in these two groups".	
***Risk of bias***		
**Bias**	**Authors' judgement**	**Support for judgement**
Random sequence generation (selection bias)	Unclear risk	Quote "We randomly divided these 17 patients into two groups". Insufficient information to permit judgement of low risk or high risk
Allocation concealment (selection bias)	Unclear risk	Insufficient information to permit judgement of low risk or high risk
Blinding of participants and personnel (performance bias) All outcomes	High risk	No blinding of participants or personnel
Blinding of outcome assessment ‐ cardiac abnormalities (detection bias) All outcomes	Unclear risk	No blinding of outcome assessors, but internal checks on reproducibility on coronary images carried out with little variance. Quote "Two independent observers (KH, MI), with each observer individually selecting the frames to measure and having no knowledge of the results obtained by the other observer, analyzed 20 randomly selected patients at different times."
Blinding of outcome assessment ‐ non‐cardiac outcomes (detection bias) All outcomes	High risk	No blinding of outcome assessment described, and the outcome measurement could have been influenced by lack of blinding.
Incomplete outcome data (attrition bias) All outcomes	Low risk	No concerns over missing data, all accounted for
Selective reporting (reporting bias)	Low risk	All expected outcomes reported (protocol available).
Other bias	Low risk	The study appears to be free of other sources of bias.

**Table:** He 2021

***Study characteristics***		
Methods	Design: open‐label, multicentre, prospective RCT Exclusions postrandomisation: none Losses to follow‐up: 7 (Group A 4; Group B 2; Group C 1) Intention‐to‐treat analysis: no (states primary outcome was ITT) Duration of study: January 2014 to December 2015	
Participants	Country: China Setting: 3 hospitals (Children’s Hospital of Fudan University, Shanghai Children’s Medical Center, and Shanghai Children’s Hospital) Number: 397 (404 randomised) (Group A 138; Group B 127; Group C 132) Age (months, mean ± SD): Group A 26.4 ± 22.7; Group B 28.8 ± 22.3; Group C 26.1 ± 20.0 Sex (M/F): Group A 90/48; Group B 82/45; Group C 77/55 Diagnostic criteria described: yes Inclusion criteria: within 5 to 10 days of KD onset (the first day of illness defined as the first day of fever), age ≤ 1 month at onset to < 12 years of age Exclusion criteria: afebrile before enrolment, previous history of KD; CAL before enrolment; history of administration of steroids (oral, intravenous, intramuscular, or subcutaneous) in the previous 30 days or administration of IVIG in the previous 180 days, severe concomitant medical disorders (chromosomal abnormality, immunodeficiency, complex congenital heart disease, metabolic disorders, nephritis, collagen diseases, etc.), suspected infectious diseases including sepsis, septic meningitis, peritonitis, bacterial pneumonia, varicella, and influenza	
Interventions	Primary or secondary KD treatment: primary Intervention Group A: IVIG 2 g/kg once over 12 to 24 hours Intervention Group B: IVIG 1 g/kg twice, over 2 days Intervention Group C: IVIG 1 g/kg once over 12 to 24 hours Concomitant medications: ASA at 30 to 50 mg/kg/day split into 3 doses per day, reduced to 3 to 5 mg/kg per day when fever subsided for 3 days and CRP ≤ 8 mg/L Participants admitted before day 5 of illness were treated with ASA only and began IVIG treatment between day 5 and 10 of illness.	
Outcomes	Primary outcomes: Hours to defervescence Incidence of CAL Secondary outcomes: IVIG resistance, total fever days, total dose of IVIG, changes in laboratory data 72 hours after completion of initial IVIG infusion, incidence of CAL during the whole study period, length of hospital stay, hospitalisation expenses, and serious adverse events (including death, severe infection, heart failure, allergic reactions) Cardiac evaluation: 2‐dimensional ECG before treatment and approximately 2 weeks, 1, 3, 6 months after onset. Measurement included the diameter of the left main coronary artery, left anterior descending artery, left circumflex coronary artery and proximal, middle and distal segment of right coronary artery. CAL defined as dilation or aneurysm, when luminal diameter was > 3.0 mm in children < 5 years or > 4.0 mm for ≥ 5 years, or when internal diameter of a segment was ≥ 1.5 times adjacent segment, or Z‐score > 2.5. Medium aneurysm defined as an internal luminal diameter from 4 to 8 mm; giant aneurysm defined as an internal luminal diameter > 8.0 mm. CAL severity classified as: level 1: no changes at any stage of illness; level 2: transient coronary ectasia (disappears within 1 month of onset); level 3: isolated small to medium (> 4.0 mm but ≤ 8.0 mm, Z‐score 2.5 to 10), CAA in ≥ 1 coronary arteries; level 4: ≥ 1 large or giant CAA (≥ 8.0 mm or Z‐score ≥ 10), or multiple aneurysms in same artery without obstruction; level 5: coronary artery obstruction. Time points: CAA 2 weeks, 1, 3, and 6 months	
Funding	Quote "This study was funded by Joint Research Projects of Emerging Cutting‐edge Technologies of Shanghai Municipal Hospitals (SHDC12013106), Science and Technology Commission of Shanghai Municipality Projects (15ZR1404300), and Chinese Academy of Medical Sciences Research Unit (2018RU002)"	
Declaration of interest	Quote "The authors declare no conflict of interest."	
Notes	2 IVIG products used ‐ both pH4 and manufactured in Shanghai. Participants included 13 incomplete KD patients (9.42%).	
***Risk of bias***		
**Bias**	**Authors' judgement**	**Support for judgement**
Random sequence generation (selection bias)	Low risk	Quote "Randomization was performed using a simple randomization sequence generated by Microsoft Excel. A set of instructions about correct implementation of the randomized trial was sent to the three participating hospitals."
Allocation concealment (selection bias)	Unclear risk	Hospitals had instructions on the correct implementation of randomisation, but insufficient information to permit judgement of low risk or high risk.
Blinding of participants and personnel (performance bias) All outcomes	High risk	Quote "Patients and physicians were not masked to the group assignments, while echocardiologists assessing CAL were blinded."
Blinding of outcome assessment ‐ cardiac abnormalities (detection bias) All outcomes	Low risk	Quote "Patients and physicians were not masked to the group assignments, while echocardiologists assessing CAL were blinded."
Blinding of outcome assessment ‐ non‐cardiac outcomes (detection bias) All outcomes	High risk	No blinding described for other outcomes. Quote "Patients and physicians were not masked to the group assignments, while echocardiologists assessing CAL were blinded."
Incomplete outcome data (attrition bias) All outcomes	Low risk	No concerns over missing data
Selective reporting (reporting bias)	Low risk	All expected outcomes reported (NCT02439996).
Other bias	Low risk	The study appears to be free of other sources of bias.

**Table:** Matsushima 1985

***Study characteristics***		
Methods	Design: multicentre RCT Exclusions postrandomisation: none Losses to follow‐up: none Intention‐to‐treat analysis: no Duration of study: September 1983 to July 1984	
Participants	Country: Japan Setting: 15 hospitals Number: 34 (intervention n = 17; comparison n = 17) Age (months, mean/SD): intervention 26.1 (16.5); comparison 34.1 (25.1) Sex (M/F): intervention 9/8; comparison 8/9 Diagnostic criteria described: yes Inclusion criteria: treated for KD within 10 days of onset Exclusion criteria: recurrent or incomplete KD	
Interventions	Primary or secondary KD treatment: primary Intervention: IVIG (400 mg/kg/day for 5 consecutive days) plus ASA (30 mg/kg/day) Comparison: ASA (30 mg/kg/day) Concomitant medications: none	
Outcomes	Fever, laboratory findings (WBC, CRP, ESR, PLT count, serum IgG, albumin score), complement and cellular immunity testing (IgA, IgM, IgE, C3, C4, CH50, T4/T8) Cardiac evaluation: 2‐dimensional ECG 1/week for 30 days after KD onset; coronary artery diameter of 3 to 5 mm were considered dilations, and 6 mm or greater were considered aneurysms Time points: CAA 1, 2, 3, 4 weeks	
Funding	None reported.	
Declaration of interest	None reported.	
Notes	IVIG details: polyethylene glycol‐treated human normal immunoglobulin (Venoglobulin I) Additional data provided by study authors (unable to provide data on length of hospital stay).	
***Risk of bias***		
**Bias**	**Authors' judgement**	**Support for judgement**
Random sequence generation (selection bias)	High risk	States central randomisation, but also that interventions were assigned based on even or odd registration numbers ‐ this suggests not true randomisation
Allocation concealment (selection bias)	High risk	Assigned based on even or odd registration numbers
Blinding of participants and personnel (performance bias) All outcomes	Unclear risk	No blinding of participants or personnel described.
Blinding of outcome assessment ‐ cardiac abnormalities (detection bias) All outcomes	High risk	No blinding of outcome assessment, and the outcome measurement could have been influenced by lack of blinding
Blinding of outcome assessment ‐ non‐cardiac outcomes (detection bias) All outcomes	High risk	No blinding of outcome assessment, and the outcome measurement could have been influenced by lack of blinding
Incomplete outcome data (attrition bias) All outcomes	Low risk	No concerns over missing data
Selective reporting (reporting bias)	Unclear risk	Expected outcomes reported, but no protocol.
Other bias	Low risk	The study appears to be free of other sources of bias.

**Table:** Miura 2008

***Study characteristics***		
Methods	Design: RCT Exclusions postrandomisation: none Losses to follow‐up: none Intention‐to‐treat analysis: no Duration of study: January 2001 to May 2003	
Participants	Country: Japan Setting: hospital Number: 22 (intervention n = 11; comparison n = 11) Age (months, mean (SD)): intervention 31 (26); comparison 32 (19) Sex (M/F): intervention 5/3; comparison 5/2 Diagnostic criteria described: no (states number of clinical signs in the diagnostic criteria for KD in terms of patients with 5 signs compared to those with 6 signs was 5:2 vs 6:2) Inclusion criteria: patients with acute KD unresponsive to initial IVIG therapy (persistent or recurrent fever ≥ 37.5 ˚C 48 hours after single infusion 2 g/kg IVIG) Exclusion criteria: not described	
Interventions	Primary or secondary KD treatment: secondary Intervention: IVIG 2 g/kg over 24 hours Comparison: IVMP 30 mg/kg/day for 3 days; heparin continuously infused 15 to 20 units/kg/h Concomitant medications: none stated	
Outcomes	Primary outcomes: cytokine levels Secondary outcomes: fever CAL AE Cardiac evaluation: internal diameters of any coronary arteries, their Z scores (no further details) Time points: enrolment, daily to day 7	
Funding	Not reported	
Declaration of interest	Quote "Competing interests: none declared"	
Notes	IVIG: polyethylene glycol‐treated human immunoglobulin (Venoglobulin IH; Mitsubishi Pharma Corp, Osaka, Japan) The study was halted prematurely when 22 participants were recruited because of adverse effects of IVMP. Number of participants differs between the 2 reports (22 vs 15). We contacted the study authors, and they confirmed that the same participants were involved, but cytokines could only be measured in 15.	
***Risk of bias***		
**Bias**	**Authors' judgement**	**Support for judgement**
Random sequence generation (selection bias)	Unclear risk	Described as randomised, but no details provided Quote "At enrolment, the subjects were randomly assigned to either the IVMP group, 30 mg/kg of methyl‐ prednisolone per day for 3 consecutive days, or the additional IVIG group, 2 g/kg over 24 hours"
Allocation concealment (selection bias)	Unclear risk	Insufficient information to permit judgement of low risk or high risk
Blinding of participants and personnel (performance bias) All outcomes	High risk	Quote "Treating physicians, echocardiographers, or patients’ families were not blinded, raising the possibility of a bias in interpreting the results."
Blinding of outcome assessment ‐ cardiac abnormalities (detection bias) All outcomes	High risk	Quote "Treating physicians, echocardiographers, or patients’ families were not blinded, raising the possibility of a bias in interpreting the results."
Blinding of outcome assessment ‐ non‐cardiac outcomes (detection bias) All outcomes	High risk	Quote "Treating physicians, echocardiographers, or patients’ families were not blinded, raising the possibility of a bias in interpreting the results."
Incomplete outcome data (attrition bias) All outcomes	Low risk	Number of participants differs between the 2 reports (22 vs 15), but this was explained by study authors (personal communication).
Selective reporting (reporting bias)	Unclear risk	All expected outcomes reported, but no protocol available.
Other bias	Unclear risk	The study was halted prematurely when 22 participants were recruited because of adverse effects of IVMP.

**Table:** Mori 2017

***Study characteristics***		
Methods	Design: multicentre RCT Exclusions postrandomisation: none Losses to follow‐up: 14 (worsening KD) Intention‐to‐treat analysis: no Duration of study: May 2012 to September 2014	
Participants	Country: Japan Setting: 10 medical institutions Number: 31 (intervention n = 15; comparison n = 16) Age (years, median (range)): intervention 2.5 (1 to 6); comparison 3.0 (1 to 4) Sex (M/F): intervention 11/4; comparison 10/6 Diagnostic criteria described: yes Inclusion criteria: patients diagnosed with KD aged 1 to 10 years; refractory to initial IVIG therapy (2 g/kg), defined as persistent fever of ≥ 38.0 ˚C (axillary temperature) and ≥ 4 h within 24 to 36 h after completion of the initial IVIG infusion and increases in either WBC count, neutrophil count, or CRP within 24 to 36 h since before initial IVIG administration, fever of ≥ 37.5 ˚C at enrolment, could be administered study drug within 8 days of illness onset (the first day) Exclusion criteria: patients with CAA before enrolment, BCG vaccination within 6 months before enrolment or no BCG vaccination; treatments other than initial IVIG for KD; corticosteroids (prednisolone equivalent of ≥ 1 mg/kg/day) within 4 weeks before enrolment; history of infliximab and other biologics; abnormal laboratory results after consent; complication or a history of infections; history of hypersensitivity to IVIG; immunodeficiency; or serious complications requiring hospitalisation	
Interventions	Primary or secondary KD treatment: secondary Intervention: IVIG single dose 2 g/kg over 20 hours Comparison: infliximab single dose of 5 mg/kg over 2 hours Concomitant medications: ASA 30 to 50 mg/kg/day at acute stage and 3 to 5 mg/kg/day after defervescence	
Outcomes	Primary outcome: defervescence rate within 48 hours after the start of treatment Secondary outcomes: overall defervescence rate until 48 hours duration of fever incidence of CALs on days 21 and 56 Z‐score until day 56 presence/absence of major symptoms (bilateral bulbar conjunctival congestion, lip/oral cavity changes, polymorphous rash, distal extremity changes, and non‐suppurative cervical lymphadenopathy) in the acute stage laboratory variables (WBC count, neutrophil count, PLT count, albumin, and CRP) Cardiac evaluation: CALs were defined as a coronary artery with an internal diameter of ≥ 3 mm in participants aged < 5 years or ≥ 4 mm in participants aged ≥ 5 years. Z‐scores were determined using the lambda‐mu‐sigma method.	
Funding	Quote "Mitsubishi Tanabe Pharma Corporation sponsored this clinical trial and was responsible for the collection and analysis of data. The sponsor was involved with the study design and interpretation of data."	
Declaration of interest	Quote "Competing Interests: M.M. received personal fees from Mitsubishi Tanabe Pharma Corporation during the conduct of the trial and grants from Chugai Pharmaceutical Co., Ltd.; Ono Pharmaceutical Co., Ltd.; Mitsubishi Tanabe Pharma Corporation; UCB Japan Co., Ltd.; Towa Pharmaceutical Co., Ltd.; AbbVie G.K.; Japan Blood Products Organization; Ayumi Pharmaceutical Co.; CSL Behring; and Nippon Kayaku Co., Ltd.; and personal fees from AbbVie G.K.; MSD K.K.; Daiichi Sankyo Co., Ltd.; and Taisho Pharmaceutical Co., Ltd. outside the submitted work. T.H. received grants from Mitsubishi Tanabe Pharma Corporation during the conduct of the trial and personal fees from Mitsubishi Tanabe Pharma Corporation outside the submitted work. M.K. received grants from Mitsubishi Tanabe Pharma Corporation during the conduct of the trial. H.S. received grants from Mitsubishi Tanabe Pharma Corporation, during the conduct of the trial and personal fees from Taisho Toyama Pharmaceutical Co., Ltd. outside the submitted work. T.M. received grants from Mitsubishi Tanabe Pharma Corporation during the conduct of the trial. S.I. received grants from Mitsubishi Tanabe Pharma Corporation during the conduct of the trial. T.O. received grants from Mitsubishi Tanabe Pharma Corporation during the conduct of the trial and personal fees from Mitsubishi Tanabe Pharma Corporation and TEIJIN PHARMA Limited outside the submitted work. K.H. received grants from Mitsubishi Tanabe Pharma Corporation during the conduct of the trial and personal fees from Mitsubishi Tanabe Pharma Corporation outside the submitted work. N.K. received grants from Mitsubishi Tanabe Pharma Corporation during the conduct of the trial and personal fees from Mitsubishi Tanabe Pharma Corporation outside the submitted work. K.W. received grants from Mitsubishi Tanabe Pharma Corporation during the conduct of the trial. Y.S. received grants from Mitsubishi Tanabe Pharma Corporation during the conduct of the trial. Y.O. received grants from Mitsubishi Tanabe Pharma Corporation during the conduct of the trial. H.Y. received personal fees from Mitsubishi Tanabe Pharma Corporation during the conduct of the trial and personal fees from Mitsubishi Tanabe Pharma Corporation outside the submitted work. C.I. received personal fees from Mitsubishi Tanabe Pharma Corporation during the conduct of the trial and personal fees from Mitsubishi Tanabe Pharma Corporation outside the submitted work. T.K. received grants from Mitsubishi Tanabe Pharma Corporation during the conduct of the trial and personal fees from Actelion Pharmaceuticals Japan, Ltd.; Astellas Pharma Inc.; and Meiji Seika Pharma Co., Ltd. outside the submitted work. S.F. received personal fees from Mitsubishi Tanabe Pharma Corporation, Teijin Pharma Ltd., Nihon Pharmaceutical Co., Ltd.; and Japan Blood Products Organization outside the submitted work."	
Notes	Recruitment target was not reached. 5/16 and 9/15 participants receiving infliximab and IVIG discontinued the trial due to worsening KD (persistent fever or CALs development) and were switched to another treatment at physician’s discretion. IVIG: participants received 1 of 4 approved IVIG formulations in Japan as initial IVIG therapy: VGIH (Venoglobulin IH, Japan Blood Products Organization, Tokyo, Japan), freeze‐dried sulphonated human normal Ig (Kenketsu Venilon‐I, Teijin Co Ltd, Tokyo, Japan), freeze‐dried VGIH (Kenketsu Glovenin‐I, Nihon Pharmaceutical Co Ltd, Tokyo, Japan), or pH4‐treated acidic human normal Ig (Nisseki Polyglobin‐N 5%, 10% I.V., Japan Red Cross Society, Tokyo, Japan)	
***Risk of bias***		
**Bias**	**Authors' judgement**	**Support for judgement**
Random sequence generation (selection bias)	Low risk	Quote "Randomization was performed centrally using a dynamic allocation method, with sex and age as assignment factors."
Allocation concealment (selection bias)	Low risk	Quote "Randomization was performed centrally using a dynamic allocation method, with sex and age as assignment factors."
Blinding of participants and personnel (performance bias) All outcomes	High risk	Quote "Because of the difference in their infusion periods, infliximab and VGIH [IVIG] were administered open‐label."
Blinding of outcome assessment ‐ cardiac abnormalities (detection bias) All outcomes	Low risk	Assessors for CAL were blinded. Quote "CALs were assessed using echocardiography by a Central Review Committee, comprising three KD specialists who were blinded to patient information at all time points except baseline."
Blinding of outcome assessment ‐ non‐cardiac outcomes (detection bias) All outcomes	High risk	No blinding of outcome assessment described, and the outcome measurement could have been influenced by lack of blinding.
Incomplete outcome data (attrition bias) All outcomes	Unclear risk	17/31 completed the study (11 in infliximab group, 6 in IVIG group).
Selective reporting (reporting bias)	Low risk	All expected outcomes reported (NCT01596335).
Other bias	Unclear risk	Recruitment target was not reached. 5/16 and 9/15 participants receiving infliximab and IVIG, respectively, discontinued the trial due to worsening KD (persistent fever or CALs development) and were switched to another treatment at physician’s discretion.

**Table:** Morikawa 1994

***Study characteristics***		
Methods	Design: multicentre RCT Exclusions postrandomisation: 15 Losses to follow‐up: 15 Intention‐to‐treat analysis: no Duration of study: September 1991 to January 1993	
Participants	Country: Japan Setting: 95 hospitals Number: 451 (intervention group A n = 147; intervention group B n = 152; intervention group C n = 152) Age, years: 0 to 6 (< 1 year: 50, 52, 51; > 1 < 2 years: 30, 45, 41; > 2 < 3 years: 29, 26, 25; > 3 < 4 years: 19, 19, 18; > 4 < 5 years: 12, 7, 10; > 5 < 6 years: 7, 3, 7; group A, B, C respectively) Sex (M/F): intervention group A 86/61; intervention group B 87/65; intervention group C 87/65 Diagnostic criteria described: yes Inclusion criteria: KD patients enrolled within 9 days of onset, with day 1 defined as the first day of fever, less than 6 years old Exclusion criteria: relapse of KD, CAA before IVIG, IgA deficiency, history of allergic reaction to IVIG, received treatment with IVIG, steroid, or indometacin before referral to the study centre, severe renal failure	
Interventions	Primary or secondary KD treatment: primary Intervention group A: IVIG 200 mg/kg per day for 5 days Intervention group B: IVIG 400 mg/kg per day for 5 days Intervention group C: sulphonated IVIG 200 mg/kg per day for 5 days Concomitant medications: ASA 30 mg/kg per day until the fever subsided to < 37.5 ˚C then at 10 mg/kg per day until the 30th day after onset of the illness or the day of hospital discharge	
Outcomes	Primary outcome: CAA until 30 days Secondary outcomes: duration of fever after administration of IVIG, AE Cardiac evaluation: 2‐dimensional ECG, recorded on a videotape and a photograph. Each coronary artery was categorised as non‐visualised, normal (< 3 mm diameter), dilatation (23 mm and < 5 mm diameter), medium aneurysm (35 mm and < 8 mm diameter), and large aneurysm (28 mm diameter) Time points: fever to 120 hours, CAA and AE to 30 days	
Funding	Not reported	
Declaration of interest	Not reported	
Notes	IVIG Group A and B: polyethyleneglycol‐treated human immunoglobulin GB‐0998 (The Green Cross Corporation, Osaka, Japan) IVIG Group C: freeze‐dried, sulphonated human immunoglobulin (Teijin Co Ltd, Tokyo, Japan) Quote "Each study center, at which the patient was enrolled, had their own independent interpretation of echocardiograms. The disagreements between the center’s interpretation and that of the study committee were reevaluated and resolved by the individual study center, based on the interpretation of the study committee"	
***Risk of bias***		
**Bias**	**Authors' judgement**	**Support for judgement**
Random sequence generation (selection bias)	Low risk	Quote "Patients were randomized to the three treatment groups by a central registration system. Randomization was per formed by the minimization method using age (less than one year vs at least one year), sex and study center as prognostic factors for balancing."
Allocation concealment (selection bias)	Low risk	Quote "Patients were randomized to the three treatment groups by a central registration system."
Blinding of participants and personnel (performance bias) All outcomes	Unclear risk	Blinding of participants and personnel not described. Insufficient information to permit judgement of low risk or high risk
Blinding of outcome assessment ‐ cardiac abnormalities (detection bias) All outcomes	Low risk	Quote "Each patient’s echocardiograms were recorded on a video‐ tape and a photograph. Echocardiograms on photograph were interpreted blindly and independently by two pediatric cardiologists from the Study Committee composed of the authors, under the supervision of the statistician of this study, and videotapes were reviewed repeatedly when necessary."
Blinding of outcome assessment ‐ non‐cardiac outcomes (detection bias) All outcomes	High risk	No blinding of outcome assessment described, and the outcome measurement could have been influenced by lack of blinding.
Incomplete outcome data (attrition bias) All outcomes	Low risk	No concerns over missing data
Selective reporting (reporting bias)	Unclear risk	All expected outcomes reported, but no protocol available.
Other bias	Low risk	The study appears to be free of other sources of bias.

**Table:** Nagashima 1987

***Study characteristics***		
Methods	Design: multicentre RCT Exclusions postrandomisation: none Losses to follow‐up: none Intention‐to‐treat analysis: yes Duration of study: July 1984 to April 1986	
Participants	Country: Japan Setting: 16 hospitals Number 136 (intervention n = 69; comparison n = 67) Age (months, mean (SD)): intervention 25.6 (20.1); comparison 22.7 (14.4) Sex (M/F): intervention 46/23; comparison 41/26 Diagnostic criteria described: no Inclusion criteria: children admitted with KD within 10 days of onset Exclusion criteria: CAL before treatment	
Interventions	Primary or secondary KD treatment: primary Intervention: IVIG 400 mg/kg for 1 to 2 hours for 3 days and ASA 30 mg/kg/day Comparison: ASA 30 mg/kg/day Concomitant medications: none stated	
Outcomes	CAL Laboratory test (WBC, CRP, ESR, total serum protein, protein fractions, IgG, IgM, and IgA) Febrile period AE Cardiac evaluation: 2‐dimensional ECG > 3/week. Coronary arteries 3 to 5 mm were considered dilated; > 6 mm or spherical or spindle shape associated with lumen diameter ≥ 1.5 times diameter of adjacent vessel considered as aneurysms Time points: at enrolment, > 3 ECG per week to 30 days, temperature daily	
Funding	None reported.	
Declaration of interest	Not reported	
Notes	IVIG: polyethylene glycol‐treated intact gammaglobulin (Venoglobulin I, Green Cross Corporation, Japan)	
***Risk of bias***		
**Bias**	**Authors' judgement**	**Support for judgement**
Random sequence generation (selection bias)	High risk	Registered by calling to the controller, with odd registration order being the gammaglobulin group and the even order being the ASA group
Allocation concealment (selection bias)	High risk	Registered by calling to the controller, with odd registration order being the gammaglobulin group and the even order being the ASA group
Blinding of participants and personnel (performance bias) All outcomes	High risk	No blinding of participants. The outcome measurement could have been influenced by lack of blinding.
Blinding of outcome assessment ‐ cardiac abnormalities (detection bias) All outcomes	High risk	No blinding of outcome assessors. The outcome measurement could have been influenced by lack of blinding.
Blinding of outcome assessment ‐ non‐cardiac outcomes (detection bias) All outcomes	High risk	No blinding of outcome assessors. The outcome measurement could have been influenced by lack of blinding.
Incomplete outcome data (attrition bias) All outcomes	Low risk	No concerns over missing data
Selective reporting (reporting bias)	Unclear risk	Expected outcomes reported, but no protocol available.
Other bias	Low risk	The study appears to be free of other sources of bias.

**Table:** Newburger 1986

***Study characteristics***		
Methods	Design: multicentre RCT Exclusions postrandomisation: none Losses to follow‐up: 4 Intention‐to‐treat analysis: no Duration of study: February 1984 to September 1985	
Participants	Country: USA Setting: 6 hospitals Number: 168 (intervention n = 84; comparison n = 84) Age (years, mean (SD)): intervention 2.7 (0.2); comparison 2.3 (0.2) Sex (M/F): intervention 49/35; comparison 50/34 (calculated) Diagnostic criteria described: yes Inclusion criteria: patients met 5 of 6 criteria of KD, enrolled within 10 days of onset, where day 1 was first day of fever Exclusion criteria: clinical or laboratory evidence of any other disease known to mimic KD	
Interventions	Primary or secondary KD treatment: primary Intervention: IVIG 400 mg/kg/day over 2 hours for 4 days; plus ASA 100 mg/kg/day (80 to 120 mg/kg/day) every 6 hours to day 14 of illness, then reduced to 3 to 5 mg/kg/day single dose Comparison: ASA 100 mg/kg/day (80 to 120 mg/kg/day) every 6 hours until day 14 of illness, then reduced to 3 to 5 mg/kg/day single dose Concomitant medications: none	
Outcomes	Primary outcome: CAA Secondary outcomes: Fever Laboratory test (WBC, PLT) AE Cardiac evaluation: 2‐dimensional ECG of the right coronary artery, and left main, anterior descending and circumflex branches of the left coronary artery; at enrolment, 2 and 7 weeks. Defined as abnormal when diameter of the lumen was at least 3 mm or 1.5 times as large as diameter of adjacent vessel Time points: enrolment, 2 and 7 weeks	
Funding	Quote "Supported by grants (HL 34545, RR 2172, RR 69, and RR 827) from the National Institutes of Health"	
Declaration of interest	Not reported	
Notes	IVIG: intact, unmodified, supplied by Immuno AG (Austria) Not all participants in ASA group hospitalised. Only hospitalised participants used in fever analysis to prevent bias from home reporting.	
***Risk of bias***		
**Bias**	**Authors' judgement**	**Support for judgement**
Random sequence generation (selection bias)	Low risk	Quote "The coordinating center developed randomization schemes for each age‐sex group at each clinical center and supplied the centers with four sets of sealed, serially numbered, opaque envelopes containing the subjects' treatment assignments"
Allocation concealment (selection bias)	Low risk	Quote "...four sets of sealed, serially numbered, opaque envelopes containing the subjects' treatment assignments"
Blinding of participants and personnel (performance bias) All outcomes	Unclear risk	Insufficient information to permit judgement of low risk or high risk
Blinding of outcome assessment ‐ cardiac abnormalities (detection bias) All outcomes	Low risk	Echocardiograms recorded and interpreted blindly and independently by 2 paediatric echocardiographers from other centres.
Blinding of outcome assessment ‐ non‐cardiac outcomes (detection bias) All outcomes	High risk	No blinding of outcome assessment described, and the outcome measurement could have been influenced by lack of blinding.
Incomplete outcome data (attrition bias) All outcomes	Low risk	No concerns over missing data
Selective reporting (reporting bias)	Unclear risk	Expected outcomes reported, but no protocol available.
Other bias	Low risk	The study appears to be free of other sources of bias.

**Table:** Newburger 1991

***Study characteristics***		
Methods	Design: multicentre RCT Exclusions postrandomisation: none Losses to follow‐up: 6 Intention‐to‐treat analysis: no Duration of study: May 1986 to November 1989	
Participants	Country: USA Setting: 7 centres Number: 549 (intervention n = 273; comparison n = 276) Age (years, mean (SD)): intervention 2.9 (0.1); comparison 2.9 (0.1) Sex (M/F): intervention 164/109; comparison 164/112 Diagnostic criteria described: yes Inclusion criteria: fever plus 4 of 5 diagnostic criteria, enrolled within 10 days of onset, day 1 defined as first day of fever Exclusion criteria: clinical or laboratory evidence of any disease known to mimic KD, treatment with IVIG before referral	
Interventions	Primary or secondary KD treatment: primary Intervention: IVIG 2 g/kg over 8 to 12 hours (single infusion) Comparison: IVIG 400 mg/kg/day over 2 hours for 4 days Concomitant medications: ASA 80 to 100 mg/kg/day to day 14 then 3 to 5 mg/kg/day	
Outcomes	Primary outcomes: presence or absence of CAA at 2 weeks and 7 weeks maximal temperature at day 3 laboratory indexes of systemic inflammation Secondary outcomes: AE laboratory testing (complete cell count, WBC count, PLT count, IgG, CRP) Cardiac evaluation: 2‐dimensional ECG of the right coronary artery, and left main, anterior descending and circumflex branches of the left coronary artery and posterior descending coronary artery ‐ at enrolment, 2 and 7 weeks. Each coronary artery categorised as non visualised, normal or abnormal (showing ectasia or aneurysm). Defined as abnormal if the lumen diameter (inside to inside) ≥ 3 mm in a child < 5 years of age, or ≥ 4 mm in ≥ 5 years of age; or if the lumen diameter of a segment ≥ 1.5 times that of an adjacent segment; or clearly irregular Time points: enrolment, 2 and 7 weeks	
Funding	Quote "Supported by grants (HL 34545, RR 02172, and RR 69) from the National Institutes of Health"	
Declaration of interest	Not reported	
Notes	IVIG: Immuno AG	
***Risk of bias***		
**Bias**	**Authors' judgement**	**Support for judgement**
Random sequence generation (selection bias)	Low risk	Quote "The coordinating center developed randomization schemes for each age‐and‐sex group at each clinical center and supplied the centers with four sets of sealed, serially numbered, opaque envelopments that contained the patient treatment assignments."
Allocation concealment (selection bias)	Low risk	Quote "...sealed, serially numbered, opaque envelopments that contained the patient' treatment assignments."
Blinding of participants and personnel (performance bias) All outcomes	Unclear risk	Insufficient information to permit judgement of low risk or high risk
Blinding of outcome assessment ‐ cardiac abnormalities (detection bias) All outcomes	Low risk	Quote "Each patient's echocardiograms were recorded on a single videotape in chronologic order and were interpreted blindly and independently by two pediatric echocardiographers."
Blinding of outcome assessment ‐ non‐cardiac outcomes (detection bias) All outcomes	High risk	No blinding of outcome assessment described, and the outcome measurement could have been influenced by lack of blinding.
Incomplete outcome data (attrition bias) All outcomes	Low risk	No concerns over missing data
Selective reporting (reporting bias)	Low risk	Expected outcomes reported (NCT00000520).
Other bias	Low risk	The study appears to be free of other sources of bias.

**Table:** Nishihara 1988

***Study characteristics***		
Methods	Design: multicentre RCT Exclusions postrandomisation: 18 (clinical features and dose/duration of IVIG did not meet the criteria) Losses to follow‐up: none Intention‐to‐treat analysis: yes Duration of study: July 1984 to March 1988	
Participants	Country: Japan Setting: 13 paediatric clinics Number: 160 (intervention group A: n = 32; intervention group B: n = 42; intervention group C: n = 36; control* n = 32) Age (months, mean (SD)): intervention group A 17.7 (14.2); intervention group B 19.9 (14.4); intervention group C 19.6 (14.1); control 15.1 (8.6) Sex (M/F): data not provided Diagnostic criteria described: no Inclusion criteria: full clinical expression of KD and under 4 years of age, treatment started within 7 days after the onset Exclusion criteria: treatment given before the study	
Interventions	Primary or secondary KD treatment: primary Intervention group A: IVIG 50 to 100 mg/kg/day for 5 days Intervention group B: IVIG 200 mg/kg/day for 5 days Intervention group C: IVIG 400 mg/kg/day for 5 days Control*: ASA 30 mg/kg/day Concomitant medications: ASA 30 mg/kg/day	
Outcomes	CAA Fever Laboratory data Cardiac evaluation: 2‐dimensional ECG were done once a week for at least 3 weeks, including views of the right coronary artery and of the left main, anterior descending, and circumflex branches of the left coronary artery. The views were categorised into 3 depending on the largest diameter of the coronary artery at the third week of examination: score 0 (normal) for cases of a diameter < 3 mm; and score 2 (severe) for cases > 5.5 mm. The remaining participants scored 1 (mild). Time points: 1, 2, 3 weeks	
Funding	Not reported	
Declaration of interest	Not reported	
Notes	Data reported in a letter. *Patients treated in clinics with ASA were controls ‐ not randomised to treatment, so data from this group were not used in this review. IVIG: sulphonated human gammaglobulin or polyethylene‐glycol‐treated human gammaglobulin	
***Risk of bias***		
**Bias**	**Authors' judgement**	**Support for judgement**
Random sequence generation (selection bias)	Unclear risk	States randomised, but insufficient information to permit judgement of low risk or high risk
Allocation concealment (selection bias)	Unclear risk	Insufficient information to permit judgement of low risk or high risk
Blinding of participants and personnel (performance bias) All outcomes	Unclear risk	Insufficient information to permit judgement of low risk or high risk
Blinding of outcome assessment ‐ cardiac abnormalities (detection bias) All outcomes	Low risk	Quote "The views were interpreted blindly and independently"
Incomplete outcome data (attrition bias) All outcomes	Low risk	No concerns over missing data
Selective reporting (reporting bias)	Unclear risk	Results published as a letter. No other outcomes except for coronary abnormalities reported.
Other bias	Unclear risk	Results published as a letter, so likely not peer reviewed.

**Table:** Nonaka 1995

***Study characteristics***		
Methods	Design: RCT Exclusions postrandomisation: 9 (accessible administration of another drug) Losses to follow‐up: 1 Intention‐to‐treat analysis: no Duration of study: January 1991 to September 1994	
Participants	Country: Japan Setting: hospital Number: 90 (intervention n = 50; comparison n = 40) Age (months, mean (SD)): intervention 19.9 (16.4); comparison 25.3 (20.3) Sex (M:F): intervention 1.4:1; comparison 1.7:1 Diagnostic criteria described: no Inclusion criteria: children with KD with less than 8 days since onset Exclusion criteria: none described	
Interventions	Primary or secondary KD treatment: primary Intervention: IVIG 400 mg/kg for 3 days Comparison: prednisolone 2 mg/kg IV for 5 days then 2 mg/kg orally until CRP fell to negative Concomitant medications: ASA 50 mg/kg/day and dipyridamole 5 mg/kg/day (until CRP normalised). Both groups also received ASA 10 mg/kg/day and dipyridamole 5 mg/kg/day during admission, and ASA 5 mg/kg after discharge.	
Outcomes	Temperature Febrile period Laboratory data (CRP, serum albumin, total protein) CAL Cardiac evaluation: 2‐dimensional ECG at enrolment and follow‐up. CAA as defined by JKDRC (1 of: dilated lumen diameter of at least 3 mm in children < 5 years and at least 4 mm in children ≥ 5 years; internal diameter at least 1.5 times that of adjacent segment; or lumen clearly irregular) Time points: acute, 30 days, 3 months	
Funding	Not reported	
Declaration of interest	Not reported	
Notes	IVIG: no details	
***Risk of bias***		
**Bias**	**Authors' judgement**	**Support for judgement**
Random sequence generation (selection bias)	Unclear risk	States randomised, but insufficient information to permit judgement of low risk or high risk
Allocation concealment (selection bias)	Unclear risk	Insufficient information to permit judgement of low risk or high risk
Blinding of participants and personnel (performance bias) All outcomes	Unclear risk	No blinding of participants and personnel described.
Blinding of outcome assessment ‐ cardiac abnormalities (detection bias) All outcomes	High risk	No blinding of outcome assessment described, and the outcome measurement could have been influenced by lack of blinding.
Blinding of outcome assessment ‐ non‐cardiac outcomes (detection bias) All outcomes	High risk	No blinding of outcome assessment described, and the outcome measurement could have been influenced by lack of blinding.
Incomplete outcome data (attrition bias) All outcomes	Unclear risk	100 children randomised, but results only reported for 90 total, reasons not all clear.
Selective reporting (reporting bias)	Unclear risk	Expected outcomes reported, but no protocol.
Other bias	Unclear risk	Possible imbalance in age distributions between groups (13 to 24 months 30%:15% and 37 to 48 months 6%:17.5%, IVIG:prednisolone respectively)

**Table:** Ogawa 1987

***Study characteristics***		
Methods	Design: multicentre RCT Exclusions postrandomisation: 142 were randomised (25 cases were excluded from study analysis: 19 cases did not meet the inclusion criteria; 1 case in IVGG was high dose, 4 cases in IVGG were low dose, and 1 case in IVGG steroid was used) Losses to follow‐up: none Intention‐to‐treat analysis: no Duration of study: July 1985 to June 1986	
Participants	County: Japan Setting: 31 hospitals Number: 117 (intervention n = 63; comparison n = 54) Age (months, mean (SD)): intervention 27.8 (22.2); comparison 26.5 (21.8) Sex (M/F): intervention 35/28; comparison 38/16 Diagnostic criteria described: yes Inclusion criteria: treated for KD within 7 days of onset before treatment, no steroid or indometacin or IVIG use Exclusion criteria: not provided	
Interventions	Primary or secondary KD treatment: primary Intervention: IVIG (400 mg/kg/day for 3 consecutive days) plus ASA (30 mg/kg/day) Comparison: ASA (30 mg/kg/day, reduced to 10 mg/kg once a day after CRP‐negative) Concomitant medications: none reported	
Outcomes	CAL (1 to 10 days, 11 to 25 days, 30 days, 60 days after KD onset), frequency of occurrence of the 6 main symptoms (fever, rash, conjunctival hyperaemia, oral‐labial findings, cervical lymph node findings, extremity terminal changes), laboratory findings (CRP, ESR), side effects Cardiac evaluation: 2‐dimensional ECG (1 to 10 days, 11 to 25 days, 30 days, 60 days after KD onset). A coronary artery inner diameter of 3 mm or more, or 1.5 times the diameter of the surrounding coronary artery was considered abnormal. Aneurysm greater than 4 times the diameter of the surrounding coronary artery was defined as aneurysm large (ANI), greater than 1.5 times and less than 4 times the diameter of the surrounding coronary artery was defined as aneurysm middle (ANm), and less than 1.5 times the diameter of the surrounding coronary artery were defined as aneurysm small (ANs) or Dil.	
Funding	None reported. Quote "We thank Chemo‐Sero‐Therapeutic Research Institute, Fujisawa yakuhin and TelJin Ltd for donating S‐sulphonated gammaglobulin (Venilon)"	
Declaration of interest	Not reported	
Notes	IVIG: S‐sulphonated intact gammaglobulin; Venilon, Teijin Ltd, Japan This is study 2 of 3 reported by Ogino 1990. We requested additional information regarding randomisation and data. The author can no longer be contacted. The co‐author did not have any additional data.	
***Risk of bias***		
**Bias**	**Authors' judgement**	**Support for judgement**
Random sequence generation (selection bias)	Unclear risk	Reports groups were assigned at the direction of an independent controller, but no details provided
Allocation concealment (selection bias)	Low risk	States central randomisation by an independent controller
Blinding of participants and personnel (performance bias) All outcomes	Unclear risk	No blinding of participants or personnel described.
Blinding of outcome assessment ‐ cardiac abnormalities (detection bias) All outcomes	Unclear risk	States re‐examination by review committee, but no further details
Blinding of outcome assessment ‐ non‐cardiac outcomes (detection bias) All outcomes	Unclear risk	Insufficient information to permit judgement of low risk or high risk ‐ states re‐examination by review committee, but no further details
Incomplete outcome data (attrition bias) All outcomes	Unclear risk	6/123 participants were not included in analysis. These were all in the IVIG group (given a low dose). It was not clear if this could have impacted the results.
Selective reporting (reporting bias)	Unclear risk	Expected outcomes reported, but no protocol.
Other bias	Low risk	The study appears to be free of other sources of bias.

**Table:** Ogino 1987

***Study characteristics***		
Methods	Design: multicentre RCT Exclusions postrandomisation: 115 were randomised (23 cases were excluded from study analysis: 19 cases did not meet the inclusion criteria; 3 cases in IVGG were low dose, and 1 case in ASA used IVIG) Losses to follow‐up: none Intention‐to‐treat analysis: no Duration of study: May 1984 to April 1985	
Participants	County: Japan Setting: 33 hospitals Number: 92 (intervention n = 50; comparison n = 42) Age (months, mean (SD)): intervention 32.1 (24.5); comparison 27.6 (27.6) Sex (M/F): intervention 29/21; comparison 32/10 Diagnostic criteria described: yes Inclusion criteria: treated for KD within 7 days of onset, no steroid, indometacin, or IVIG used before treatment Exclusion criteria: recurrent or incomplete KD	
Interventions	Primary or secondary KD treatment: primary Intervention: IVIG (200 mg/kg/day for 3 consecutive days) plus ASA (30 mg/kg/day) Comparison: ASA (30 mg/kg/day, reduced to 10 mg/kg once a day after CRP‐negative) Concomitant medications: none	
Outcomes	CAL (1 to 10 days, 11 to 25 days, 30 days, 60 days after KD onset), frequency of occurrence of the 6 main symptoms (fever, rash, conjunctival hyperaemia, oral‐labial findings, cervical lymph node findings, extremity terminal changes), laboratory findings (CRP, ESR), side effects Cardiac evaluation: 2‐dimensional ECG (1 to 10 days, 11 to 25 days, 30 days, 60 days after KD onset). Defined as: a coronary artery inner diameter of 3 mm or more, or 1.5 times the diameter of the surrounding coronary artery was considered abnormal. An aneurysm greater than 4 times the diameter of the surrounding coronary artery was defined as aneurysm large (ANl), greater than 1.5 times and less than 4 times the diameter of the surrounding coronary artery was defined as aneurysm middle (ANm), and less than 1.5 times the diameter of the surrounding coronary artery was defined as aneurysm small (ANs) or Dil.	
Funding	None reported. Quote "We thank Chemo‐Sero‐Therapeutic Research Institute, Fujisawa yakuhin and TelJin Ltd for donating S‐sulphonated gammaglobulin (Venilon)"	
Declaration of interest	None reported.	
Notes	IVIG details: S‐sulphonated intact gammaglobulin; Venilon, Teijin Ltd, Japan This is study 1 of 3 reported in Ogino 1990. We requested additional information regarding randomisation and data. The author can no longer be contacted. The co‐author did not have any additional data.	
***Risk of bias***		
**Bias**	**Authors' judgement**	**Support for judgement**
Random sequence generation (selection bias)	Unclear risk	Reports groups were assigned at the direction of an independent controller, but no details provided.
Allocation concealment (selection bias)	Low risk	States central randomisation by an independent controller.
Blinding of participants and personnel (performance bias) All outcomes	Unclear risk	No blinding of participants or personnel described.
Blinding of outcome assessment ‐ cardiac abnormalities (detection bias) All outcomes	Unclear risk	States evaluation for CAL by independent review committee, but no further details.
Blinding of outcome assessment ‐ non‐cardiac outcomes (detection bias) All outcomes	Unclear risk	No description.
Incomplete outcome data (attrition bias) All outcomes	Low risk	4/96 participants not included in analysis, but reasons provided.
Selective reporting (reporting bias)	Unclear risk	Expected outcomes reported, but no protocol.
Other bias	Low risk	The study appears to be free of other sources of bias.

**Table:** Okuni 1987A

***Study characteristics***		
Methods	Design: multicentre RCT Exclusions postrandomisation: 3 Losses to follow‐up: 1 Intention‐to‐treat analysis: yes Duration of study: October 1983 to February 1985	
Participants	Country: Japan Setting: hospital Number: 214 (intervention n = 139 (pepsin treated n = 73, intact n = 66); comparison n = 75) Age (months, mean (SD)): intervention (pepsin treated 21.6 (14.7), intact 19.2 (15.4)); comparison 22.1 (15.0) Sex (M/F): intervention (pepsin treated 40/33, intact 36/30); comparison 47/28 Diagnostic criteria described: no Inclusion criteria: satisfied diagnostic criteria for KD, < 7 days KD onset, ≤ 4 years old Exclusion criteria: previously treated with indometacin or corticosteroids	
Interventions	Primary or secondary KD treatment: primary Intervention: IVIG 100 mg/kg single dose plus ASA 50 mg/kg/day until temperature < 37.5 ˚C, then 30 mg/kg/day Comparison: ASA 50 mg/kg/day until temperature < 37.5 ˚C, then 30 mg/kg/day Concomitant medications: none	
Outcomes	CAA (enrolment ‐ 60 days, and at 1 year if present at 60 days) Laboratory test (enrolment ‐ 60 days) Cardiac evaluation: 2‐dimensional ECG at enrolment and at least once a week in acute phase, then 30 to 60 days from onset. If CAA detected at 60 days, examined at 1 year after onset. Classified as normal, dilation and aneurysm "by the classification standard by Research Committee of Kawasaki Disease" (JMHW 1984)	
Funding	Quote "I gratefully acknowledge the support of the Kawasaki Disease Research Committee of Japan for this study" (from Harada 1991)	
Declaration of interest	Not reported	
Notes	IVIG: pepsin‐treated and intact gamma globulin used This study is the same as study 1 (of 3) reported in Harada 1991 (now merged).	
***Risk of bias***		
**Bias**	**Authors' judgement**	**Support for judgement**
Random sequence generation (selection bias)	Unclear risk	Quote "These patients randomly assigned to each treatment group, for the three stages of the study". States randomised, but insufficient information to permit judgement of low risk or high risk
Allocation concealment (selection bias)	Unclear risk	Insufficient information to permit judgement of low risk or high risk
Blinding of participants and personnel (performance bias) All outcomes	Unclear risk	No blinding of participants and personnel described. Unclear if this could have impacted outcomes
Blinding of outcome assessment ‐ cardiac abnormalities (detection bias) All outcomes	Low risk	ECGs recorded (stop motion/video) and interpreted by independent observers (from Harada 1991 report).
Blinding of outcome assessment ‐ non‐cardiac outcomes (detection bias) All outcomes	High risk	No description of blinding for assessment of any other outcome. Unclear if this could have affected other outcome reporting (from Harada 1991 report)
Incomplete outcome data (attrition bias) All outcomes	Low risk	No concerns over missing data, all accounted for
Selective reporting (reporting bias)	Unclear risk	Expected outcomes reported, but no protocol available.
Other bias	Low risk	The study appears to be free of other sources of bias.

**Table:** Okuni 1987B

***Study characteristics***		
Methods	Design: multicentre RCT Exclusions postrandomisation: none Losses to follow‐up: 18 Intention‐to‐treat analysis: yes Duration of study: February 1985 to March 1986	
Participants	Country: Japan Setting: hospital Number: 295 (intervention n = 196 (pepsin treated n = 96, intact n = 100); comparison n = 99) Age (months, mean (SD)): intervention (pepsin treated 21.0 (13.4), intact 17.7 (11.3)); comparison 18.5 (12.1) Sex (M/F): intervention (pepsin treated 63/33, intact 59/41); comparison 58/41 Diagnostic criteria described: no Inclusion criteria: satisfied diagnostic criteria for KD, < 7 days onset, ≤ 4 years old Exclusion criteria: previously treated with indometacin or corticosteroids	
Interventions	Primary or secondary KD treatment: primary Intervention: IVIG 100 mg/kg day for 5 days plus ASA 50 mg/kg/day until temperature < 37.5 ˚C, then 30 mg/kg/day Comparison: ASA 50 mg/kg/day until temperature < 37.5 ˚C, then 30 mg/kg/day Concomitant medications: none stated	
Outcomes	CAA (enrolment ‐ 60 days, and at 1 year if present at 60 days) Laboratory test (enrolment ‐ 60 days) Cardiac evaluation: 2‐dimensional ECG at enrolment and at least once a week in acute phase, then 30 to 60 days from onset. If CAA detected at 60 days, examined at 1 year after onset. Classified as normal, dilation and aneurysm "by the classification standard by Research Committee of Kawasaki Disease" (JMHW 1984)	
Funding	Quote "I gratefully acknowledge the support of the Kawasaki Disease Research Committee of Japan for this study" (from Harada 1991)	
Declaration of interest	Not reported	
Notes	IVIG: pepsin‐treated and intact gamma globulin used This study is the same as study 2 (of 3) reported in Harada 1991.	
***Risk of bias***		
**Bias**	**Authors' judgement**	**Support for judgement**
Random sequence generation (selection bias)	Unclear risk	Quote translated "These patients randomly assigned to each treatment group, for the three stages of the study" (from Harada 1991 report). States randomised, but insufficient information to permit judgement of low risk or high risk
Allocation concealment (selection bias)	Unclear risk	Insufficient information to permit judgement of low risk or high risk
Blinding of participants and personnel (performance bias) All outcomes	Unclear risk	No blinding of participants and personnel described. Unclear if this could have impacted outcomes
Blinding of outcome assessment ‐ cardiac abnormalities (detection bias) All outcomes	Low risk	ECGs recorded (stop motion/video) and interpreted by independent observers.
Blinding of outcome assessment ‐ non‐cardiac outcomes (detection bias) All outcomes	High risk	No description of blinding for assessment of any other outcome. Unclear if this could have affected other outcome reporting (from Harada 1991 report)
Incomplete outcome data (attrition bias) All outcomes	Low risk	No concerns over missing data
Selective reporting (reporting bias)	Unclear risk	Expected outcome of CAA reported, but no protocol available.
Other bias	Low risk	The study appears to be free of other sources of bias.

**Table:** Onouchi 1988

***Study characteristics***		
Methods	Design: multicentre RCT Exclusions postrandomisation: 22 Losses to follow‐up: 3 Intention‐to‐treat analysis: no Duration of study: April 1986 to April 1987	
Participants	Country: Japan Setting: 28 institutions Number: 126 (intervention group A: n = 38, group B: n = 42; comparison: n = 46) Age (months, mean (SD)): group A 22.2 (15.6), group B 23.4 (14.4); comparison 17.0 (12.0) Sex (M/F): group A (18/20), group B (26/16); comparison (32/14) Diagnostic criteria described: yes Inclusion criteria: patients with KD, < 5 years old, treatment initiated within 7 days of KD onset Exclusion criteria: steroid, indometacin, or IVIG prior to study treatment, recurrent disease	
Interventions	Primary or secondary KD treatment: primary Intervention group A: IVIG 200 mg/kg/day for 3 days with ASA 50 mg/kg/day until afebrile, then reduced to 30 mg/kg/day Intervention group B: IVIG 400 mg/kg/day for 3 days with ASA 50 mg/kg/day until afebrile, then reduced to 30 mg/kg/day Comparison: ASA 50 mg/kg/day until afebrile, then reduced to 30 mg/kg/day Concomitant medications: none stated	
Outcomes	Clinical symptoms (fever, rash, conjunctival congestion, oral cavity and lip findings, cervical lymph node swelling, abnormalities in hands and feet) CAL Laboratory tests (RBC, WBC, PLT, total protein, blood biochemistry, urinalysis) AE Cardiac evaluation: 2‐dimensional ECG of the left coronary artery main trunk, anterior descending branch, circumflex branch and right coronary artery at start of treatment, 15, 30, and 60 days after disease onset. Graded scale was either normal, dilated lesion or aneurysm (medium sized or large) "according to previously published standards (JMHW 1984)". Time points: enrolment, days 15, 30, and 60	
Funding	Not reported	
Declaration of interest	Not reported	
Notes	IVIG: reduced alkylated human IG; Polyglobulin Bayer Yakuhin, Ltd	
***Risk of bias***		
**Bias**	**Authors' judgement**	**Support for judgement**
Random sequence generation (selection bias)	Unclear risk	States randomised, but sequence appears to have been decided by rule based on hospital visit order using alternate envelope allocation and so not truly random. Quote "The controller assigned one case to each treatment for a total of three cases comprising a single set." Controllers randomly assigned a set of different medication.
Allocation concealment (selection bias)	Unclear risk	In the order of receipt of eligible patients, open the envelopes from the lowest number and administer the prescriptions listed inside.
Blinding of participants and personnel (performance bias) All outcomes	Unclear risk	No blinding of participants and personnel described.
Blinding of outcome assessment ‐ cardiac abnormalities (detection bias) All outcomes	Low risk	Quote "CAL evaluations were conducted by a committee who based their evaluation on recorded 2DE videotapes. They were blind to treatment involved"
Blinding of outcome assessment ‐ non‐cardiac outcomes (detection bias) All outcomes	Low risk	Non‐cardiac outcomes assessments were performed by independent members.
Incomplete outcome data (attrition bias) All outcomes	Unclear risk	More participant data available for AE (145) than for cardiac evaluation (126), exclusions described.
Selective reporting (reporting bias)	Unclear risk	Expected outcomes reported, but no protocol.
Other bias	Unclear risk	Some baseline imbalances between groups reported (more females and lower mean age in the ASA group).

**Table:** Onouchi 1992

***Study characteristics***		
Methods	Design: multicentre RCT Exclusions postrandomisation: 9 Losses to follow‐up: 4 Intention‐to‐treat analysis: no Duration of study: October 1990 to September 1991	
Participants	Country: Japan Setting: 39 institutions Number: 160 (group A (n = 53); group B (n = 51); group C (n = 56)) Age (months, mean (SD)): group A 21.0 (15.8); group B 20.1 (16.3); group C 23.3 (16.8) Sex (M/F): group A 31/22; group B 30/21; group C 30/26 Diagnostic criteria described: yes Inclusion criteria: patients with KD, < 5 years old, treatment initiated within 7 days of KD onset Exclusion criteria: steroid, indometacin, or IVIG prior to study treatment, recurrent disease	
Interventions	Primary or secondary KD treatment: primary Intervention group A: IVIG 100 mg/kg/day for 5 days with ASA 30 mg/kg/day Intervention group B: IVIG 200 mg/kg/day for 5 days with ASA 30 mg/kg/day Intervention group C: IVIG 400 mg/kg/day for 5 days with ASA 30 mg/kg/day Concomitant medications: none stated	
Outcomes	Clinical symptoms (fever, rash, conjunctival congestion, oral cavity and lip findings, cervical lymph node swelling, abnormalities in hands and feet) CAL Laboratory tests (RBC, WBC, PLT, total protein, blood biochemistry, urinalysis) AE Cardiac evaluation: 2‐dimensional ECG of the left coronary artery main trunk, anterior descending branch, circumflex branch and right coronary artery at start of treatment, 15, 30, and 60 days after disease onset. Graded scale was either normal, dilated lesion or aneurysm (medium‐sized or large) "according to previously published standards (JMHW 1984)". Time points: enrolment, days 15, 30, and 60	
Funding	Not reported	
Declaration of interest	Not reported	
Notes	IVIG: pH4 stabilised acid human IG, Polyglobulin N‐Bayer Yakuhin Ltd	
***Risk of bias***		
**Bias**	**Authors' judgement**	**Support for judgement**
Random sequence generation (selection bias)	Unclear risk	States randomised centrally, but unclear if sequence was decided by physician. Quote "Controllers randomly assigned a set of three different medication doses"
Allocation concealment (selection bias)	Low risk	Controllers randomly assigned a set of 3 different medication doses. The investigators opened the envelope in the order of enrolment of eligible participants, starting with the lowest numbered envelope, and administered the listed prescriptions.
Blinding of participants and personnel (performance bias) All outcomes	Unclear risk	No blinding of participants and personnel described.
Blinding of outcome assessment ‐ cardiac abnormalities (detection bias) All outcomes	Low risk	Quote "CAL evaluations were conducted by a committee who based their evaluation on recorded 2DE videotapes. They were blind to treatment involved"
Blinding of outcome assessment ‐ non‐cardiac outcomes (detection bias) All outcomes	Low risk	Non‐cardiac outcomes assessments were performed by independent members.
Incomplete outcome data (attrition bias) All outcomes	Low risk	9/169 participants not included in analysis, but reasons provided.
Selective reporting (reporting bias)	Unclear risk	Expected outcomes reported, but no protocol.
Other bias	Low risk	The study appears to be free of other sources of bias.

**Table:** Qin 2006

***Study characteristics***		
Methods	Design: RCT Exclusions postrandomisation: none described Losses to follow‐up: 93.4% followed up to 12 months Intention‐to‐treat analysis: unclear Duration of study: July 2000 to October 2005	
Participants	Country: China Setting: hospital Number: 242 (intervention n = 120; comparison n = 122) Age (mean (SD)): mean 4.0 (2.8) years (range 3 months to 14 years; 83.1% < 5 years) Sex (M:F): 1.66:1; intervention 69/51; comparison 82/40 Diagnostic criteria described: yes Inclusion criteria: children with KD. Typical cases meet the KD diagnostic criteria revised by the 3rd International Kawasaki Disease Conference, atypical cases meet at least 3 to 4 main clinical manifestations including fever and CAD formally confirmed by ECG, age < 14 years. Exclusion criteria: irregular ASA or IVIG treatment prior to admission, disease duration > 10 days, KD combined with other congenital heart malformations	
Interventions	Primary or secondary KD treatment: primary (within 7 to 10 days of illness) Intervention: IVIG 2 g/kg single IV injection over 8 to 12 hours Comparison: IVIG 1 g/kg single IV injection over 8 to 12 hours Concomitant medications: ASA 80 to 100 mg/kg/d, reduced once fever controlled; if the participant has CAD, ASA continued until diameter normal	
Outcomes	CAL, fever, clinical symptoms, WBC, PLT, serum IgG, CRP, ESR, ECG Cardiac evaluation: unclear Time points: enrolment and 2 weeks (follow‐up range 4 months to 5.4 years)	
Funding	National Natural Science Foundation of China	
Declaration of interest	None reported.	
Notes	Translated Lyophilised low pH human blood IVIG	
***Risk of bias***		
**Bias**	**Authors' judgement**	**Support for judgement**
Random sequence generation (selection bias)	Unclear risk	Translation: randomly divided into 2 groups according to gender, age, stage of the disease, with/without CAL, with/without high‐risk factors States randomised, but insufficient information to permit judgement of low risk or high risk
Allocation concealment (selection bias)	Unclear risk	Insufficient information to permit judgement of low risk or high risk
Blinding of participants and personnel (performance bias) All outcomes	Unclear risk	No blinding of participants and personnel described.
Blinding of outcome assessment ‐ cardiac abnormalities (detection bias) All outcomes	High risk	No blinding of outcome assessment described, and the outcome measurement could have been influenced by lack of blinding.
Blinding of outcome assessment ‐ non‐cardiac outcomes (detection bias) All outcomes	High risk	No blinding of outcome assessment described, and the outcome measurement could have been influenced by lack of blinding.
Incomplete outcome data (attrition bias) All outcomes	Unclear risk	Insufficient information to permit judgement of low risk or high risk
Selective reporting (reporting bias)	Unclear risk	Insufficient information to permit judgement of low risk or high risk
Other bias	Low risk	No evidence of other bias

**Table:** Sakata 2007

***Study characteristics***		
Methods	Design: multicentre RCT Exclusions postrandomisation: none described Losses to follow‐up: none Intention‐to‐treat analysis: yes Duration of study: January 2000 to December 2002	
Participants	Country: Japan Setting: hospital Number: 109 (intervention n = 54; comparison n = 55) Age (months, mean (SD)): 28.0 (21.0); intervention 28.89 (19.73); comparison 27.25 (21.95) Sex (M/F): 61/48 Diagnostic criteria described: no Inclusion criteria: KD admitted before day 7 of onset, additional IVIG administered when patient showed 2 or 3 criteria 24 to 36 hours after previous IVIG dose: body temperature > 37.5 ˚C, CRP > 3.0 mg/dL and neutrophils ≥ 7500/mm^3^ Exclusion criteria: CAL at time of admission, treated with other drugs during the clinical course	
Interventions	Primary or secondary KD treatment: primary with additional therapy Intervention: IVIG 2 g/kg single infusion (additional therapy 2 g/kg) Comparison: IVIG 1 g/kg single infusion (1st additional therapy 1 g/kg, and 2nd 2 g/kg) Concomitant medications: ASA 30 mg/kg/day, reduced to 10 mg/kg/day once body temperature ≤ 37.5 ˚C, further reduced to 5 mg/kg/day when CRP ≤ 0.5 mg/dL. ASA was continued for at least 2 months, even if no symptoms of CAL. Flurbiprofen administered in participants with liver dysfunction.	
Outcomes	Total dose of IG CAL Plasma IG concentration Cardiac evaluation: serial UCG days 5, 7, 10, 14, 21, and 28, of coronary arteries. CAL defined as intact (no morphological change in coronary arteries during clinical course); transient dilation (transient dilated coronary arteries regressed to their normal size with 1 month of illness); aneurysm (diameter of right or left coronary artery > 4 mm or 1.5 times that or coronary orifice at 1 month within onset of illness). If both transient dilation and aneurysm, then regarded as CAL. Abstract reports serial ECG until day 60, no further details.	
Funding	Not reported	
Declaration of interest	Not reported	
Notes	IVIG: Venilon (Teijin Pharma, Japan), Venoglobulin‐IH (Venesis, Japan) or Polyglobin‐N (Bayer Yakuhin, Japan), choice institute‐dependent	
***Risk of bias***		
**Bias**	**Authors' judgement**	**Support for judgement**
Random sequence generation (selection bias)	Low risk	Quote "a randomization code was generated using a random number table such that the number of patients assigned to each protocol group was almost similar"
Allocation concealment (selection bias)	Low risk	Quote "Randomization to either protocol was performed with sequentially numbered, sealed envelopes using the previously determined code."
Blinding of participants and personnel (performance bias) All outcomes	Low risk	Quote "The clinicians treating the patients were blinded to the protocol."
Blinding of outcome assessment ‐ cardiac abnormalities (detection bias) All outcomes	Low risk	Quote "The UCG was performed by the same paediatric cardiologists, and the findings recorded on a video were evaluated by more than four paediatric cardiologists."
Blinding of outcome assessment ‐ non‐cardiac outcomes (detection bias) All outcomes	Unclear risk	No blinding of outcome assessment described, but total Ig and plasma Ig unlikely to be influenced by lack of blinding.
Incomplete outcome data (attrition bias) All outcomes	Low risk	No concerns over missing data
Selective reporting (reporting bias)	Unclear risk	Expected outcomes reported, but no protocol.
Other bias	Low risk	The study appears to be free of other sources of bias.

**Table:** Sato 1995

***Study characteristics***		
Methods	Design: RCT Exclusions postrandomisation: none Losses to follow‐up: none Intention‐to‐treat analysis: yes Duration of study: January 1991 to December 1995	
Participants	Country: Japan Setting: hospital Number: 145 (intervention n = 72; comparison n = 73) Age (months, mean (SD)): intervention 23.9 (21.7); comparison 22.0 (17.6) Sex (M/F): intervention 46/26; comparison 47/27 Diagnostic criteria described: yes Inclusion criteria: patients with KD, 9 days of onset, scoring ≥ 4 on Harada score Exclusion criteria: ≤ 3 on Harada score	
Interventions	Primary or secondary KD treatment: primary Intervention: IVIG 2 g/kg single treatment over 12‐hour infusion Comparison: IVIG 400 mg/kg/day for 5 days over 2‐ to 3‐hour infusions Concomitant medications: ASA 30 mg/kg/day in acute febrile phase and 5 mg/kg/day when afebrile	
Outcomes	Primary outcome: CAA Secondary outcomes: fever clinical symptoms laboratory data (CRP, WBC, PLT, albumin) AE number of hospital days total medical costs Cardiac evaluation: 2‐dimensional ECG (at admission, day 4/5, and discharge) of main trunk, anterior descending and circumflex branches of the left coronary artery and the main and distal segments of the right coronary artery. Graded as aneurysmal if the internal lumen diameter was at least 3 mm in children < 5 years or at least 4 mm in children ≥ 5 years; or if the internal diameter was at least 1.5 times that of an adjacent segment. If CAA indicated, coronary angiograms were taken in the convalescent phase. Time points: enrolment, day 4/5 and 7 (approximately discharge)	
Funding	Not reported	
Declaration of interest	Not reported	
Notes	Harada score used, those ≤ 3 on Harada score did not receive IVIG, only ASA (not randomised) IVIG: S‐sulphonated human IG (Kenketsu Venilon‐I, Kaketsuken, Japan)	
***Risk of bias***		
**Bias**	**Authors' judgement**	**Support for judgement**
Random sequence generation (selection bias)	Low risk	Quote "We randomly assigned participating patients to treatment groups by means of a stratified randomization according to age (<1 year vs 1 year) and gender (male vs female). Using this random number table..."
Allocation concealment (selection bias)	Unclear risk	Insufficient information to permit judgement of low risk or high risk
Blinding of participants and personnel (performance bias) All outcomes	High risk	No blinding of participants and personnel
Blinding of outcome assessment ‐ cardiac abnormalities (detection bias) All outcomes	Low risk	Quote "Each patient’s echocardiogram was recorded on a single videotape and reviewed by pediatric cardiologists who were unaware of patient identity"
Blinding of outcome assessment ‐ non‐cardiac outcomes (detection bias) All outcomes	High risk	No blinding of outcome assessment described, and the outcome measurement could have been influenced by lack of blinding.
Incomplete outcome data (attrition bias) All outcomes	Low risk	No concerns about missing data
Selective reporting (reporting bias)	Unclear risk	Expected outcomes reported, but no protocol.
Other bias	Unclear risk	Baseline differences reported in age and blood tests between IVIG and no IVIG group.

**Table:** Wang 2020

***Study characteristics***		
Methods	Design: RCT Exclusions postrandomisation: none Losses to follow‐up: 47* Intention‐to‐treat analysis: yes Duration of study: January 2018 to June 2019	
Participants	Country: China Setting: hospital Number: 80 (intervention n = 40; comparison n = 40) Age (months, mean (SD)): intervention 21.0 (14.4); comparison 25.6 (21.1) Sex (M/F): intervention 28/11; comparison 28/13 Diagnostic criteria described: yes Inclusion criteria: diagnosed and hospitalised KD patients initially treated with IVIG then assessed as IVIG resistant (recrudescent or persistent fever at least 36 hours after first IVIG infusion) Exclusion criteria: presence of other diseases that affect temperature in the course of KD (sepsis, influenza, and juvenile idiopathic arthritis), absence of detailed information about initial treatment outside the hospital, prior history of KD, treatment with hormone or immunosuppressant therapy in preceding 30 days, presence of severe immune disease (immunodeficiency or chromosomal abnormality), refusal to sign informed consent, inability to follow up for at least 6 months	
Interventions	Primary or secondary KD treatment: secondary Intervention: IVIG 2 g/kg single infusion Comparison: MPT 15 mg/kg/day IV for 3 days Concomitant medications: ASA 30 to 50 mg/kg/day; all participants initially treated with IVIG 2 g/kg and ASA 30 to 50 mg/kg/day within the first 10 days of fever onset	
Outcomes	Primary outcomes: CAL Secondary outcomes: duration of fever after retreatment length of hospital stay medical costs readmission rate laboratory data (CRP, WBC, haemoglobin, PLT, albumin, sodium) AE Cardiac evaluation: maximal Z‐scores from ECG assessment of luminal dimensions are categorised as follows: no involvement: Z‐scores always < 2; dilation only: Z‐score ≥ 2 but < 2.5; small aneurysm: Z‐score ≥ 2.5 to < 5; medium aneurysm: Z‐score ≥ 5 to < 10, and absolute dimension < 8 mm; giant aneurysm: Z‐score ≥ 10, or absolute dimension ≥ 8 mm. Time points: enrolment, 48 hours (fever), 1 week (laboratory data, CAL), 1, 3, 6, 12, 24 months (CAL)	
Funding	Quote "This study was supported by the Guangdong Natural Science Fund, China (grant number 2016A030313836), and the Guangdong Science and Technology Project of China (grant number 2017A030223003)"	
Declaration of interest	Quote "The authors declare no competing financial interests"	
Notes	*As no CAL detected, participants did not return to hospital for further checkups.	
***Risk of bias***		
**Bias**	**Authors' judgement**	**Support for judgement**
Random sequence generation (selection bias)	Low risk	Quote "Non‐participating nurses generated the random tables using a computer. Once eligible subjects had entered the trial, the nurses assigned the groups to trial researchers for clinical trial intervention"
Allocation concealment (selection bias)	Low risk	Quote "Once eligible subjects had entered the trial, the nurses assigned the groups to trial researchers for clinical trial intervention"
Blinding of participants and personnel (performance bias) All outcomes	High risk	No blinding of participants and personnel
Blinding of outcome assessment ‐ cardiac abnormalities (detection bias) All outcomes	High risk	No blinding of outcome assessment, and the outcome measurement could have been influenced by lack of blinding (strict diagnosis criteria of CAL described, but still high risk as may not prevent bias)
Blinding of outcome assessment ‐ non‐cardiac outcomes (detection bias) All outcomes	High risk	No blinding of outcome assessment described, and the outcome measurement could have been influenced by lack of blinding.
Incomplete outcome data (attrition bias) All outcomes	Low risk	No concerns over missing data
Selective reporting (reporting bias)	Low risk	Expected outcomes reported as planned in protocol (ChiCTR‐EOC‐17013266).
Other bias	Unclear risk	Although randomisation procedure described, protocol states this is a cohort study, without randomisation.

**Table:** Yabiku 1989

***Study characteristics***		
Methods	Design: multicentre RCT Exclusions postrandomisation: 124 were randomised (40 cases were excluded from study analysis: 32 cases did not meet the inclusion criteria, 8 cases in IVIG group were given low dose) Losses to follow‐up: none Intention‐to‐treat analysis: no Duration of study: October 1986 to February 1988	
Participants	Country: Japan Setting: 33 hospitals Number: 84 (intervention n = 48; comparison n = 36) Age (months, mean (SD)): intervention 21.4 (18.1); comparison 24.9 (15.0) Sex (M/F): intervention 29/19; comparison 18/18 Diagnostic criteria described: yes Inclusion criteria: treated for KD within 7 days of onset, no steroid, indometacin, or IVIG use before treatment Exclusion criteria: recurrent or incomplete KD	
Interventions	Primary or secondary KD treatment: primary Intervention: IVIG (1000 mg/kg/day single dose) plus ASA (30 mg/kg/day) Comparison: ASA (30 mg/kg/day, reduced to 10 mg/kg once a day after CRP‐negative) Concomitant medications: no	
Outcomes	CAL (1 to 10 days, 11 to 25 days, 30 days, 60 days after KD onset), frequency of occurrence of the 6 main symptoms (fever, rash, conjunctival hyperaemia, oral‐labial findings, cervical lymph node findings, extremity terminal changes), laboratory findings (CRP, ESR), side effects Cardiac evaluation: 2‐dimensional ECG (1 to 10 days, 11 to 25 days, 30 days, 60 days after KD onset). Defined as: coronary artery inner diameter of 3 mm or more, or 1.5 times the diameter of the surrounding coronary artery was considered abnormal. Aneurysm greater than 4 times the diameter of the surrounding coronary artery was defined as aneurysm major (ANI), greater than 1.5 times and less than 4 times the diameter of the surrounding coronary artery was defined as aneurysm moderate (ANm), and less than 1.5 times the diameter of the surrounding coronary artery was defined as aneurysm minor (ANs) or Dil.	
Funding	None reported.	
Declaration of interest	None reported.	
Notes	IVIG: S‐sulphonated intact gammaglobulin; Venilon, Teijin Ltd, Japan This is study 3 of 3 reported in Ogino 1990 Progress in Medicine. We requested additional information regarding randomisation and data. The author can no longer be contacted. The co‐author did not have any additional data.	
***Risk of bias***		
**Bias**	**Authors' judgement**	**Support for judgement**
Random sequence generation (selection bias)	Unclear risk	Reports groups were assigned at the direction of an independent controller, but no details provided
Allocation concealment (selection bias)	Low risk	States central randomisation by an independent controller
Blinding of participants and personnel (performance bias) All outcomes	Unclear risk	No description
Blinding of outcome assessment ‐ cardiac abnormalities (detection bias) All outcomes	Unclear risk	There were insufficient details on blinding provided, but study reports that the final evaluation for CAL was carried out by an independent review committee.
Blinding of outcome assessment ‐ non‐cardiac outcomes (detection bias) All outcomes	Unclear risk	No description of blinding provided.
Incomplete outcome data (attrition bias) All outcomes	Unclear risk	8/90 participants were not included in analysis. These were all in the IVIG group (given a low dose). It was not clear if this may have impacted the results.
Selective reporting (reporting bias)	Unclear risk	Expected outcomes reported, but no protocol.
Other bias	Unclear risk	Imbalance in numbers across intervention (8 participants in IVIG group removed from analysis, no dropouts in ASA group)

**Table:** Youn 2016

***Study characteristics***		
Methods	Design: multicentre RCT Exclusions postrandomisation: none Losses to follow‐up: none Intention‐to‐treat analysis: no Duration of study: January 2010 to June 2015	
Participants	Country: Korea Setting: hospital Number: 43 (intervention n = 32; comparison n = 11) Age (range): 3 months to 13 years (data per group not given, but states no baseline differences) Sex (M/F): 28/15 Diagnostic criteria described: initial KD no, retreatment indicated according to institutional KD guidelines Inclusion criteria: persistent or recrudescent fever ≥ 38.0 ˚C beyond 36 to 48 hours after completion of initial IVIG infusion according to institutional guideline Exclusion criteria: none given	
Interventions	Primary or secondary KD treatment: secondary Intervention: IVIG 2 g/kg Comparison: infliximab 5 mg/kg, paracetamol (15 mg/kg orally), and chlorpheniramine (2 mg or 4 mg IV) were administered 30 minutes before infliximab infusion Concomitant medications: initial treatment was IVIG 2 g/kg plus ASA 80 to 100 mg/kg/d for 2 to 8 days after the onset of disease	
Outcomes	Need for further treatment Duration of fever elapsed from completion of first retreatment to defervescence (< 37.5 ˚C) Total days of hospitalisation CAL AE Laboratory data (WBC, PLT counts, aspartate aminotransferase, alanine aminotransferase, protein, albumin, CRP, and N‐terminal pro‐brain natriuretic peptide) Cardiac evaluation: ECG at entry, 1 to 2 weeks, 6 to 8 weeks after retreatment. CAL determined by the criteria of Japanese Ministry of Health and Welfare (JMHW 1984). Changes from baseline were not calculated. Time points: enrolment, day 7, and 1, 3, 6, 12, 24 months	
Funding	Not reported.	
Declaration of interest	Quote "The authors have no funding or conflicts of interest to disclose"	
Notes	Imbalance in group numbers "...associated with patient selection according to the change in standard practice for patients with IVIG resistant KD in our institution during the study period"	
***Risk of bias***		
**Bias**	**Authors' judgement**	**Support for judgement**
Random sequence generation (selection bias)	Unclear risk	States randomised, but insufficient information to permit judgement of low risk or high risk
Allocation concealment (selection bias)	Unclear risk	Insufficient information to permit judgement of low risk or high risk
Blinding of participants and personnel (performance bias) All outcomes	Unclear risk	No blinding of participants or personnel described.
Blinding of outcome assessment ‐ cardiac abnormalities (detection bias) All outcomes	High risk	No blinding of outcome assessment, and the outcome measurement could have been influenced by lack of blinding. Quote "...echocardiograms were not read blindly"
Blinding of outcome assessment ‐ non‐cardiac outcomes (detection bias) All outcomes	High risk	No blinding of outcome assessment described, and the outcome measurement could have been influenced by lack of blinding.
Incomplete outcome data (attrition bias) All outcomes	Low risk	No concerns over missing data
Selective reporting (reporting bias)	Unclear risk	Expected outcomes reported, but no protocol.
Other bias	Unclear risk	Numbers imbalanced between groups, and mention of a change to standard practice for IVIG‐resistant KD during study which caused this.

**Table:** Yuan 2000

***Study characteristics***		
Methods	Design: CCT, a parallel study Exclusions postrandomisation: unclear Losses to follow‐up: not reported Intention‐to‐treat analysis: unclear Duration of study: March 1996 to March 1998	
Participants	Country: China Setting: hospital Number: 50 (intervention n = 25; comparison n = 25) Age (months, mean and range): intervention 45 (8 to 90); comparison 53 (9 to 84) Sex (M/F): intervention 13/12; comparison 15/10 Diagnostic criteria described: yes Inclusion criteria: patients were admitted to the paediatric department in hospital between March 1996 and March 1998 and met the clinical diagnostic criteria for KD Exclusion criteria: not specified	
Interventions	Primary or secondary KD treatment: primary Intervention: 1 g/kg/d IVIG for 2 days Comparison: IVMP 2 mg/kg/d for 5 days then oral prednisolone until CRP‐negative Concomitant medications: ASA 50 mg/kg/d, oral dipyridamole if CAA detected	
Outcomes	Duration of acute symptoms (fever, conjunctival congestion, swelling of hands and feet, swollen lymph nodes in the neck), laboratory indices, CAA Cardiac evaluation: ECG and echocardiography at 2 weeks, 3, 6, and 9 months, 1 year post‐treatment. Assessment of CAA not clear (subjectively described) Time points: 2 weeks, 3, 6, 9, and 12 months	
Funding	Not reported	
Declaration of interest	Not reported	
Notes	IVIG details: not reported	
***Risk of bias***		
**Bias**	**Authors' judgement**	**Support for judgement**
Random sequence generation (selection bias)	High risk	Not truly random ‐ used odd/even hospital numbers (quasi‐RCT)
Allocation concealment (selection bias)	High risk	Allocation not described, and likely to be insufficient because of alternation used in sequence generation (quasi‐RCT).
Blinding of participants and personnel (performance bias) All outcomes	Unclear risk	No blinding of participants or personnel described.
Blinding of outcome assessment ‐ cardiac abnormalities (detection bias) All outcomes	High risk	No blinding of outcome assessment described, and the outcome measurement could have been influenced by lack of blinding.
Blinding of outcome assessment ‐ non‐cardiac outcomes (detection bias) All outcomes	High risk	No blinding of outcome assessment described, and the outcome measurement could have been influenced by lack of blinding.
Incomplete outcome data (attrition bias) All outcomes	Unclear risk	Unclear if all data provided
Selective reporting (reporting bias)	Unclear risk	Unclear what the outcomes were and how these were defined
Other bias	Low risk	The study appears to be free of other sources of bias.

2DE: 2‐dimensional echocardiography AE: adverse events AHA: American Heart Association ALT: alanine transaminase ASA: acetylsalicylic acid BCG: Bacillus Calmette–Guérin CAA: coronary artery aneurysm CAD: coronary artery dilation CAL: coronary artery lesions CCT: controlled clinical trial CRP: C‐reactive protein ECG: echocardiogram ESR: erythrocyte sedimentation rate Ig: immunoglobulin IgA: immunoglobulin A IgE: immunoglobulin E IgG: immunoglobulin G IgM: immunoglobulin M IQR: interquartile range ITT: intention‐to‐treat IV: intravenous IVGG: intravenous gamma globulin IVIG: intravenous immunoglobulin IVMP: intravenous methylprednisolone JKDRC: Japanese Kawasaki Disease Research Committee JMHW: Japanese Ministry of Health and Welfare KD: Kawasaki disease MPT: methylprednisolone pulse therapy PLT: platelet RBC: red blood cell RCT: randomised controlled trial SD: standard deviation TB: tuberculosis TNF: tumour necrosis factor UCG: ultrasonographic cardiogram VGIH: polyethylene glycol‐treated human immunoglobulin WBC: white blood cell

### Characteristics of excluded studies [ordered by study ID]

**Table:**

Study	Reason for exclusion
ChiCTR2000035163	Ongoing study to investigate KD treatment in participants either IVIG‐resistant or predicted to be IVIG‐resistant. IVIG not the differentiating intervention
Hamada 2019	Study compared IVIG plus ciclosporin versus IVIG alone in people predicted to be at higher risk for IVIG resistance. IVIG not the differentiating intervention
ISRCTN71987471	Participants randomised to corticosteroid or nothing after initial IVIG plus ASA treatment.
JPRN‐UMIN000014665	Study compared fast versus slow injection of IVIG.
Lee 1996	Study only reported surrogate outcomes (lymphocyte phenotype), so does not meet our inclusion criteria.
Lin 2021	Study plans to evaluate the efficacy of IVIG plus ASA plus prednisolone compared to IVIG plus ASA in Chinese paediatric participants with KD.
Muta 2002	Study compared different types of IVIG, and dosage was determined by KD severity.
Nanishi 2017	Study compared IVIG with IVIG plus clarithromycin.
NCT02298062	Clinical trial report states completed September 2015, but no published report identified. We contacted the study investigators to request data (Fang Liu, Professor, Fudan University, liufang@fudan.edu.cn; January 2022). They replied to say that study was not completed due to limited participants and COVID‐19.
Portman 2019	Study randomised participants to either etanercept or placebo after initial dose of IVIG.
RAISE 2012	Study compared IVIG plus ASA versus IVIG plus prednisolone.
Sanati 2021	Study compared IVIG versus IVIG plus ASA.
Seki 2021	Study compared different speeds of IVIG infusion.

ASA: acetylsalicylic acid (aspirin)  IVIG: intravenous immunoglobulin KD: Kawasaki disease

### Characteristics of studies awaiting classification [ordered by study ID]

**Table:** Chang 2007

Methods	
Participants	
Interventions	
Outcomes	
Notes	Translation required. Unable to verify if meets our inclusion criteria

**Table:** Chang 2008

Methods	
Participants	
Interventions	
Outcomes	
Notes	Translation required. Unable to verify if meets our inclusion criteria

**Table:** Chen 2006

Methods	
Participants	
Interventions	
Outcomes	
Notes	Translation required. Unable to verify if meets our inclusion criteria

**Table:** Fu 2010

Methods	
Participants	
Interventions	
Outcomes	
Notes	Translation required. Unable to verify if meets our inclusion criteria

**Table:** Ho 2003

Methods	
Participants	
Interventions	
Outcomes	
Notes	Translation required. Unable to verify if meets our inclusion criteria

**Table:** Ho 2004

Methods	
Participants	
Interventions	
Outcomes	
Notes	Translation required. Unable to verify if meets our inclusion criteria

**Table:** Juan 2003

Methods	
Participants	
Interventions	
Outcomes	
Notes	Translation required. Unable to verify if meets our inclusion criteria

**Table:** Juan 2005

Methods	
Participants	
Interventions	
Outcomes	
Notes	Translation required. Unable to verify if meets our inclusion criteria

**Table:** Juan 2006

Methods	
Participants	
Interventions	
Outcomes	
Notes	Translation required. Unable to verify if meets our inclusion criteria

**Table:** Kuo 2009

Methods	
Participants	
Interventions	
Outcomes	
Notes	Translation required. Unable to verify if meets our inclusion criteria

**Table:** Li 2008

Methods	
Participants	
Interventions	
Outcomes	
Notes	Translation required. Unable to verify if meets our inclusion criteria

**Table:** Li 2009

Methods	
Participants	
Interventions	
Outcomes	
Notes	Translation required. Unable to verify if meets our inclusion criteria

**Table:** Liao 2007

Methods	
Participants	
Interventions	
Outcomes	
Notes	Translation required. Unable to verify if meets our inclusion criteria

**Table:** Liu 2000

Methods	
Participants	
Interventions	
Outcomes	
Notes	Translation required. Unable to verify if meets our inclusion criteria

**Table:** Liu 2004

Methods	
Participants	
Interventions	
Outcomes	
Notes	Translation required. Unable to verify if meets our inclusion criteria

**Table:** Liu 2009

Methods	
Participants	
Interventions	
Outcomes	
Notes	Full text required. Unable to verify if meets our inclusion criteria

**Table:** Lu 2003

Methods	
Participants	
Interventions	
Outcomes	
Notes	Translation required. Unable to verify if meets our inclusion criteria

**Table:** Peng 2001

Methods	
Participants	
Interventions	
Outcomes	
Notes	Translation required. Unable to verify if meets our inclusion criteria

**Table:** Teng 2005

Methods	
Participants	
Interventions	
Outcomes	
Notes	Translation required. Unable to verify if meets our inclusion criteria

**Table:** Yao 2009

Methods	
Participants	
Interventions	
Outcomes	
Notes	Full text required. Unable to verify if meets our inclusion criteria

**Table:** Yen 2007

Methods	
Participants	
Interventions	
Outcomes	
Notes	Translation required. Unable to verify if meets our inclusion criteria

**Table:** Yuan 2009

Methods	
Participants	
Interventions	
Outcomes	
Notes	Translation required. Unable to verify if meets our inclusion criteria

**Table:** Yueh 2006

Methods	
Participants	
Interventions	
Outcomes	
Notes	Translation required. Unable to verify if meets our inclusion criteria

### Characteristics of ongoing studies [ordered by study ID]

**Table:** ChiCTR1900027954

Study name	Efficacy of infliximab on IVIG‐unresponsive KD
Methods	Single‐centre prospective randomised controlled study
Participants	Inclusion criteria Children diagnosed as KD according to the AHA statement Children with persistent fever (axillary temperature ≥ 38 ˚C for no less than 4 h) after 36 h since the initial IVIG therapy (2 g/kg, or 1 g/kg x 2 days) within 4 to 10 days from onset of KD Age and gender unlimited Axillary temperature ≥ 37.5 ˚C at enrolment The use of infliximab within 10 days from onset Exclusion criteria Contraindications for the use of infliximab Not IVIG as the initial therapy of KD Use of systemic steroids within 4 weeks History of treatment with infliximab or other biological agents Live vaccination within 1 month
Interventions	Intervention: infliximab Comparison: second dose of IVIG
Outcomes	Primary outcomes Febrile duration after treatment Defervescent rate within 48 h after treatment Reintervention rate Incidence of coronary artery lesions Secondary outcomes White blood cells C‐reactive protein TNF‐a IL‐6, IL‐8, IL‐10
Starting date	1 January 2020
Contact information	Yanfei Wang Guangzhou Women and Children's Medical Center, Guangzhou, Guangdong, China Email: wangyanfei@gwcmc.org
Notes	Funding: self‐financing

**Table:** EUCTR2020‐003194‐22‐FR

Study name	A randomized phase III multicentre trial comparing the efficacy and safety of Anakinra versus intravenous immunoglobulin (IVIG) retreatment, in patients with Kawasaki disease who failed to respond to initial standard IVIG treatment (ANACOMP)
Methods	Randomised, controlled, parallel, open‐label trial to compare the efficacy of anakinra (IL‐1R1 receptor antagonist) with second‐line IVIG infusion on fever in children with KD who failed to respond to 1 infusion of IVIG (standard treatment)
Participants	Inclusion criteria Children, male and female, from 3 months to < 18 years old Child's weight ≥ 5 kg Child with KD according to the AHA definition for complete or incomplete KD Children who failed to respond to the standard therapy of KD, e.g. persistence or recrudescence of fever ≥ 38 ˚C, 48 h after the infusion of 2 g/kg of IVIG Children may be screened 24 h after the end of the first infusion if they remain febrile 24 h after the end of the first infusion Child, parents', or legal guardian’s written informed consent is required Efficient contraception for the duration of participation in the research for childbearing‐aged women Exclusion criteria Preterm and neonates, pregnancy and breastfeeding Suspicion of another diagnosis Other concomitant bacterial, viral, or fungal infection Previously treated with steroids and/or another biotherapy Increased risk of TB infection or recent TB infection or with active TB Any type of immunodeficiency or cancer Severe renal impairment Hepatic insufficiency/neutropenia Included in another interventional protocol Undergoing the following treatments (paracetamol, NSAIDs other than ASA 30 to 50 mg/kg given for purpose of KD inflammation, systemic steroids, ciclosporin, tacrolimus, azathioprine, cyclophosphamide, interferon, mycophenolate, other anti‐IL‐1, anti‐IL‐6, anti CD20, and anti‐TNF), plasmapheresis Hypersensitivity to anakinra or excipients, hypersensitivity to IVIG, hypersensitivity to human normal immunoglobulin Hyperprolinaemia Live vaccines within 1 month prior to enrolment Hypersensitivity to anakinra or immunoglobulins or excipients of Kineret or Privigen or to *Escherichia coli* proteins Contraindication for administration of anakinra or IVIG listed in SmPC of Kineret and Privigen
Interventions	Intervention: IL‐1R receptor antagonist (Kineret/anakinra 100 mg/0.67 mL) Comparison: human normal immunoglobulin (Privigen 100 mg/mL)
Outcomes	Primary outcome The participant must reach a body (axillary (+0.5 ˚C), tympanic, oral) temperature < 38 ˚C within 2 days after initiation of treatment (i.e. a binary outcome: success/failure) Secondary outcomes Efficacy on fever at 72 h Efficacy on disease activity Efficacy on KD symptoms Efficacy on coronary lesions (e.g. dilatation and aneurysm) Efficacy on inflammation Safety and tolerability
Starting date	December 2021
Contact information	Malika Yahmi Hopital Saint‐Louis, Paris, France Email: malika.yahmi@aphp.fr Isabelle Koné‐Paut CHU de Bicêtre, Le Kremlin‐Bicêtre, France Email: isabelle.kone‐paut@aphp.fr
Notes	Funding: Ministère de la Santé ANACOMP Study NCT04656184

AHA: American Heart Association IL: interleukin IL‐1R: interleukin‐1 receptor IVIG: intravenous immunoglobulin KD: Kawasaki disease NSAIDs: non‐steroidal anti‐inflammatory drugs SmPC: summary of product characteristics TB: tuberculosis TNF‐a: tumour necrosis factor‐alpha

## Differences between protocol and review

### 2022 version

We amended the Types of studies section to clarify that we excluded studies that did not investigate or report any of our outcomes of interest.

We amended the Types of interventions section to clarify that we would not include studies that compared the speed of intravenous immunoglobulin (IVIG) infusion or a particular type of IVIG versus another (i.e. manufacturer or heparinated/sulphonated). We originally planned to undertake subgroup analysis to investigate the efficacy of IVIG produced by different manufacturers. Following discussion, we determined that this was not clinically relevant and it has been removed.

We had planned to use a fixed‐effect model when there were no concerns about heterogeneity. As there were many differences between studies (doses, infusion regimen, concomitant medications, etc.), we used a random‐effects model for all analyses, not only in the case of substantial heterogeneity (I^2^ > 50%).

We had planned to undertake subgroup analyses by initial or refractory (secondary) IVIG treatment and by single‐ or multiple‐dose regimens. Given the range of regimens involved in the included studies, it was clinically more appropriate to present initial versus secondary IVIG treatment, and single versus multiple doses as separate comparisons and subgrouped by the total dose administered. For the same reason, we presented analyses for all outcomes in this way, not just our primary outcomes as planned at protocol stage. We did not investigate adverse effects by subgroup for geographical distribution as, given the results of the main analysis, this would not have provided any useful information. We carried out additional sensitivity analyses with studies at high risk of detection bias for cardiac outcomes, as coronary artery abnormalities was our most clinically relevant outcome, and for studies where it was not clear what Kawasaki disease diagnostic criteria were used, as this may have impacted the results. We undertook further sensitivity analysis when we identified substantial heterogeneity by removing studies from the analyses to assess the individual impact of studies on the results.
